# Steric Mapping, Ligand Dynamics, and Cycloisomerization Catalysis with Redox Robust Mn^I/0/‑I^ Dicarbenes

**DOI:** 10.1021/acs.organomet.6c00057

**Published:** 2026-04-27

**Authors:** Viani Maxwell, Ageliki Karagiannis, Tim K. Schramm, Veronika Kotelnikow, Rupal Gupta, Roger A. Lalancette, Aaron M. Appel, Andreas Hansen, Eric S. Wiedner, Demyan E. Prokopchuk

**Affiliations:** † Department of Chemistry, 242612 Rutgers University, Newark, New Jersey 07102, United States; ‡ Mulliken Center for Theoretical Chemistry, Clausius Institute for Physical and Theoretical Chemistry, Rhenish Friedrich Wilhelms University of Bonn, Bonn 53115, Germany; § Department of Chemistry and Biochemistry, College of Staten Island, City University of New York, 2800 Victory Blvd., Staten Island, Staten Island, New York 10314, United States; ∥ Institute for Integrated Catalysis, 6865 Pacific Northwest National Laboratory, P.O. Box 999, Richland, Washington 99352, United States

## Abstract

Manganese is perhaps the most electronically versatile element, yet the redox properties, reactivity, and catalytic applications of low-valent Mn^0^/Mn^–I^ complexes remain underexplored due to the propensity for Mn^0^ to dimerize, quenching high-energy metalloradicals. We report a series of redox-active monometallic Mn^I^, Mn^0^ and Mn^–I^ complexes containing a BH_2_-bridged dicarbene, characterized using a suite of experimental and cutting-edge computational (DFT) methods. Slow electron transfer kinetics at Mn^I/0^ are observed, with computations and electrochemical simulations in excellent agreement with experimental values. Despite the lack of steric bulk at the BH_2_-bridged Mn^0^, the ^t^Bu groups at the dicarbene provide adequate steric protection to prevent dimerization, with percent buried volume (%*V*_bur_) serving as a valuable steric ranking tool. We also show that a %*V*_bur_ > 83% prevents dimerization for a diverse array of Mn^0^ complexes from the literature. Ligand sterics of BPh_2_-and BH_2_-bridged complexes dictate reaction outcomes when Mn^I^ and Mn^–I^ are exposed to nucleophiles and electrophiles, respectively, while Mn^0^ facilitates the radical cycloisomerization catalysis of 6-iodo-1-hexene at room temperature. This work underscores the importance of ligand sterics in rationalizing reactivity patterns at Mn and provides valuable insights for designing chelating ligands that can selectively leverage Mn^I/0/‑I^ states in redox-mediated catalytic reactions.

## Graphical abstract

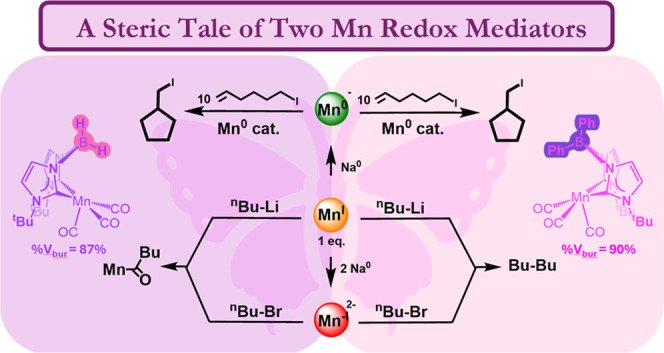

## Introduction

Manganese is renowned for its capability to access formal oxidation states ranging from −3 ([Mn­(CO)_4_]^3–^)^1,2^ to +7 ([MnO_4_]^2–^),^3,4^ making it a unique Earth-abundant metal for its sheer electronic breadth. In between these extremes, higher valent monometallic examples include Mn^V^ to Mn^III^ porphyrinoid complexes,^5,6^ Mn^III^X_3_(OPPh_3_)_2_ oxidants,^7,8^ and Mn^II^ complexes as initiators for olefin polymerization^9–12^ and precatalysts for the C–H bond oxygenation of organic substrates.^13–17^ Moreover, Mn^I^ compounds are well-established for their foundational carbonyl insertion/elimination chemistry^18–20^ and myriad of catalytic (de)­hydrogenation applications.^21–29^ The Mn^–I^ anion [Mn­(CO)_5_]^−^ participates in decarbonylation reactions^18^ and compounds with the general formula [L_2_Mn­(CO)_3_]^−^ are key intermediates in electrocatalytic CO_2_ reduction to CO and/or HCOO^–^ (L_2_ = chelating bidentate ligand).^30–37^

While transient Mn^0^ metalloradical intermediates have been utilized for catalysis via electrochemical,^38^ photochemical,^37,39^ and thermal^40–44^ activation pathways, Mn^0^ complexes that are kinetically stable under standard-state conditions are relatively rare due to the propensity of Mn^0^ systems to dimerize^30,31,34,37,45–48^ or perform stoichiometric atom abstraction reactions.^49,50^ Despite these challenges, a small collection of isolable monometallic Mn^0^ metalloradicals has been reported, albeit with a menagerie of ligand types such as aryl isocyanide,^51,52^ indenyl,^53^ *N*-heterocyclic carbene (NHC),^47,54,55^ and pincer-type phosphine/silylene.^56,57^ Previously, we reported a strongly reducing, mononuclear Mn^0^ anion stabilized by a bulky borate-bridged dicarbene ligand, which was subsequently reduced by 1e^–^ to yield an isolable and incredibly potent Mn^–I^ dianion with a redox potential of −3.13 V vs Fc^+/0^ in THF.^54,55^ Additionally, these Mn^0^ and Mn^–I^ dicarbene complexes can release 1e^–^ or 2e^–^, respectively, easily toggling between the +1, 0, and −1 formal oxidation states in the presence of oxidants.^55^

The diverse set of ligand frameworks supportive of Mn^0^ systems makes metalloradical stability predictions nontrivial. While it is reasonable to argue that metalloradical stability in the above examples is conferred by steric protection, an outstanding question remains: is there an approximate steric threshold that prevents Mn–Mn bond formation and subsequent radical–radical quenching? To harness the capacity of Mn^0^ as a powerful electron reservoir, it stands to reason that additional research is required to understand the underlying principle(s) behind the stabilization of Mn^0^ radicals and predict the stability of such systems as a function of the ligands’ primary and secondary coordination spheres. Moreover, the strongly reducing properties of Mn^–I^ systems present additional redox stability challenges, which further hamper their isolation and analysis,^53,58,59^ prompting us to explore the interconversion of Mn^0/‑I^ states for potential applications in redox mediation,^60^ small molecule activation,^61^ flow batteries,^62,63^ and organic electrosynthesis.^64–66^

Herein, we report the synthesis, electrochemistry, spectroscopy, and computational (DFT) analysis of a redox-active tricarbonyl manganese complex incorporating the borate-bridged NHC ligand H_2_B­(^t^BuNHC)_2_, which differs from its predecessor by the replacement of the phenyl groups at boron with hydrogen atoms (Figure 1).^67^ Despite the decrease in steric protection of the metal center in Mn­(CO)_3_(H_2_B­(^t^BuNHC)_2_) (1^H^), a 1e^–^ reduction also generates the rare mononuclear Mn^0^ metalloradical [2^H^]^−^. To better understand this unexpected outcome, we undertook a percent buried volume (%*V*_bur_) analysis of [2^H^]^−^ and other reported Mn^0^ complexes,^68–70^ which predicts that a minimum steric threshold of approximately 83% is required to avoid dimer formation across a diverse set of coordination environments. A second reduction of [2^H^]^−^ leads to a Mn^–I^ species ([3^H^]^2–^), which is also isolable and incredibly reducing (*E*_1/2_ = −2.85 V, THF). The benchmarked, DFT-computed redox potentials and linearly scaled IR stretching frequencies for the Mn^I/0/‑I^ series are in overall excellent agreement with experimental values. Exposure of the BPh_2_-and BH_2_-containing Mn complexes (1^Ph^ and 1^H^) to organolithium or Grignard reagents reveals divergent reactivity, with the more sterically bulky analogue 1^Ph^ facilitating C–C homocoupling, while 1^H^ forms a novel acylmanganate­(I) salt that undergoes rapid intramolecular acyl exchange in solution (Δ*G* ^‡^_exp_ = 13.1 kcal/mol, Δ*G* ^‡^_DFT_ = 12.6 kcal/mol) facilitated by a reversible μ–BH interaction. The remarkable stability of Mn^0^ anions [2^H^]^−^ and [2^Ph^]^−^ enables them to facilitate the radical cycloisomerization catalysis of 6-iodo-1-hexene under mild conditions (room temperature), with the spent Mn^I^ adducts being easily recovered in 40% and 99% yields, respectively. The observed redox chemistry and reactivity patterns with small molecules are exquisitely controlled by the steric bulk at boron, providing key insights into the role of the secondary coordination sphere in ligand design for governing electron transfer and organoradical reactivity with redox-active Mn systems.

**Figure 1:**
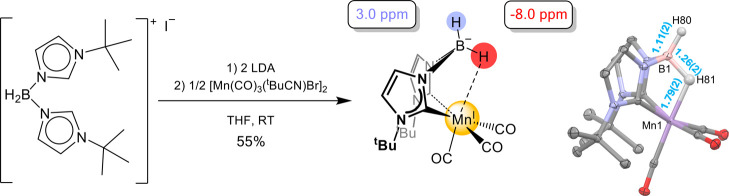
Left: synthesis of 1^H^. Right: molecular structure of 1^H^ with 50% probability ellipsoids and most hydrogen atoms removed for clarity. Experimental bond lengths are shown in blue. RT: room temperature; LDA: lithium diisopropylamide.

## Results and Discussion

Synthesis of H_2_B­(^tBu^NHC)_2_Mn­(CO)_3_ (1^H^) was achieved in a one-pot reaction by deprotonating the previously known iodide salt [H_2_B­(^tBu^Im)_2_]­I^71^ with lithium diisopropylamide (LDA) and adding [MnBr­(CO)_3_(NC^t^Bu)]_2_,^72^ yielding orange X-ray quality crystals after workup. The molecular structure reveals a close interaction between the Mn center and one of the hydrogens at the boron bridge (H···Mn = 1.79(2) Å). Inclusion of this μ–BH interaction within the metal’s coordination sphere provides additional stability to the formally 16e^–^ valence count at Mn (Figure 1) and is reminiscent of the interaction between the Mn center and the ipso carbon of the phenyl ring in Ph_2_B­(^tBu^NHC)_2_Mn­(CO)_3_ (1^Ph^).^54^

Careful analysis of NMR spectral data was critical to establish that the B–H bond of 1^H^ remains intact but weakened, as terminal MnH­(CO)_3_(dicarbene) complexes can have Mn–H bond lengths ranging from 1.63(3) – 1.79(3) Å.^36,73^ The μ–BH interaction in 1^H^ is static under ambient conditions, allowing one of the two hydrogen atoms to appear as a sharp doublet at −7.99 ppm (^2^ *J*_HH_ = 13 Hz) in ^1^H­{^11^B} NMR spectra (CD_2_Cl_2_) at room temperature, while the nonbridging hydrogen resonates at 3.00 ppm (^2^ *J*_HH_ = 13 Hz; Figure S7).^74^ This shielding effect resembles three-center two-electron agostic (CH–M) interactions,^75,76^ where electron donation from σ_BH_ to Mn^I^ is coupled with electron backdonation from Mn^I^ into σ*_BH_,^74^ weakening the B–H bond. Boron-coupled ^1^H NMR data collection results in severe peak broadening at room temperature, with B–H signals appearing as 1:1:1:1 quartets with ^1^ *J*_HB_ = 65 Hz and ^1^ *J*_HB_ = 124 Hz for the bridging and terminal hydrogen atoms, respectively (Figure S8). The substantial B–H bond lengthening for the μ-BH moiety (1.26(2) Å) is consistent with other bidentate ligands with B–H-M bridges^74^ and a decreased ^1^ *J*_HB_ indicates B–H bond weakening, mirroring decreased ^1^ *J*_CH_ values observed in agostic (CH–M) interactions.^75,76^ Of note, a similar μ–BH interaction with a borate-bridged chelating bis­(benzothiazole) complex has been previously reported (Mn···H = 1.71(2) Å; δ = −11.09 ppm (^1^H)).^77^

We hypothesized that the decreased steric profile at the borate bridge of 1^H^ would lead to dimerization upon 1e^–^ reduction, but to our surprise, 1^H^ can be smoothly reduced to the monometallic Mn^0^ radical anion ([2^H^]^−^) in the presence of 1.1 equiv KC_8_ or sodium naphthalenide (NaNap), followed by addition of 2,2,2-cryptand (Figure 2A). Single crystals suitable for X-ray diffraction were obtained in both cases, and the structure of [K­(2,2,2­(crypt))]­[2^H^] is shown in Figure 2B. The crystal structure shows that the strong μ–BH interaction is significantly weakened, as the Mn···H distance increases by about 1.4 Å (Mn···H = 3.16(2) Å). This Mn···H distance is about 0.3 Å shorter than the Mn---C_ipso_ distance of [K­(2,2,2­(crypt))]­[2^Ph^], which is in line with decreased Pauli (electronic) repulsion between the half-filled *d*_z2_ orbital at Mn and the proximal B-R moiety in [2^H^]^−^ compared to [2^Ph^]^−^ with larger substituents at boron.^54^

**Figure 2:**
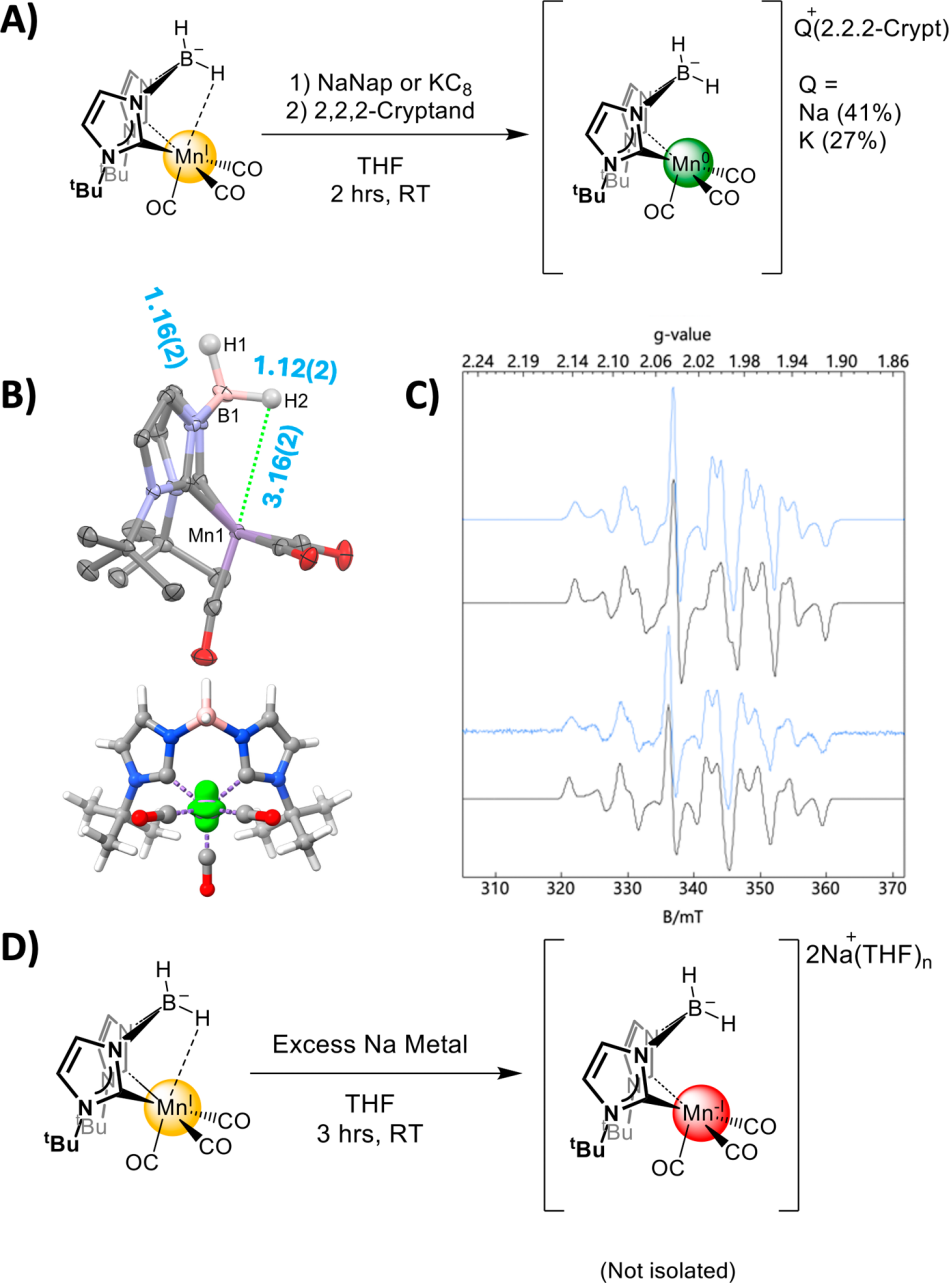
(A): synthesis of [2^H^]^−^ using KC_8_ or NaNap (Nap = naphthalenide). (B): molecular structure of [K­(2,2,2­(crypt))]­[2^H^] with 50% probability ellipsoids along with a spin density plot at PW6B95-D4/def2-QZVPPD/COSMO-RS­(THF)//B3LYP-3c/CPCM­(THF) level of theory (isovalue of 0.03 Å^–3^). The countercation and most hydrogens are omitted for clarity, and the spin density plot reveals localization of radical character on the Mn^0^ center. C: Comparison of EPR spectra of [2^Ph^]^−^ (top) and [2^H^]^−^ (bottom). Black traces are simulations to the experimental spectra with parameters as provided in the main text. Experimental conditions: microwaves, 0.2 mW at 9.65 GHz; temperature, 77 K. D: Synthesis of [Na­(THF)_*x*_]_2_[3^H^].

To better understand the electronic differences between [2^H^]^−^ and the known metalloradical [2^Ph^]^−^, we acquired X-band EPR spectral data on [2^H^]^−^ and [2^Ph^]^−^ (Figure 2C). While the steric profiles at boron are markedly different between these two samples, the EPR spectral data of [2^H^]^−^ and [2^Ph^]^−^ are nearly identical. The X-band EPR spectrum of [2^H^]^−^ (Figure 2C) indicates a rhombic electronic symmetry for the *d* ^7^, S = 1/2 Mn(0) center with numerical simulations yielding a *g*-tensor = (2.030, 2.023, 2.007) and hyperfine tensor, A = ± (148, 217, 135) MHz. The EPR spectrum of the previously reported complex [2^Ph^]^−^ exhibits electronic parameters *g* = (2.025, 2.019, 1.998) and A = ± (152, 214, 118) MHz; Figure 2C), which are similar to [2^H^]^−^. This suggests that the electronic environment of the Mn^0^ center is not dramatically influenced by the absence of phenyl groups on the boron moiety and that the radical character of the complex is primarily localized on the metal center. The latter is also supported by the calculated spin densities of both Mn^0^ complexes, confirming a metal-centered reduction and thus spin localization on the metal (Figure S44). Furthermore, EPR spectra provide insight into the similarities and differences between the two complexes as briefly described below. Similar magnitudes of the g_⊥_ and A_⊥_ tensorial components of the two complexes suggest that the phenyl group introduces limited perturbations in the pseudo equatorial plane of the Mn(0) center. However, the A_||_ component of the hyperfine tensors of the two complexes (A_||_^[2H]‑^ = 135 MHz; A_||_^[2Ph]^ = 118 MHz), shows notable variation compared to the A_⊥_ component. This variation between the two complexes along the electronic *z*-axes is consistent with the reduced distance (−0.3 Å) for the axial H moiety in [2^H^]^−^ compared to the Mn---C_ipso_ distance in [2^Ph^]^−^. These differences are also reflected in g_||_ values where g_||_ < 2 for the [2^Ph^]^−^ complex, originating from second-order correction afforded by the electronic d_*xz*/yz_ → d_z2_ transition (suppressed in [2^H^]^−^). Lastly, the isotropic hyperfine interactions (*A*_iso_) calculated from the experimentally determined A-tensor of the two complexes are 161 ([2^Ph^]^−^) and 167 ([2^H^]^−^) MHz. In previous EPR studies of inorganic Mn complexes, *A*_iso_ values were observed to increase with a decrease in oxidation state.^78^ Based on this, an *A*_iso_ value greater than that of typical Mn­(II) centers (∼250 MHz) may be expected for a Mn^0^ radical. Instead, we observe *A*_iso_ values of the Mn^0^ radical complexes reported herein that align more closely to the *A*_iso_ value of an *S* = 1 Mn­(V) complex (*A*_iso_ = 163 MHz).^79^ This suppression of the Fermi contact term in our *d* ^7^ [2^H^]^−^ and [2^Ph^]^−^ complexes may arise from the cancellation of net spin polarization at the nucleus from doubly occupied d-orbitals, thus making the overall contact interaction similar to that of a *d* ^2^ Mn­(V) ion. We note that this net loss of spin polarization may also originate due to the transfer of spin density to ligand π-orbitals resulting in increased covalency of the metal–ligand bonds. Additional studies further investigating the origin of suppressed *A*_iso_ values are ongoing, but beyond the scope of this work.

Complex 1^H^ is incredibly robust under strongly reducing conditions, as reactions with excess solid Na^0^ in THF afford the Mn^–I^ dianion [Na­(THF)_*x*_]_2_[3^H^], which has been characterized in solution at room temperature (Figure 2D). This complex is also isolable under the same rigorous air-free conditions previously used for [3^Ph^]^2–^.^55^ Additionally, NMR spectral characterization of [3^H^]^2–^ in THF-*d*_8_ reveals more similarities to [3^Ph^]^2–^ with a comparable ^11^B chemical shift at −7.87 ppm (cf. −5.40 ppm for [3^Ph^]^2–^) and chemically equivalent ^13^C resonances for the CO ligands trans to the NHCs (Figure S17). The ^1^H NMR spectrum of [3^H^]^2–^ reveals that the protons on boron appear at 2.56 and 3.27 ppm, indicating the absence of a Mn----H interaction for the (formally 18 e^–^) Mn^–I^ center (Figure S15). Performing ^11^B-decoupled ^1^H NMR on [3^H^]^2–^ does not resolve these peaks into sharp doublets as previously observed for 1^H^.

In situ infrared IR spectral analysis of 1^H^ using a ReactIR apparatus clearly shows the downward shift of the three CO stretching frequencies by ca. 120 cm^–1^ per electron added as the metal center is successively reduced with excess NaK alloy and 2 equiv 18-crown-6 (see Figure 3).^58,59^ The experimental IR spectral data are provided in Table 1. With experimental IR data for the 2e^–^ reductions of both 1^H^ and 1^Ph^ in hand, we turned to density functional theory calculations to accurately reproduce the diagnostic IR bands of the carbonyl ligands, using the ORCA^80^ software package. B3LYP-3c/CPCM­(THF) showed excellent agreement with the experimental IR data (Table 1), while also efficiently yielding molecular structures, thus motivating its use throughout this study.

**Figure 3:**
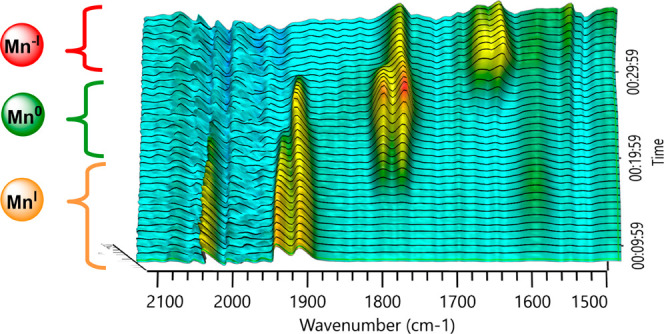
ReactIR spectrum of 1^H^ in THF in the presence of excess NaK alloy and 2 equiv 18-crown-6, showing the CO stretching frequency region for the sequential reduction of 1^H^ to [2^H^]^−^ to [3^H^]^2–^. The time axis is in minutes.

**Table 1:** Experimental and Computed^a^ IR Data for the Carbonyl Moieties in the Hydrogen- and Phenyl-Substituted Complexes^55^ With Varying Oxidation States

complex	*v* (CO, asym.) (cm^–1^)	*v* (CO, sym.) (cm^–1^)	*v* (CO, sym.) (cm^–1^)
1^H^	2017 (2011)	1928 (1926)	1902 (1899)
[2^H^]^−^	1909 (1901)	1802 (1803)	1776 (1777)
[3^H^]^2–^	1781 (1783)	1673 (1691)	1655 (1652)
1^Ph^	2014 (2012)	1932 (1927)	1887 (1888)
[2^Ph^]^−^	1909 (1901)	1812 (1807)	1775 (1774)
[3^Ph^]^2–^	1775 (1784)	1671 (1699)	1656 (1652)

a The corresponding linearly scaled frequency values as computed at the B3LYP-3c/CPCM­(THF) level of theory are given in parentheses. See Section III of the Supporting Information for details on obtaining the linear scaling factors for B3LYP-3c and other selected methods.^81,82^

Thus, far, EPR and IR spectroscopic analyses have shown that the electronic structures of 1^Ph^ and 1^H^ are very similar. Next, the voltammetric responses of 1^H^ under reducing conditions were explored in detail. Interestingly, we find that the Mn^I/0^ electron transfer kinetics of 1^H^/[2^H^]^−^ deviate substantially from 1^Ph^/[2^Ph^]^−^ (Figure 4A). Sweeping in the cathodic direction in either THF or MeCN reveals two redox events via cyclic voltammetry (CV) centered at −2.02 V and −2.90 V vs Fc^+/0^ in THF for the Mn^I/0^ and Mn^0/‑I^ redox events, respectively. There is also an irreversible solvent-dependent oxidation feature at +0.471 V and +0.618 V in THF and MeCN, respectively, suggesting that further oxidation to Mn^II^ is accompanied by CO loss and subsequent solvent coordination.^83^ Most notably, the peak-to-peak separation (Δ*E*_p_) for the Mn^I/0^ redox couple of 1^H^ is substantially larger than that of the Mn^0/‑I^ couple at a similar scan rate (υ) of 100 mV/s (Δ*E*_p_(Mn^I/0^) = 270 mV).

**Figure 4:**
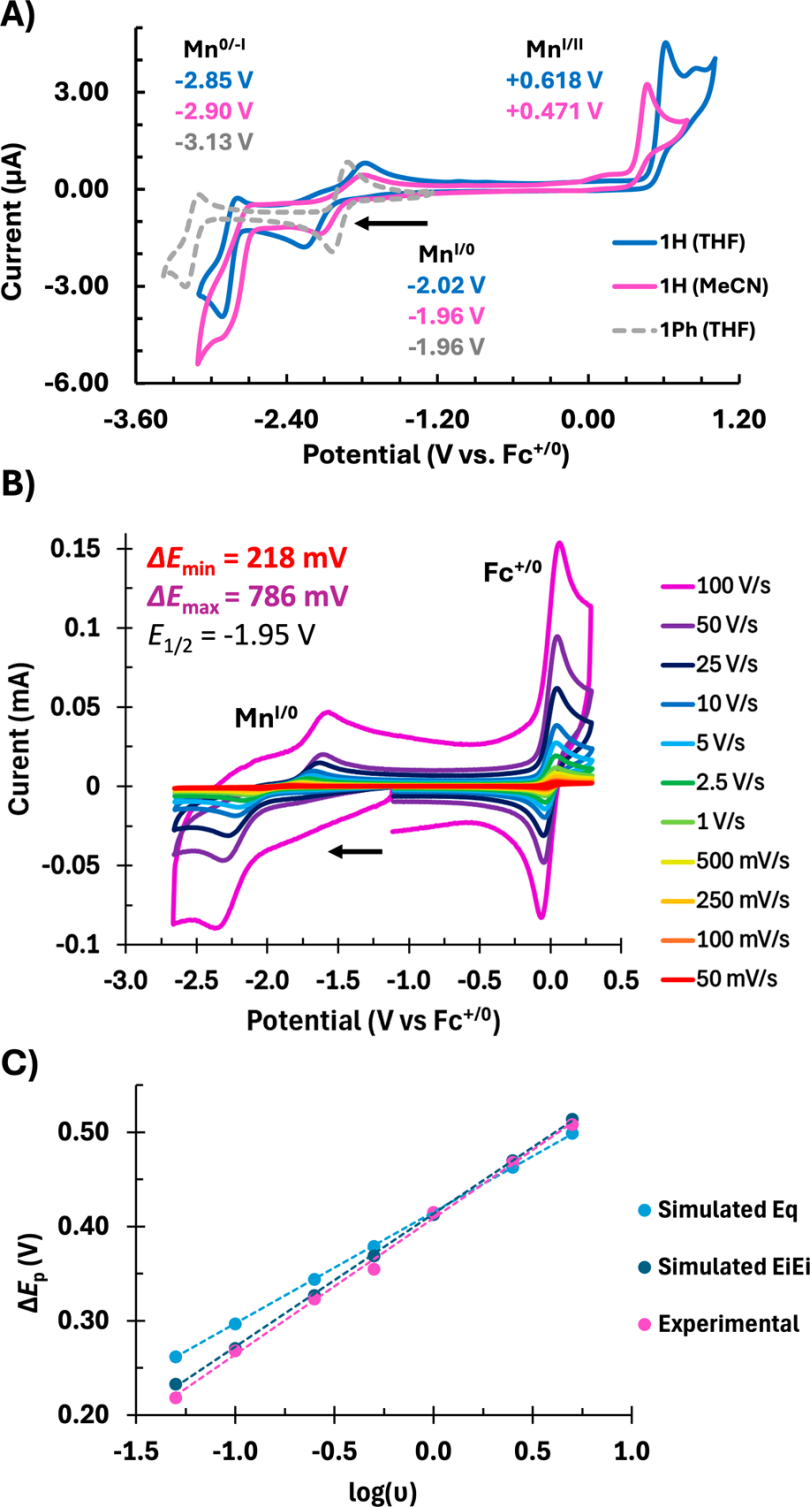
(A): CVs of 1^H^ in THF and MeCN, showing reversible Mn^I/0^ and Mn^0/‑I^ redox events. For comparison, the CV trace for 1^Ph^ in THF^55^ is shown in gray. (B): Variable scan rate CVs of the Mn^I/0^ redox couple in THF with added Fc, showing increased Δ*E*_p_ as a function of scan rate. Voltammograms are IUPAC plotted and referenced vs Fc^+/0^. Conditions: N_2_ atmosphere, 1 mM analyte, 0.1 M [^n^Bu_4_N]­[PF_6_] electrolyte, PEEK-encased glassy carbon working electrode, Type 2 glassy carbon rod counter electrode, Ag wire pseudoreference electrode coated with AgCl. Initial scan direction and position are indicated by a black arrow. (C): Plot of Δ*E*_p_ versus log­(υ) for the experimental Mn^I/0^ couple and the simulated *E*_Q_ and *E*_*I*_ *E*_I_ models.

Variable scan rate CVs on the Mn^I/0^ couple and ferrocene (Figure 4B) clearly show that while Δ*E*_p_ remains nearly constant for ferrocene, Δ*E*_p_ for Mn^I/0^ increases up to 786 mV at a scan rate of 100 V/s. A plot of the peak potentials versus log­(υ) affords slopes of −74 mV and 71 mV for the cathodic and anodic processes, respectively. These values are significantly larger than the slope of 29.6 mV, which would be expected for a kinetically limiting chemical reaction,^84,85^ indicating that the Mn^I/0^ redox process is limited by slow electron transfer kinetics. Digital simulations were used to further model the electron transfer kinetics of the scan-rate dependent CVs, in which the Mn­(I/0) couple was modeled by two different electron transfer processes – a single quasireversible electron transfer (*E*_Q_), and two separate irreversible electron transfers (*E*_I_ *E*_I_) for the reduction and oxidation processes (full details of the simulations are provided in part A of the SI). As can be seen in Figure 4C, the *E*_I_ *E*_I_ model provides a better match with the experimentally measured Δ*E*_p_ values, suggesting that the slow electron transfer is coupled to significant structural reorganization of the Mn coordination sphere. In contrast to the rapid electron transfer observed for the BPh_2_-substituted predecessor 1^Ph^/[2^Ph^]^−^, these data suggest that the μ-BH---Mn interaction is significantly stronger than the ipso-C_arene_---Mn interaction.^54^

In light of the slow electron transfer kinetics, we turned to computational modeling with density functional theory to better understand how the observed redox potentials of 1^H^/[2^H^]^−^ correlate with the structural reorganization. To achieve this, we first assessed the accuracy of 40 functional and solvation model combinations for the calculation of the Mn^I/0^ redox potentials of 1^H^ and 1^Ph^ in MeCN and THF, yielding PW6B95-D4/def2-QZVPPD/COSMO-RS//B3LYP-3c/CPCM as the most accurate level of theory (refer to Section IV of the Supporting Information for details). Table 2 shows the corresponding experimental and computed potentials.

**Table 2:** Redox Potentials of 1^**H**^ in THF and MeCN Referenced vs. Fc^+/0^ with Computational Potentials at the PW6B95-D4/def2-QZVPPD/COSMO-RS // B3LYP-3c/CPCM Level of Theory Given in Parentheses

solvent	Mn^I/0^ (V)	Mn^0/‑I^ (V)^a^
MeCN	–1.96 (−1.93)	–2.90
THF	–2.02 (−2.06)	–2.85

a Refer to Part B, Section IV of the SI for a discussion of the computed Mn^0/‑I^ redox potentials.

With this computational methodology in hand, an electrochemical square scheme was constructed by using constrained geometry optimizations at the Mn^I^ and Mn^0^ oxidation states (Scheme 1) to gain additional information on the structural reorganization behavior associated with the Mn^I/0^ redox events. Hence, to replicate the Mn^I/0^ redox potential in the “closed” state ([2^H^_closed_]^−^), the Mn···H distance was fixed at 1.93 Å (based on the computed structure of 1^H^) and the geometry of radical anion [2^H^_closed_]^−^ was optimized. A cathodically shifted redox potential of *E*°_1_ = −2.50 V is calculated, representing a boundary condition with no structural reorganization and substantial electronic repulsion between the metal center and μ-BH moiety. As a result, releasing the constraint via [2^H^_closed_]^−^→ [2^H^]^−^ is strongly exothermic (Δ*G°* = −10.2 kcal/mol). To model the Mn^I/0^ redox potential in the “open” state (1^H^_open_), the computed Mn···H distance 3.12 Å was used as a constraint in 1^H^_open_ for geometry optimization. The redox potential is computed to be *E*°_2_ = −1.53 V, which is anodically shifted due to the absence of B–H stabilization at the formally 16e^–^ Mn center. Expectedly, formation of the μ-BH moiety is strongly exergonic (Δ*G°* = −12.2 kcal/mol). Overall, the midway point between these two boundary cases is −2.01 V when using the constrained model redox data, similar to the computed Mn^I/0^ redox potential in THF from Table 2 (−2.06 V), which further validates the proposed model. Finally, relaxed potential energy surface scan calculations were performed by varying the Mn--H distance for 1^H^_open_ →1^H^ and [2^H^_closed_]^−^→ [2^H^]^−^, respectively, which showed smooth transitions between these geometries (Figure S43). Collectively, these computed observations indicate that electron transfer and structural reorganization are closely coupled, with the B–H–Mn interaction strongly favored at Mn^I^ and strongly disfavored at Mn^0^.

**Scheme 1:**
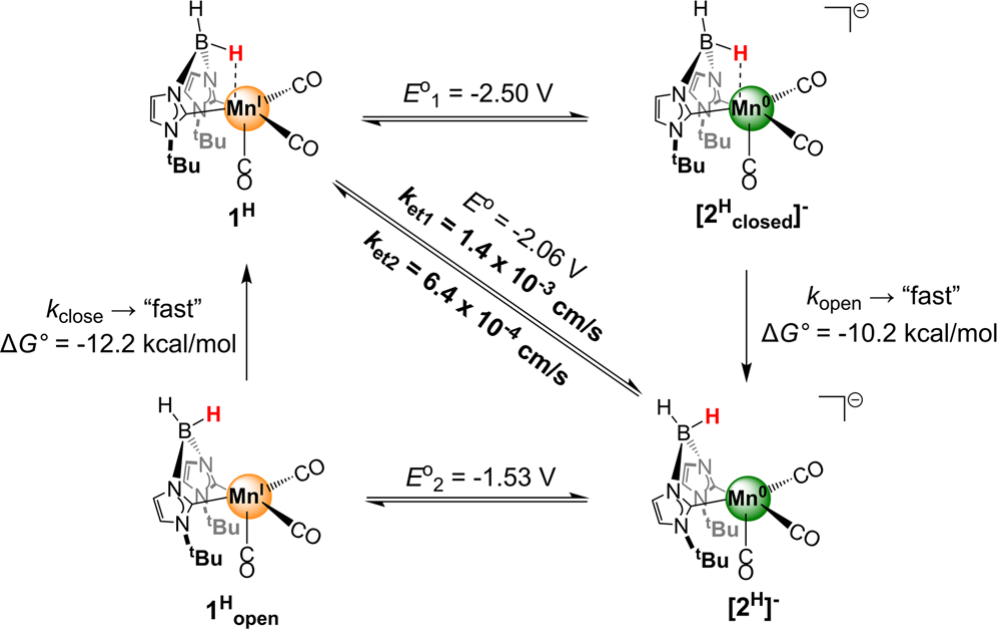
Square Scheme, Showing the Computed Redox Potentials vs Fc^+/0^, the Reaction Free Energies, and the Experimental Electron Transfer Kinetics Data (Bold) in THF

Computations were performed at the PW6B95-D4/def2-QZVPPD/COSMO-RS­(THF)//B3LYP-3c/CPCM­(THF) level of theory

The nearly identical electronic structures of monometallic [2^H^]^−^ and [2^Ph^]^−^ imply that sterics must be playing a critical role in preventing dimerization of the sterically unencumbered [2^H^]^−^ in solution. Therefore, if the bridging borate moiety does not require functionalization to prevent dimerization, why does [2^H^]^−^ remain monometallic? Sterics do appear to be crucial, as early examples of thermally or photochemically generated radicals Mn^0^(CO)_4_L and Mn­(CO)_3_L_2_ slowly decompose or dimerize under ambient anaerobic conditions (L = P^n^Bu_3_, P^i^Bu_3_, P^i^Pr_3_, P­(O^i^Pr)_3_).^49,50,86,87^ In more recently reported five-coordinate Mn^0^ systems, physically blocking the vacant coordination site with bulky isocyanide ligands is also effective,^51,52^ yet there are no guidelines to how much steric bulk is indeed necessary to prepare persistent metalloradicals under ambient conditions.

To help answer the above question using a semiquantitative approach, we turned to calculating percent buried volume (%*V*_bur_), which takes into account steric protection by considering the (scaled by 1.17) van der Waals volume occupied by the coordinating ligands in a 3.5 Å radius surrounding the Mn center.^69,70,88^ We began by comparing crystallographic data sets of monometallic Mn^0^ complexes from the literature and determining their %*V*_bur_ (Figure 5, boxed). We note that only %*V*_bur_ values of individual structures were employed, which thus represent static snapshots of steric bulk without explicit consideration of dynamic effects such as the span of energetically accessible %*V*_bur_ values due to conformational effects.^89^ Nonetheless, the calculated %*V*_bur_ values can still be reliably used to semiquantitatively assess the relative steric pressure exerted by the varying ligand frameworks, which qualitatively correlates well with the general tendency for Mn^0^–Mn^0^ dimerization (vide infra).

**Figure 5:**
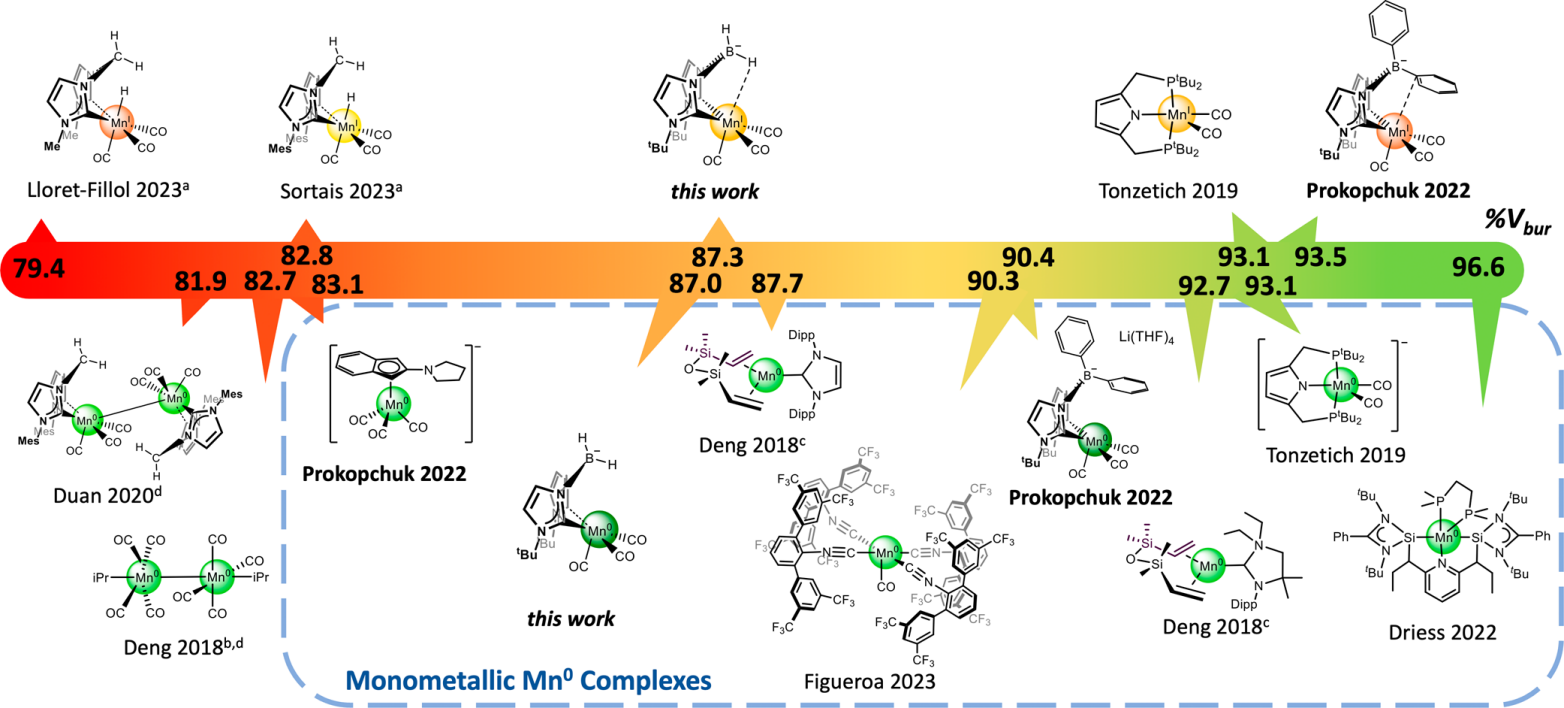
Percent buried volume (*%V*_bur_) analysis of selected crystallographically characterized Mn^I^ (top) and Mn^0^ (bottom) complexes from the literature. ^a^The hydride was omitted from the *%V*_bur_ calculations. ^b^IPr = 1,3-bis (2,6-diisopropylphenyl)­imidazole-2-ylidene. ^c^Dipp = 2,6-diisopropylphenyl. ^d^Only half of the dimer was used with the neighboring Mn atom omitted for *%V*_bur_ calculations.

It is clear that in all these systems the metal center is sterically protected, with a %*V*_bur_ ranging from 83.1% for an indenylmanganese(0) radical anion reported by some of us in 2022 ^53^ to 96.6% for a Mn^0^ bis­(silylene) pincer complex reported by Driess and co-workers in the same year.^57^ Even three-coordinate Mn systems reported by Deng and co-workers show a %*V*_bur_ of 87.7% and 92.7%. However, detailed electronic structure calculations indicate that the formal Mn^0^ oxidation state for these *S* = 3/2 systems is somewhat ambiguous.^47^ Other sterically protected examples include isocyanide-shrouded Mn^0^ complexes by Figueroa and co-workers^51,52^ and a pyrrolide-centered PNP pincer complex by Tonzetich and co-workers.^56^ Moving to %*V*_bur_ values below 83% leads to dimerization, as exemplified by the methylene-bridged Mn­(CO)_3_ dicarbene complex reported by Duan and co-workers (%*V*_bur_ = 81.9%),^48^ which is structurally similar to the systems in this study (Figure 6, bottom left). The NHC-capped Mn­(CO)_4_ dimer by Deng also has a large steric profile (%*V*_bur_ = 82.7%), showing the strong driving force for Mn–Mn bond formation by distorting the metal center to adopt a square-based pyramidal geometry (only half of the dimer was used for %*V*_bur_ calculations with the neighboring Mn atom omitted).^47^ Thus, these crystallographic instances lead us to propose an approximate monomer–dimer %*V*_bur_ threshold of 83% to be critical for the formation/dissociation of Mn^0^–Mn^0^ bonds.^90,91^

**Figure 6:**
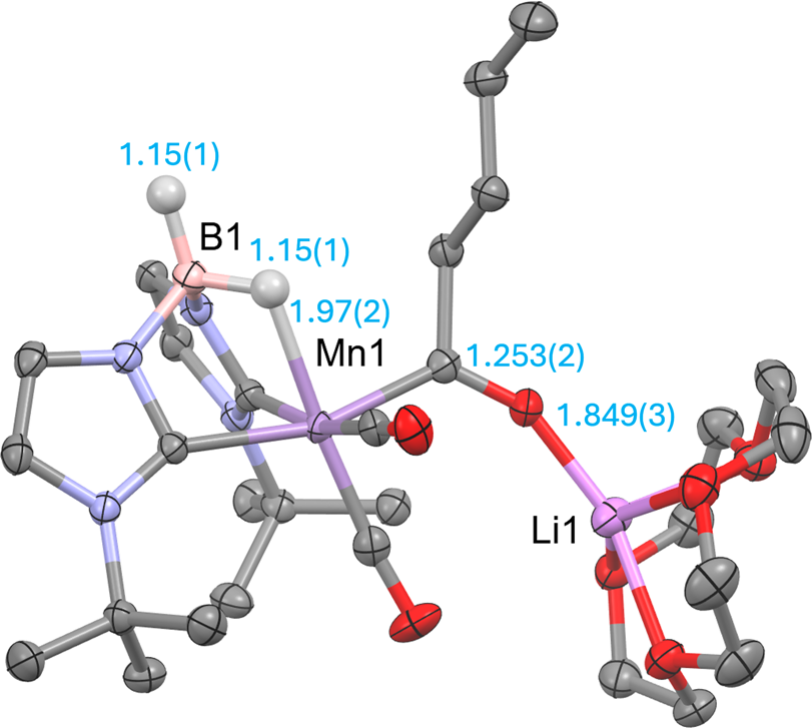
Molecular structure of [Li­(12-crown-4)]­[1^H^-Ac_eq_] with 50% probability ellipsoids. Only one of two molecules in the asymmetric unit are shown and most hydrogen atoms have been removed for clarity. Key bond lengths in Å are shown in blue.

The *%V*_bur_ of these crystallographically characterized Mn^I^ systems can be used as a predictive tool to assess monomer–dimer behavior upon reduction (Figure 5, top). For instance, assessing *%V*_bur_ for the HMn­(CO)_3_(dicarbene) complexes reported by Lloret-Fillol^36^ and Sortais^73^ yields values of 79.4% and 82.8%, with the latter known to dimerize under reducing conditions (hydrides are omitted for these *%V*_bur_ calculations; their inclusion increases *%V*_bur_ by ca. 2.5%). Furthermore, sterically protected Mn^I^ systems, such as 1^H^(*%V*_bur_ = 87.3%), are well within the *%V*_bur_ range to predict formation of a stable Mn^0^ metalloradical under reducing conditions. Nevertheless, while the anionic systems shown in Figure 5 conform well to the purely steric picture based on the calculated *%V*_bur_ values, this analysis does not account for additional contributions such as permanent electrostatic repulsion between like-charged species. Such effects are expected to further disfavor dimerization relative to analogous neutral complexes, irrespective of the steric bulk alone. However, for the examples considered in Figure 5, *%V*_bur_ clearly remains a useful semiquantitative descriptor of the dimerization tendency. A more rigorously quantitative treatment lies beyond the scope of the present study. Lastly, all the molecular examples in Figure 5 contain multiple π-acidic ligands, which appear to be critical for modulating the electron density of the Mn^0^ state.

With a thorough understanding of the steric and electrochemical factors differentiating 1^H^ and 1^Ph^, we sought to investigate the reactivity of these redox–robust complexes with small molecules. We were inspired by the work of Imoto^92^ and Grutzmacher^93^ who reported that redox-active Fe heterocubanes can facilitate the 1e^–^ oxidation and subsequent C–C homocoupling of *n*-butyllithium (*n*-BuLi) and allylmagnesium bromide ((C_3_H_5_)­MgBr) to octane and 1,5-hexadiene, respectively. Thus, we chose these as model substrates to react with 1^Ph^ and 1^H^ (Scheme 2). We first examined the reactivity of 1^Ph^ with 3 equiv of either allylMgBr or *n*-BuLi at room temperature, which generated the homocoupling products 1,5-hexadiene and octane in 48% and 11% yields, respectively (Scheme 2, top). Conducting reactions at temperatures as low as −78 °C did not improve product yields. Both solutions immediately turn from orange to green upon allylMgBr or *n*-BuLi addition due to the formation of the metalloradical [2^Ph^]^−^, which was quantified by UV–vis spectroscopy. Only moderate yields of [2^Ph^]^−^ were produced in the presence of 3 equiv allylMgBr (34%), while nearly quantitative yields were produced in the presence of 1 or 3 equiv *n*-BuLi (96%). Importantly, excess *n*-BuLi does not lead to the doubly reduced complex [3^Ph^]^2–^, showcasing the remarkably high stability of [2^Ph^]^−^, even in the presence of an incredibly basic alkyllithium reagent. Additional side products, including butane (not quantified) and ethylene (ca. 10%), were detected by GC–MS and GC, respectively (Figures S37–S39), also mirroring byproducts observed by Imoto and co-workers.^92^

**Scheme 2:**
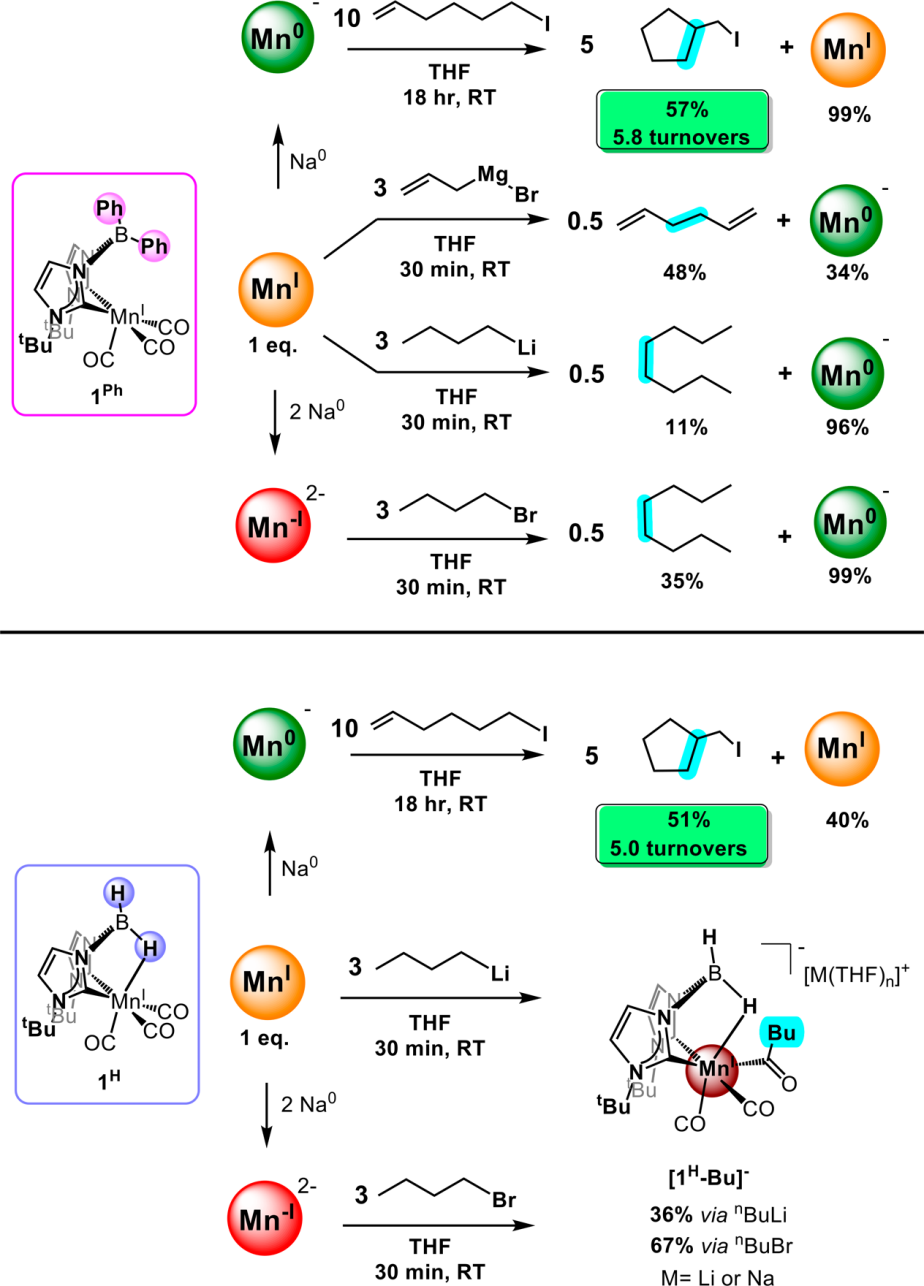
Reactivity of 1^Ph^ and 1^H^ with Nucleophilic and Electrophilic Alkyl and Allyl Reagents

We also sought to test the reactivity of the reduced forms of 1^Ph^. Inspired by the recent work of Lichtenberg on radical cycloisomerization catalysis facilitated by Mn–Bi complexes,^41^ [2^Ph^]^−^ (10 mol %) was reacted with 6-iodo-1-hexene in THF-*d*_8_ to form iodomethylcyclopentane. Using ^1^H NMR spectroscopy for product quantification revealed a 57% yield of the cyclized product (5.8 turnovers) after 18 h with >99% recovery of 1^Ph^ (Scheme 2, top). Most notably, the reaction is carried out at room temperature, starkly contrasting with the thermal conditions required by Lichtenberg et al. (60 °C).^41,42^ We presume that such uniquely mild conditions are possible due to the preformation of the reactive Mn^0^ radical in situ before addition of the alkene to the reaction vessel. Performing that same cyclization reaction at 60 °C yields no product and 81% recovery of 1^Ph^, suggesting that an unknown degradation pathway is operative under thermally forcing conditions. Additionally, the dianion [3^Ph^]^2–^ reacts with the electrophile 1-bromobutane, generating higher yields of octane (35%; see above) and near quantitative yields of [2^Ph^]^−^ (99%; Scheme 2, middle). Collectively, these results indicate facile 1e^–^ transfer, facilitating C–C homocoupling and cycloiosomerization catalysis under ambient conditions.

We then turned our attention to performing analogous reactions with 1^H^ and its reduced forms (Scheme 2, bottom). Reacting 1^H^ with *n*-BuLi or allylMgBr only yielded trace amounts of octane or 1,5-hexadiene, respectively. Instead, a red-brown solution formed after the addition of *n*-BuLi, and crystals suitable for X-ray diffraction were grown in the presence of 12-crown-4, revealing the formation of the diamagnetic acylmanganate­(I) salt [Li­(12-crown-4)]­[1^H^-Ac_eq_] (Figure 6). The acyl group is in the same plane as the dicarbene ligand (defined as the equatorial plane), with the [Li­(12-crown-4)]^+^ ion stabilized via donation from the acyl oxygen atom (Li···O = 1.849(3) Å). While dozens of neutral acylmanganese­(I) complexes have been structurally authenticated, acylmanganate­(I) salts are incredibly rare.^46,94–96^ Formation of the acyl anion [1^H^-Ac_eq_]^−^ demonstrates the divergent reactivity of sterically unencumbered 1^H^, dictated almost solely by sterics at the corresponding borate bridge.

Complex [Li­(THF)_2_]­[1^H^-Ac_eq_] can be synthesized and isolated as a solid from the reaction of 1^H^ with *n*-BuLi in 36% yield or via [3^H^]^2–^ and 1-bromobutane in 67% yield (Scheme 2, bottom). Two equivalents of THF were quantified in the final product by redissolving a pure sample of the orange solid in THF-*d*_8_ and integrating the number of nondeuterated, dissociated THF molecules present in solution, suggesting that solvated [Li­(THF)_n_]^+^ is not strongly ion-paired to [1^H^-Ac_eq_]^−^ in solution. These solutions were further analyzed by ^1^H NMR spectroscopy, clearly revealing four butyl resonances from 2.44 to 0.75 ppm in THF-*d*_8_ (Figure S19). Two-dimensional NMR spectra further indicate that the most deshielded resonance corresponds to the methylene group adjacent to the ketone. As previously observed for 1^H^, [1^H^-Ac_eq_]^−^ also retains its μ–BH interaction, with the hydrogen atom appearing as a sharp doublet at −4.23 ppm (^2^ *J*_HH_ = 10.6 Hz) and the nonbridging hydrogen atom at 3.02 ppm (^2^ *J*_HH_ = 10.3 Hz) in ^1^H­{^11^B} NMR spectra (THF-*d*_8_; Figure S19). Unfortunately, significant line broadening in the ^11^B-coupled ^1^H NMR spectra did not permit an extraction of ^1^ *J*_HB_ coupling data.

The only reaction that proceeded with similar reactivity to 1^Ph^ was the radical cycloisomerization of 6-iodo-1-hexene via [2^H^]^−^, producing a 51% yield of iodomethylcyclopentane after 18 h (5.0 turnovers; Scheme 2, middle). However, only 41% of 1^H^ is recovered. About 20 min into the cyclization reaction, evidence of 1^H^ and an acylated 1-hexene variant of [1^H^-Ac_eq_]^−^ is present in ca. 31% yield based on NMR spectra, with ^1^H and ^11^B peaks closely matching the aforementioned [1^H^-Ac_eq_]^−^ (Figure S28). While mechanistic details for the cycloisomerization reaction remain unclear, this acylated adduct might be a relevant intermediate prior to product formation, as its yield drops to ca. 5% after 18 h. The comparable overall yield of iodomethylcyclopentane relative to cyclization using [2^Ph^]^−^ leads us to speculate that an acylated adduct may also transiently form with [2^Ph^]^−^.

While solid-state data showed that [1^H^-Ac_eq_]^−^ lacks mirror plane symmetry, ^1^H NMR spectra at room temperature show a symmetrical system where the protons on the NHC backbone only show two resonances at 6.74 and 6.59 ppm despite having different trans-positioned ligands. Moreover, ^13^C NMR spectra show only one carbene resonance at 200.2 ppm, further suggesting mirror plane symmetry in solution. Foundational literature on (CO)_n_M*n*-alkyl insertion/elimination chemistry^28,97,98^ supports that butyl elimination from [1^H^-Ac_eq_]^−^ to the site occupied by the μ–BH interaction should be possible, followed by reinsertion into the neighboring cis carbonyl ligand (Figure 7A, boxed). If such a “back and forth” insertion/elimination chemical exchange process at [1^H^-Ac_eq_]^−^ were fast on the NMR time scale at room temperature, signal averaging would account for the appearance of mirror plane symmetry in NMR spectra. Thus, VT ^1^H NMR ranging from −80 to 20 °C was performed in THF-*d*_8_ on a sample of [1^H^-Bu]^−^ with spectra taken in 10 °C increments that reveal a systematic splitting of the two NHC backbone protons into two sets of spin systems at low temperatures (Figures 7B and S20; exchanging proton sites colored in red and purple). The remaining spectral resonances broaden at lower temperatures but remain relatively unchanged. A reduction in symmetry is also observed in the ^13^C NMR spectrum collected at −80 °C, revealing two distinct NHC peaks (200.3, 199.9 ppm) and two CO peaks of equal intensity (242.5, 231.0 ppm; Figures S22 and S23). This observation further supports that the butyl insertion/elimination chemical exchange process has slowed sufficiently at this temperature, allowing the clear observation of [1^H^-Ac_eq_]^−^ in solution. ^1^H NMR spectral line shape simulations for the NHC resonances were performed with the experimental data between −80 °C and −40 °C to provide exchange rate constants (*k*, s^–1^) at each temperature. Eyring analysis with these data points enables the extraction of the transition state (TS) free energy at room temperature (Δ*G* ^‡^ = 13.1 kcal/mol, 298 K; *k* = 1529 s^–1^) along with the TS enthalpies and entropies (Δ*H* ^‡^ = 2.29 kcal/mol and Δ*S* ^‡^ = −36.2 cal/(mol•K), respectively; Figure 7, bottom). Comparison to the DFT-computed elimination TS (Δ*G* ^‡^_deins,ax_ = 12.6 kcal/mol, Figure 7A, top) shows excellent agreement with experiment to make the transient axial adduct [1^H^-Bu_ax_]^−^, further confirming this phenomenon. Once [1^H^-Bu_ax_]^−^ is generated, the barrier to reinsertion is computed to be incredibly low (Δ*G* ^‡^_ins,eq_ = 2.9 kcal/mol), thus rapidly regenerating [1^H^-Ac_eq_]^−^ and reforming the μ–BH interaction with Mn.

**Figure 7:**
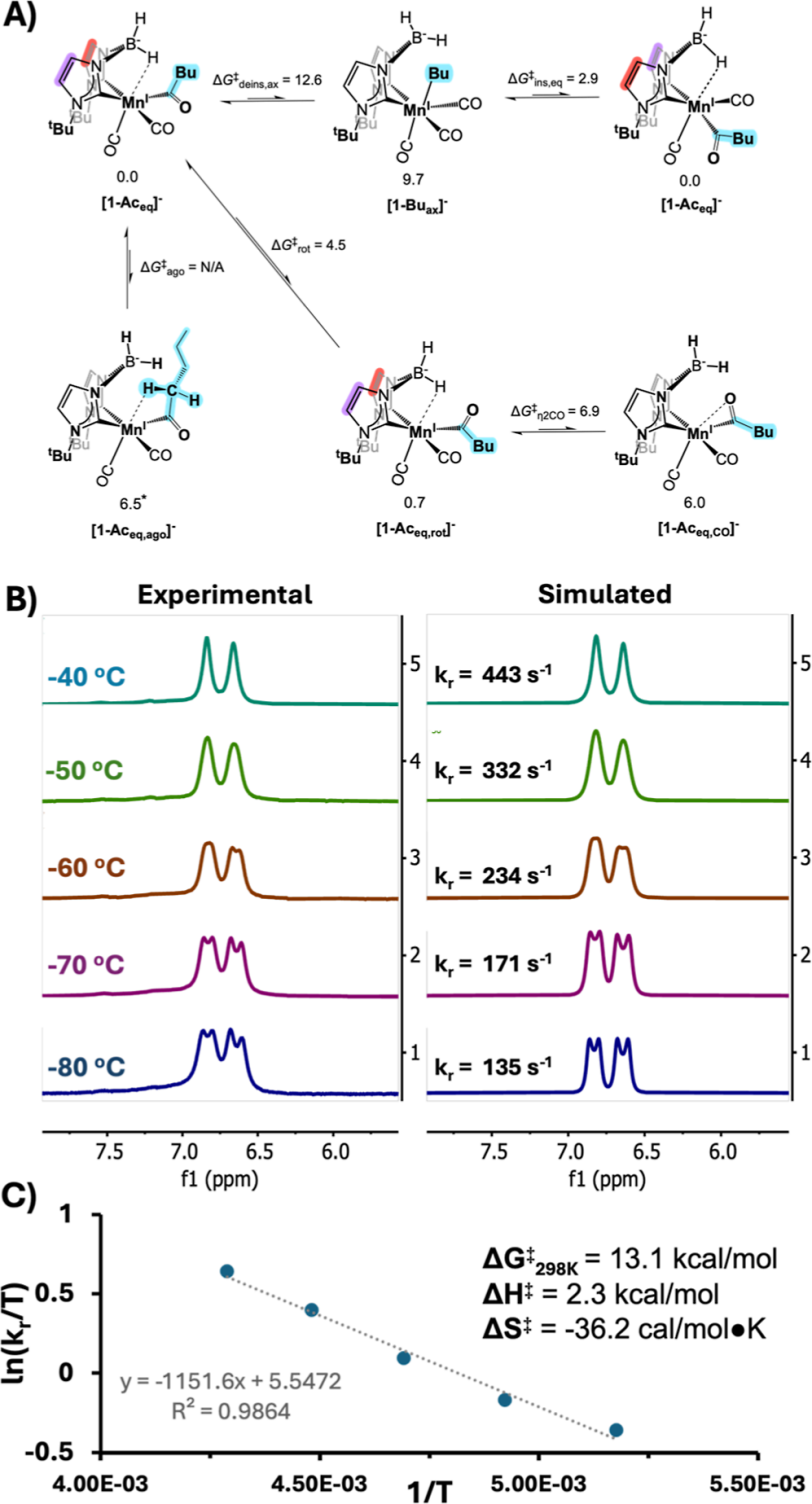
– A: Proposed acyl exchange mechanism as computed at the PW6B95-D4/def2-QZVPPD/COSMO-RS­(THF)//B3LYP-3c/CPCM­(THF) level of theory relative to [1^H^-Ac_eq_]^−^. The NHC backbone resonances that undergo chemical exchange via ^1^H NMR in Figure 7B are colored in purple and red for clarity. All free energies are shown in kcal/mol. The asterisk (*) denotes that [1^H^-Ac_eq,ago_]^−^ could only be obtained in a constrained optimization, using a Mn–H (agostic) distance of 2.22 Å as obtained from a separate calculation at PBE0-D4/def2-TZVPD/CPCM­(THF) level of theory, which is why we were unable to obtain a transition state structure for formation of the agostic interaction (denoted as “N/A”). B: Experimental and simulated ^1^H NMR spectral resonances of the NHC backbone for [1^H^-Bu]^−^ taken between −80 °C and −40 °C along with corresponding exchange rate constants (exchanging protons shown in purple in part A). C: Eyring plot and transition state thermochemistry for the intramolecular butyl exchange using kinetics data from Figure 7B.

One interesting aspect of this intramolecular acyl transfer process is that the μ–BH interaction acts as a “protecting group” to shield the adjacent coordination site at Mn^I^. This adjacent site is typically occupied by solvent (THF, DMSO), β-agostic interactions involving the acyl ligand, or by acyl rotation to form an η^2^-C,O-bound acyl product.^97,99^ The μ–BH interaction thus provides an alternate, intramolecular stabilization pathway specific to the Mn^I^ oxidation state, demonstrating how the BH_2_-bridged dicarbene ligand is uniquely suited to facilitate this novel intramolecular acyl exchange process. Our DFT calculations (see Section VI. of the Supporting Information) further support these claims, as the ground state energy of the β-agostic adduct [1^H^-Ac_eq,ago_]^−^ and the η^2^-C,O-bound acyl adduct [1^H^-Ac_eq,CO_]^−^ are considerably uphill by 6.5 and 6.0 kcal/mol, respectively (Figure 7A). Additionally, THF binding, which is necessarily accompanied by a disruption of the μ–BH interaction, is strongly disfavored given that we were unable to obtain any stable minimum structure with butyl inserted equatorially while still retaining a coordinating THF unit (see Section VII.I. of the Supporting Information). Notably, the observed intramolecular exchange is thermally tolerant, with [1^H^-Bu]^−^ resisting decomposition upon heating at 55 °C for several days. The thermal stability could be partly caused by its overall anionic charge, resulting in lower CO stretching frequencies (i.e., more tightly bound CO ligands; see above) when compared with other neutral acylmanganese­(I) adducts.^20^

## Conclusion

Three novel (^tBu^NHC)_2_BH_2_ bridged Mn complexes in the +1 (1^H^), 0 ([2^H^]^−^), and −1 ([3^H^]^2–^) oxidation states have been synthesized and thoroughly characterized. These complexes exhibit comparable electronic properties to the sterically bulkier phenyl-bridged Mn analogues (1^Ph^, [2^Ph^]^−^, [3^Ph^]^2–^) as evidenced by their redox couples and chemical reduction behavior. Notably, [3^H^]^2–^ is similarly isolable and incredibly reducing with a redox potential of −2.85 V vs Fc^+/0^ in THF. The molecular structure of [2^H^]^−^ reveals a rare mononuclear Mn^0^ metalloradical, with the 1^H^/[2^H^]^−^ redox couple experiencing slower electron transfer kinetics attributed to structural reorganization at Mn, suggesting a stronger μ-BH---Mn interaction compared to the previously observed ipso-C_arene_---Mn interaction in 1^Ph^. While it was initially surprising that the sterically unencumbered [2^H^]^−^ does not form a Mn–Mn bond, percent buried volume (%*V*_bur_) analysis serves as a simple tool to determine that a minimum steric threshold of approximately 83% is needed to avoid dimerization. This finding is also buttressed by analyzing a diverse array of structural Mn^0^ motifs reported in the literature.

Interestingly, the isoelectronic BH_2_- and BPh_2_-bridged Mn complexes display divergent reactivity in the presence of alkyl electrophiles and nucleophiles, yielding either C–C or Mn–C bond formation products depending on the sterics of the substituents at boron. In both cases, the isolable Mn^0^ anions facilitate the radical cycloisomerization catalysis of 6-iodo-1-hexene under ambient conditions–a reaction that commonly requires heating or photochemical activation. The BH_2_-bridged complex generates a unique Mn acyl adduct [1^H^-Bu]^−^ via carbonyl ligand activation in the presence of *n*-BuLi or 1-bromobutane, with NMR spectroscopy, single crystal X-ray diffraction, and DFT mechanistic studies supporting that [1^H^-Bu]^−^ retains its μ–BH interaction and undergoes rapid equatorial acyl exchange in solution. These data also indicate that the μ–BH interaction acts as a “protecting group” to stabilize the molecule, but the interaction is still weak enough to allow for rapid acyl elimination/insertion chemistry to occur. Overall, the fascinating redox chemistry and small molecule activation exhibited by these borate-bridged Mn dicarbene systems provide key insights into the role of ligand sterics in C–C bond formation and catalysis, suggesting that new modes of reactivity can be unlocked by fine-tuning the substitution chemistry at boron. Ongoing efforts are aimed at expanding the breadth of small molecule activation catalysis with electrophiles (i.e., CO_2_) using this unique family of redox-active Mn complexes.

## Supplementary Material





## Notes

Syntheses, Spectra, Additional Figures and Schemes, Additional Computational Details (PDF) (PDF)
Computed Structures and Additional Data (ZIP) (ZIP)

## References

[1] Ellis J. E., Faltynek R. A. (1975). The tetracarbonyl trianions of manganese and rhenium,
M­(CO)_4_
^3–^. J. Chem.
Soc., Chem. Commun..

[2] Wieberneit F., Korber N. (2025). Carbonyl Metalates
in Liquid Ammonia: Reduction of
Mn_2_(CO)_10_ down to [Mn­(CO)_4_]^3–^. Eur. J. Inorg. Chem..

[3] Cassebaum H. (1979). History of
the “mineral chameleon” (permanganate). Die Pharmazie.

[4] Fleischer, E. Managate Des Baryts; Chemisches Zentralblatt, 1873; Vol. 44, pp 737–739.

[5] Sahu S., Goldberg D. P. (2016). Activation of Dioxygen
by Iron and Manganese Complexes:
A Heme and Nonheme Perspective. J. Am. Chem.
Soc..

[6] Zaragoza J. P. T., Siegler M. A., Goldberg D. P. (2018). A Reactive Manganese­(IV)–Hydroxide
Complex: A Missing Intermediate in Hydrogen Atom Transfer by High-Valent
Metal-Oxo Porphyrinoid Compounds. J. Am. Chem.
Soc..

[7] Saju A., Griffiths J. R., MacMillan S. N., Lacy D. C. (2022). Synthesis of a Bench-Stable
Manganese­(III) Chloride Compound: Coordination Chemistry and Alkene
Dichlorination. J. Am. Chem. Soc..

[8] Saju A., Crawley M. R., MacMillan S. N., Lacy D. C. (2024). Manganese­(III) Nitrate
Complexes as Bench-Stable Powerful Oxidants. J. Am. Chem. Soc..

[9] Ban H. T., Kase T., Murata M. (2001). Manganese-based transition metal
complexes as new catalysts for olefin polymerizations. J. Polym. Sci., Part A: Polym. Chem..

[10] Ihara E., Todaka T., Inoue K. (2002). Polymerization of Methyl Methacrylate
Initiated with Organomanganate Reagents. Macromol.
Rapid Commun..

[11] Layfield R. A. (2008). Manganese­(ii):
the black sheep of the organometallic family. Chem. Soc. Rev..

[12] Sood A., Räisänen M. T., Aitola E., Sibaouih A., Colacio E., Ahlgren M., Nieger M., Repo T., Leskelä M. (2013). Synthesis
and crystal structure determination of Mn­(II)
Schiff base complexes and their performance in ethene polymerization. Polyhedron.

[13] Ottenbacher R. V., Samsonenko D. G., Talsi E. P., Bryliakov K. P. (2012). Highly
Efficient, Regioselective, and Stereospecific Oxidation of Aliphatic
C–H Groups with H_2_O_2_, Catalyzed by Aminopyridine
Manganese Complexes. Org. Lett..

[14] Shen D., Miao C., Wang S., Xia C., Sun W. (2014). Efficient
Benzylic and Aliphatic C–H Oxidation with Selectivity for Methylenic
Sites Catalyzed by a Bioinspired Manganese Complex. Org. Lett..

[15] Ottenbacher R. V., Talsi E. P., Bryliakov K. P. (2015). Mechanism of Selective C–H
Hydroxylation Mediated by Manganese Aminopyridine Enzyme Models. ACS Catal..

[16] Galeotti M., Bietti M., Costas M. (2024). Catalyst and
Medium Control over
Rebound Pathways in Manganese-Catalyzed Methylenic C–H Bond
Oxidation. J. Am. Chem. Soc..

[17] Ahn C., Gomez A., Hartmann M. A., White M. C. (2025). Selective Methylene
Oxidation in α,β-Unsaturated Carbonyl Natural Products. Nature.

[18] Coffield T., Kozikowski J., Closson R. (1957). Communications - Acyl Manganese Pentacarbonyl
Compounds. J. Org. Chem..

[19] Noack K., Calderazzo F. (1967). Carbon monoxide insertion reactions V. The carbonylation
of methylmanganese pentacarbonyl with ^13^CO. J. Organomet. Chem..

[20] Andersen J.-A. M., Moss J. R. (1994). Synthesis of an Extensive Series
of Manganese Pentacarbonyl
Alkyl and Acyl Compounds: Carbonylation and Decarbonylation Studies
on [Mn­(R)­(CO)_5_] and [Mn­(COR)­(CO)_5_]. Organometallics.

[21] Elangovan S., Neumann J., Sortais J.-B., Junge K., Darcel C., Beller M. (2016). Efficient and selective N-alkylation of amines with
alcohols catalysed by manganese pincer complexes. Nat. Commun..

[22] Kallmeier F., Irrgang T., Dietel T., Kempe R. (2016). Highly Active
and Selective
Manganese C = O Bond Hydrogenation Catalysts: The Importance of the
Multidentate Ligand, the Ancillary Ligands, and the Oxidation State. Angew. Chem., Int. Ed..

[23] Mukherjee A., Nerush A., Leitus G., Shimon L. J. W., Ben
David Y., Espinosa Jalapa N. A., Milstein D. (2016). Manganese-Catalyzed
Environmentally Benign Dehydrogenative Coupling of Alcohols and Amines
to Form Aldimines and H_2_: A Catalytic and Mechanistic Study. J. Am. Chem. Soc..

[24] Alig L., Fritz M., Schneider S. (2019). First-Row Transition Metal (De)­Hydrogenation
Catalysis Based On Functional Pincer Ligands. Chem. Rev..

[25] Daw P., Kumar A., Espinosa-Jalapa N. A., Ben-David Y., Milstein D. (2019). Direct Synthesis of
Amides by Acceptorless Dehydrogenative
Coupling of Benzyl Alcohols and Ammonia Catalyzed by a Manganese Pincer
Complex: Unexpected Crucial Role of Base. J.
Am. Chem. Soc..

[26] Freitag F., Irrgang T., Kempe R. (2019). Mechanistic Studies of Hydride Transfer
to Imines from a Highly Active and Chemoselective Manganate Catalyst. J. Am. Chem. Soc..

[27] Weber, S. ; Kirchner, K. The Role of Metal-Ligand Cooperation in Manganese­(I)-Catalyzed Hydrogenation/Dehydrogenation Reactions; Springer: Berlin Heidelberg, 2020; pp 1–35.

[28] Weber S., Kirchner K. (2022). Manganese Alkyl Carbonyl Complexes: From Iconic Stoichiometric
Textbook Reactions to Catalytic Applications. Acc. Chem. Res..

[29] Sila N., Schwarz T., Dickert A., Irrgang T., Kempe R. (2025). The Special
Thing about Mn (de)­hydrogenation Catalysts. Organometallics.

[30] Bourrez M., Molton F., Chardon-Noblat S., Deronzier A. (2011). [Mn­(bipyridyl)­(CO)_3_Br]: An Abundant Metal
Carbonyl Complex as Efficient Electrocatalyst
for CO_2_ Reduction. Angew. Chem.,
Int. Ed..

[31] Smieja J. M., Sampson M. D., Grice K. A., Benson E. E., Froehlich J. D., Kubiak C. P. (2013). Manganese as a Substitute for Rhenium in CO_2_ Reduction Catalysts: The Importance of Acids. Inorg. Chem..

[32] Sampson M.
D., Nguyen A. D., Grice K. A., Moore C. E., Rheingold A. L., Kubiak C. P. (2014). Manganese Catalysts with Bulky Bipyridine Ligands for
the Electrocatalytic Reduction of Carbon Dioxide: Eliminating Dimerization
and Altering Catalysis. J. Am. Chem. Soc..

[33] Sampson M. D., Kubiak C. P. (2016). Manganese Electrocatalysts
with Bulky Bipyridine Ligands:
Utilizing Lewis Acids To Promote Carbon Dioxide Reduction at Low Overpotentials. J. Am. Chem. Soc..

[34] Franco F., Pinto M. F., Royo B., Lloret-Fillol J. (2018). A Highly Active
N-Heterocyclic Carbene Manganese­(I) Complex for Selective Electrocatalytic
CO_2_ Reduction to CO. Angew. Chem.,
Int. Ed..

[35] Barrett J. A., Miller C. J., Kubiak C. P. (2021). Electrochemical Reduction of CO_2_ Using Group VII Metal Catalysts. Trends
Chem..

[36] Fernández S., Franco F., Martínez
Belmonte M., Friães S., Royo B., Luis J. M., Lloret-Fillol J. (2023). Decoding the
CO_2_ Reduction Mechanism of a Highly Active Organometallic
Manganese Electrocatalyst: Direct Observation of a Hydride Intermediate
and Its Implications. ACS Catal..

[37] Koizumi H., Tamaki Y., Kamogawa K., Nicaso M., Suzuki Y., Yamazaki Y., Takeda H., Ishitani O. (2025). Development of a Highly
Durable Photocatalytic CO2 Reduction Using a Mn-Complex Catalyst:
Application of Selective Photosplitting of a Mn(0)–Mn(0) Bond. J. Am. Chem. Soc..

[38] Dey S., Masero F., Brack E., Fontecave M., Mougel V. (2022). Electrocatalytic metal hydride generation using CPET
mediators. Nature.

[39] Sharma R., P S., Shenoy P. S., Gautam N., Gope B., Maji S., Pati S. K., Mandal S. K. (2025). Photoexcited
Manganese Complex of
Abnormal N-Heterocyclic Carbene in Molecular Hydrogen Activation via
Hydrogen Atom Transfer Pathway. ACS Catal..

[40] Li Y., Zhu F., Wang Z., Rabeah J., Brückner A., Wu X.-F. (2017). Practical and General Manganese-Catalyzed Carbonylative Coupling
of Alkyl Iodides with Amides. ChemCatChem.

[41] Ramler J., Krummenacher I., Lichtenberg C. (2019). Bismuth Compounds in Radical Catalysis:
Transition Metal Bismuthanes Facilitate Thermally Induced Cycloisomerizations. Angew. Chem., Int. Ed..

[42] Martínez S., Junghanns M. A., Dunaj T., Lichtenberg C. (2025). Bismuth Radical
Catalysis: Thermally Induced Intramolecular C­(sp^3^)–C­(sp)
Cyclization of Unactivated Alkyl Iodides and Alkynes. ACS Catal..

[43] Bao Z.-P., Wu X.-F. (2025). Manganese- and Iron-Catalyzed Carbonylation
Reactions: A Personal
Account. Synlett.

[44] Liu P.-R., Ji M.-M., Hu J.-B., Peng J.-B. (2024). Manganese-Catalyzed
Carbonylation of Unactivated Alkyl Bromides with Alkylidenecyclopropanes. ACS Catal..

[45] Hartl F., Mahabiersing T., Le Floch P., Mathey F., Ricard L., Rosa P., Záliš S. (2003). Electronic
Properties of 4,4̀,5,5̀-Tetramethyl-2,2̀-biphosphinine
(tmbp) in the Redox Series fac-[Mn­(Br)­(CO)_3_(tmbp)], [Mn­(CO)_3_(tmbp)]_2_, and [Mn­(CO)_3_(tmbp)]-: Crystallographic,
Spectroelectrochemical, and DFT Computational Study. Inorg. Chem..

[46] Shao L., Geib S. J., Badger P. D., Cooper N. J. (2003). Reductive Dimerization
of the Cyclohexadienyl Complex [Mn­(CO)_3_(η^5^-C_6_H_7_)] through a Radical Pathway. Organometallics.

[47] Cheng J., Chen Q., Leng X., Ouyang Z., Wang Z., Ye S., Deng L. (2018). The Stabilization of Three-Coordinate Formal Mn(0)
Complex with NHC and Alkene Ligation. Chem.

[48] Yang Y., Zhang Z., Chang X., Zhang Y.-Q., Liao R.-Z., Duan L. (2020). Highly Active Manganese-Based
CO_2_ Reduction Catalysts
with Bulky NHC Ligands: A Mechanistic Study. Inorg. Chem..

[49] Kidd D. R., Cheng C. P., Brown T. L. (1978). Formation and properties of substituted
manganese carbonyl radicals. J. Am. Chem. Soc..

[50] McCullen S. B., Brown T. L. (1982). Preparation, properties,
and kinetics of the reaction
of manganese(0) radicals Mn­(CO)_3_(R_3_P)_2_ with tributyltin hydride. J. Am. Chem. Soc..

[51] Agnew D. W., Moore C. E., Rheingold A. L., Figueroa J. S. (2015). Kinetic Destabilization
of Metal-Metal Single Bonds: Isolation of a Pentacoordinate Manganese(0)
Monoradical. Angew. Chem., Int. Ed..

[52] Salsi F., Wang S., Teutloff C., Busse M., Neville M. L., Hagenbach A., Bittl R., Figueroa J. S., Abram U. (2023). A Complete
Triad of Zero-Valent 17-Electron Monoradicals of Group 7 Elements
Stabilized by m-Terphenyl Isocyanides. Angew.
Chem., Int. Ed..

[53] Tresp D. S., Neugebauer H., Grimme S., Hansen A., Prokopchuk D. E. (2022). Electronic
Effects of Aminoindenyl Ligands Coordinated to Manganese: Structures
and Properties of a Mn^0^ Metalloradical and Bimetallic Mn^–I^/Mn^I^ Adduct. Organometallics.

[54] Karagiannis A., Tyryshkin A. M., Lalancette R. A., Spasyuk D. M., Washington A., Prokopchuk D. E. (2022). A redox-active Mn(0) dicarbene metalloradical. Chem. Commun..

[55] Karagiannis A., Neugebauer H., Lalancette R. A., Grimme S., Hansen A., Prokopchuk D. E. (2024). Pushing the Limits of Organometallic Redox Chemistry
with an Isolable Mn­(−I) Dianion. J. Am.
Chem. Soc..

[56] Narro A. L., Arman H. D., Tonzetich Z. J. (2019). Manganese Chemistry of Anionic Pyrrole-Based
Pincer Ligands. Organometallics.

[57] Kalra S., Pividori D., Fehn D., Dai C., Dong S., Yao S., Zhu J., Meyer K., Driess M. (2022). A bis­(silylene)­pyridine
pincer ligand can stabilize mononuclear manganese(0) complexes: facile
access to isolable analogues of the elusive d^7^-Mn­(CO)_5_ radical. Chem. Sci..

[58] Leong V. S., Cooper N. J. (1988). Synthesis and chemical characterization of [Mn­(η^5^-C_5_H_5_)­(CO)_2_]^2‑^. Organometallics.

[59] Lee S., Cooper N. J. (1991). Reductive activation of the η^5^-methylcyclopentadienyl
ligand in [Mn­(η^5^-C_5_H_4_Me)­(CO)_3_] and evidence for a ring-slipped intermediate containing
an η^3^—methylcyclopentadienyl ligand. J. Am. Chem. Soc..

[60] Anson C. W., Stahl S. S. (2020). Mediated Fuel Cells: Soluble Redox Mediators and Their
Applications to Electrochemical Reduction of O_2_ and Oxidation
of H_2_, Alcohols, Biomass, and Complex Fuels. Chem. Rev..

[61] Reid A. G., Machan C. W. (2023). Redox Mediators in Homogeneous Co-electrocatalysis. J. Am. Chem. Soc..

[62] Soloveichik G. L. (2015). Flow Batteries:
Current Status and Trends. Chem. Rev..

[63] Winsberg J., Hagemann T., Janoschka T., Hager M. D., Schubert U. S. (2017). Redox-Flow
Batteries: From Metals to Organic Redox-Active Materials. Angew. Chem., Int. Ed..

[64] Gandeepan P., Finger L. H., Meyer T. H., Ackermann L. (2020). 3d metallaelectrocatalysis
for resource economical syntheses. Chem. Soc.
Rev..

[65] Kim H., Kim H., Lambert T. H., Lin S. (2020). Reductive Electrophotocatalysis:
Merging Electricity and Light To Achieve Extreme Reduction Potentials. J. Am. Chem. Soc..

[66] Cardinale, L. ; Stahl, S. S. ; Kalyani, D. ; Lehnherr, D. Chapter Two - Overview of outer-sphere electron transfer mediators for electrosynthesis. In Adv. Catal.; Hevia, E. , Pérez-Temprano, M. H. , Diéguez, M. , Eds.; Academic Press, 2023; Vol. 72, pp 57–102.

[67] Maxwell V., Karagiannis A., Schramm T., Kotelnikow V., Gupta R., Lalancette R., Appel A., Hansen A., Wiedner E., Prokopchuk D. (2026). Redox Robust Mn­(I/0/-I) Dicarbenes:
Electrochemistry, Steric Mapping, and Borate-Directed C-C Bond Formation. ChemRxiv.

[68] Hillier A. C., Sommer W. J., Yong B. S., Petersen J. L., Cavallo L., Nolan S. P. (2003). A Combined Experimental and Theoretical Study Examining
the Binding of N-Heterocyclic Carbenes (NHC) to the Cp*RuCl (Cp* =
η^5^-C_5_Me_5_) Moiety: Insight into
Stereoelectronic Differences between Unsaturated and Saturated NHC
Ligands. Organometallics.

[69] Poater A., Cosenza B., Correa A., Giudice S., Ragone F., Scarano V., Cavallo L. (2009). SambVca: A Web Application for the
Calculation of the Buried Volume of N-Heterocyclic Carbene Ligands. Eur. J. Inorg. Chem..

[70] Falivene L., Credendino R., Poater A., Petta A., Serra L., Oliva R., Scarano V., Cavallo L. (2016). SambVca 2. A Web Tool
for Analyzing Catalytic Pockets with Topographic Steric Maps. Organometallics.

[71] Fox P., Griffin S., Reichert W., Salter E., Smith A., Tickell M., Wicker B., Cioffi E., Davis Jr J. H., Rogers R. (2005). Exploiting isolobal relationships to create new ionic
liquids: Novel room-temperature ionic liquids based upon (N-alkylimidazole)­(amine)­BH_2_
^+^ “boronium” ions. Chem. Commun..

[72] Forshaw A. P., Bontchev R. P., Smith J. M. (2007). Oxidation
of the Tris­(carbene)­borate
Complex PhB­(MeIm)_3_ Mn^I^ (CO)_3_ to Mn^IV^ [PhB­(MeIm)_3_ ]_2_ (OTf)_2_. Inorg. Chem..

[73] Azouzi K., Pedussaut L., Pointis R., Bonfiglio A., Kumari Riddhi R., Duhayon C., Bastin S., Sortais J.-B. (2023). Hydrogenation
of Carboxylic Esters Catalyzed by Phosphine-Free Bis-N-heterocyclic
Carbene Manganese Complexes. Organometallics.

[74] Saha K., Roy D. K., Dewhurst R. D., Ghosh S., Braunschweig H. (2021). Recent Advances
in the Synthesis and Reactivity of Transition Metal σ-Borane/Borate
Complexes. Acc. Chem. Res..

[75] Brookhart, M. ; Green, M. L. H. ; Wong, L.-L. Carbon-Hydrogen-Transition Metal Bonds. In Prog. Inorg. Chem.; John Wiley & Sons, Ltd, 1988; pp 1–124.

[76] Brookhart M., Green M. L. H., Parkin G. (2007). Agostic interactions
in transition
metal compounds. Proc. Natl. Acad. Sci. U. S.
A..

[77] Ramalakshmi R., Saha K., Roy D. K., Varghese B., Phukan A. K., Ghosh S. (2015). New Routes to a Series
of σ-Borane/Borate Complexes of Molybdenum
and Ruthenium. Chem. Eur J..

[78] Gupta R., Taguchi T., Borovik A. S., Hendrich M. P. (2013). Characterization
of Monomeric MnII/III/IV–Hydroxo Complexes from X- and Q-Band
Dual Mode Electron Paramagnetic Resonance (EPR) Spectroscopy. Inorg. Chem..

[79] Gupta R., Taguchi T., Lassalle-Kaiser B., Bominaar E. L., Yano J., Hendrich M. P., Borovik A. S. (2015). High-spin Mn–oxo complexes
and their relevance to the oxygen-evolving complex within photosystem
II. Proc. Natl. Acad. Sci. U. S. A..

[80] Neese F. (2025). Software Update:
The ORCA Program System-Version 6.0. WIREs Comput.
Mol. Sci..

[81] Zapata
Trujillo J. C., McKemmish L. K. (2022). VIBFREQ1295: A New Database for Vibrational
Frequency Calculations. J. Phys. Chem. A.

[82] Zapata
Trujillo J. C., McKemmish L. K. (2023). Model Chemistry Recommendations for
Scaled Harmonic Frequency Calculations: A Benchmark Study. J. Phys. Chem. A.

[83] Wu K., Laws D. R., Nafady A., Geiger W. E. (2014). Substitution of
CO Ligand by P­(OPh)_3_ in Radical Cations of the Cymantrene
Family: Relationships of Substitution Rates to E_1/2_ Values
and Carbonyl IR Frequencies. J. Inorg. Organomet.
Polym. Mater..

[84] Bard, A. J. ; Faulkner, L. R. Electrochemical Methods: Fundamentals and Applications; John Wiley & Sons, Inc, 2001.

[85] Savéant, J. M. ; Costentin, C. Elements of Molecular and Biomolecular Electrochemistry; John Wiley & Sons, Ltd, 2019; . 10.1002/9781119292364.

[86] Kidd D. R., Brown T. L. (1978). Photochemical substitution reactions of decacarbonyldimanganese. J. Am. Chem. Soc..

[87] Rattinger G. B., Belford R. L., Walker H., Brown T. L. (1989). EPR-derived electronic
characteristics of zerovalent manganese carbonyl di­(tertiary phosphine)
radicals. Inorg. Chem..

[88] Schaefer A. J., Ingman V. M., Wheeler S. E. (2021). SEQCROW: A ChimeraX
bundle to facilitate
quantum chemical applications to complex molecular systems. J. Comput. Chem..

[89] Newman-Stonebraker S. H., Smith S. R., Borowski J. E., Peters E., Gensch T., Johnson H. C., Sigman M. S., Doyle A. G. (2021). Univariate classification
of phosphine ligation state and reactivity in cross-coupling catalysis. Science.

[90] Pracht P., Bohle F., Grimme S. (2020). Automated exploration of the low-energy
chemical space with fast quantum chemical methods. Phys. Chem. Chem. Phys..

[91] Pracht P., Grimme S., Bannwarth C., Bohle F., Ehlert S., Feldmann G., Gorges J., Müller M., Neudecker T., Plett C. (2024). CREST-A
program for
the exploration of low-energy molecular chemical space. J. Chem. Phys..

[92] Inoue H., Fujimoto N., Imoto E. (1977). Electron-transfer
reduction of fluorenone
with n-butyl-lithium mediated by an iron–sulphur cluster. J. Chem. Soc., Chem. Commun..

[93] Lichtenberg C., Garcia Rubio I., Viciu L., Adelhardt M., Meyer K., Jeschke G., Grützmacher H. (2015). A Low-Valent
Iron Imido Heterocubane Cluster: Reversible Electron Transfer and
Catalysis of Selective C–C Couplings. Angew. Chem., Int. Ed..

[94] Casey C. P., Bunnell C. A. (1976). Site of nucleophilic attack on acylpentacarbonylmanganese­(I)
compounds. J. Am. Chem. Soc..

[95] Gladysz J. A., Selover J. C., Strouse C. E. (1978). α-Silyloxy
and α-hydroxy
manganese alkyls. Generation via a new five-membered metallocycle. J. Am. Chem. Soc..

[96] Selover J. C., Vaughn G. D., Strouse C. E., Gladysz J. A. (1986). Synthesis and reactivity
of metal α-hydroxyalkyl complexes; generation of (CO)_5_MCH­(C_6_H_5_)­OH (M = Mn, Re). J. Am. Chem. Soc..

[97] Calderazzo F. (1977). Synthetic
and Mechanistic Aspects of Inorganic Insertion Reactions. Insertion
of Carbon Monoxide. Angew. Chem., Int. Ed. Engl..

[98] Maitlis P. M., Belli Dell’Amico D. (2014). Fausto Calderazzo:
Pioneer in Mechanistic
Organometallic Chemistry. Organometallics.

[99] Zhou T., Malakar S., Webb S. L., Krogh-Jespersen K., Goldman A. S. (2019). Polar molecules catalyze CO insertion
into metal-alkyl
bonds through the displacement of an agostic C-H bond. Proc. Natl. Acad. Sci. U. S. A..

