# Supplementary material for: Steric Mapping, Ligand Dynamics, and Cycloisomerization Catalysis with Redox Robust MnI/0/‑I Dicarbenes
Source: Organometallics. 2026 Apr 27;45(12):1355–67. doi: 10.1021/acs.organomet.6c00057 (PMC13292348; doi:10.1021/acs.organomet.6c00057)
Supplement: Supplementary file 1 [file om6c00057_si_001.pdf]

Supporting Information for:

Steric Mapping, Ligand Dynamics, and Cycloisomerization Catalysis with Redox Robust $Mn^{I/0/-I}$ Dicarbenes

Viani Maxwell[a], Ageliki Karagiannis[a], Tim K. Schramm[b], Veronika Kotelnikow[b], Rupal Gupta[c], Roger A. Lalancette[a], Aaron M. Appel[d], Andreas Hansen[b]*, Eric S. Wiedner[d]*, and Demyan E. Prokopchuk[a]*

[a]Department of Chemistry, Rutgers University – Newark, Newark, NJ, 07102, United States
[b]Mulliken Center for Theoretical Chemistry, Clausius Institute for Physical and Theoretical Chemistry, Rhenish Friedrich Wilhelms University of Bonn, Bonn 53115, Germany
[c]Department of Chemistry and Biochemistry, College of Staten Island, City University of New York, 2800 Victory Blvd., Staten Island, New York, 10314, United States
[d]Institute for Integrated Catalysis, Pacific Northwest National Laboratory, P.O. Box 999, Richland, Washington 99352, United States
*Email: demyan.prokopchuk@rutgers.edu, eric.wiedner@pnnl.gov, hansen@thch.uni-bonn.de

Part A

Experimental

General Comments 4
Syntheses 6
[$H_2B(^{tBu}NHC)_2Mn(CO)_3$] (**$1^H$**) 6
[Na(2.2.2)crypt][**2^H**] 6
[K(2.2.2)crypt][**2^H**] 7
[K(18-c-6(THF)$_x$)][**2^H**] 7
[Na(THF)$_x$]$_2$[**3^H**] 7
Reactions with *n*-BuLi 7
Synthesis of [Li(THF)$_x$][**2^{Ph}**] and octane 7
Synthesis of [Li(THF)$_2$][**1^H-Ac$_{eq}$**] 8
Synthesis and crystallization of [Li(12-c-4)][**1^H-Ac$_{eq}$**] 8
Reactions with allylmagnesium bromide 8
Synthesis of **[2^{Ph}]**$^-$ and 1,5-hexadiene 8
Reactions with 1-bromobutane 9
Synthesis of [Na(THF)$_x$][**2^{Ph}**] and octane 9
Synthesis of [Na(THF)$_x$][**1^H-Ac$_{eq}$**] 9
Reactions with 6-Iodo-1-hexene 9
Synthesis of Iodomethylcyclopentane via [**1^{Ph}**]$^-$ 9
Synthesis of Iodomethylcyclopentane via [**1^H**]$^-$ 10
Electrochemistry 10
NMR Spectra 14
UV-Vis Spectra 31
GC-MS 36

General Comments

All reactions were carried out under an atmosphere of nitrogen (N_2) using a standard glove box or Schlenk techniques unless stated otherwise. All reagents and solvents were stored in an N_2 filled glove box prior to use. Acetonitrile (CH_3CN), dichloromethane (DCM), dimethoxy ethane (DME), diethyl ether (Et_2O), tetrahydrofuran (THF), fluorobenzene, toluene, and pentane were dried and degassed over activated alumina using an IT/Inert solvent purification system. Additionally, MeCN, DCM, Et_2O, fluorobenzene, and toluene were dried over 20% w/v activated 3 Å molecular sieves.[1] For chemical reduction reactions, THF was dried again over 20% w/v activated 3 Å molecular sieves. Glassware was dried overnight at 140°C and cooled under dynamic vacuum in a glove box antechamber. Glass fiber filter discs (1.5 µm) were dried overnight at 140°C and stored in a glove box. For all reduction reactions, surface passivated (i.e., blackened) Teflon-coated stir bars were used that have already been exposed to alkali metal and/or naphthalenide reducing agents. Compounds $[(C_2B^H)_2BH_2]I$,[2,3] $[Mn(CO)_3(NC^{tBu})Br]_2$,[4] and $[Ph_2B(^{tBu}NHC)_2Mn(CO)_3]$[5] (**1^{Ph}**) were prepared according to known procedures. The commercially purchased electrolyte $[^nBu_4N][PF_6]$ (99+%) was recrystallized from fluorobenzene/pentane in a glovebox prior to use. All other reagents were purchased from commercial suppliers and used as received. Allyl magnesium bromide ($Mg(C_3H_5)Br$, 1.0 M in Et_2O), purchased from chemical suppliers ThermoFischer and ChemCruz and was found to contain varying amounts of the C-C coupling byproduct 1,5-hexadiene. Elemental analyses (EA) were obtained from the CENTC Elemental Analysis Facility at the University of Rochester. For EA, microanalysis samples were weighed with a PerkinElmer Model AD6000 Autobalance and their composition determined with a PerkinElmer 2400 Series II Analyzer. After multiple attempts on independently prepared samples of **1^H**, [Na(2.2.2)crypt][**2^H**], and $[Na(THF)_x]$[**1^H-Bu**], EA results on independently prepared batches were consistently low in carbon and/or nitrogen. Using a combustion aid did not affect the outcomes, and we attribute this discrepancy to incomplete combustion due to the presence of the borate anion.[6] Similar discrepancies were observed with **1^{Ph}** and reduction products.[7] Percent buried volume analysis was conducted using SambVca as part of the SEQCROW package in UCSF ChimeraX version 1.9rc202411210252 developed by the Resource for Biocomputing, Visualization, and Informatics at the University of California, San Francisco, with support from National Institutes of Health R01-GM129325 and the Office of Cyber Infrastructure and Computational Biology, National Institute of Allergy and Infectious Diseases (https://www.rbvi.ucsf.edu/chimerax).[8–11] Standard parameters of this version of software were utilized in the buried volume calculations including a radius of 3.5 Å from the metal center and van Der Waals scaling of 1.17.[12–14]

Electrochemistry. Cyclic voltammetry experiments were conducted under N_2 at 295 ± 3 K using a standard three-electrode setup consisting of a PEEK-encased glassy carbon disc working electrode (Ø = 1 mm; eDAQ), Type 2 graphite rod counter electrode (Ø = 3 mm; Fisher), and Ag/AgCl pseudoreference electrode. The Ag/AgCl pseudoreference electrode was prepared by electrodepositing a film of AgCl onto a silver wire (Ø = 1 mm) using 1 M HCl_{aq}. The wire was stored in a glass compartment containing solvent and electrolyte which was separated from the bulk solution using a porous glass frit (Coralpor®, Ø = 3 mm) connected to the compartment with high temperature PTFE heat shrink tubing (McMaster-Carr; 75665K822). The working electrode was polished in the glove box on a microfiber pad (Buehler MicroCloth™) with 0.25 µm diamond polishing paste (Buehler MetaDi) and lapping oil (Buehler) and thoroughly rinsed with the solvent used in the experiment. A Gamry Reference 1010B potentiostat and Gamry software were used for data collection and analysis. Samples contained 0.1 M $[^nBu_4N][PF_6]$ as the electrolyte, organic solvent (3 mL), and 1.0 mM analyte. The uncompensated solution resistance (R_u) was measured for each sample. All reported voltammograms and redox potentials are *iR* compensated and internally referenced to the $Cp_2Fe^{+/0}$ redox couple (0 V). We conservatively estimate a standard deviation of ±10 mV for all reported redox potentials.[15,16] Digital simulations were performed using DigiElch 8.FD software (ElchSoft through Gamry Instruments).

IR and UV-Vis Spectroscopy. Infrared spectra were recorded on a Thermo Nicolet FT-IR instrument by preparing a KBr pellet or liquid IR cell (CaF_2 windows) in the glove box. Additional FTIR spectra were collected on a Mettler-Toledo ReactIR™ 15 equipped with a liquid nitrogen-cooled mercury cadmium telluride (MCT) detector using a DiComp AgX Fiber conduit detector, which contains a diamond sensor tip sealed with a 99.99% gold ring and encased in Hastelloy C22 sheathing. Mettler-Toledo iC IR™ v4.3 software was used for data collection, processing, and visualization. ReactIR™ experiments were conducted in the same manner as previously described.[17] UV-Vis experiments were carried out using an Agilent Cary 100 spectrophotometer using a custom 1.0 cm quartz cuvette fitted with a Schlenk-type Teflon screw valve.

Gas Chromatography-Mass Spectrometry (GC-MS). Samples were collected and analyzed using an Agilent 6890 Series GC equipped with 5973 MSD GC-MS system with flame ionization detector and ChemStation software. To quantify the formation of octane and 1,5-hexadiene as a result of oxidative C-C coupling from reactions between either $\mathbf{1^H}$ or $\mathbf{1^{Ph}}$ with either *n*-BuLi or $Mg(C_3H_5)Br$, as well as reductive C-C coupling reactions between $\mathbf{[3^H]^{2-}}$ and $\mathbf{[3^{Ph}]^{2-}}$ with 1-bromobutane, the following general procedure was followed: All reactions were performed in a N_2 filled glovebox, and after completion, a known concentration of the reaction mixture was transferred to 2.0 mL SureSTART™ glass screw top GC-MS vials. Samples were analyzed using an injection volume of 1.0 µL, an HP-5MS (5% phenylmethyl siloxane) column, and retention times of 3.202 min for octane and 2.303 min for 1,5-hexadiene. It is important to note that for accurate yield determinations, a calibration curve using authentic samples of octane or 1,5-hexadiene was performed on the same day as the analyses. To maintain consistent reaction conditions and product outcomes, 3 equivalents of the reactants allylMgBr, *n*-BuLi, or 1-bromobutane were added to solutions of either $\mathbf{1^H}$, $\mathbf{1^{Ph}}$, $\mathbf{[3^H]^{2-}}$ or $\mathbf{[3^{Ph}]^{2-}}$.

NMR Spectroscopy. NMR spectroscopic data were recorded on a Bruker Avance III HD 500 MHz spectrometer or Varian Inova 600 MHz spectrometer at 25°C unless otherwise indicated. Spectra for ^1H and ^{13}C were referenced to their respective residual *protio* solvent signal,[18] ^{11}B to external BF_3-OEt_2, and ^7Li to external LiCl, and recorded in parts per million. Deuterated THF was dried over NaK alloy and vacuum transferred prior to use while all other deuterated solvents were degassed via three freeze-pump-thaw cycles and dried over 10% w/v activated 3 Å molecular sieves in an N_2 filled glove box. We stress that the THF-d_8 must be dried over NaK alloy to remove traces of moisture prior to dissolving the reduced species of complexes 1^H or 1^{Ph}. Variable temperature ^1H NMR experiments were performed on a 500 MHz Avance III HD NMR spectrometer in the range between -80 and 20 °C in CD_2Cl_2. Spectra were collected in 10 °C intervals. NMR spectra were fitted by trial and error using the DNMR (dynamic NMR) 7 package in Topspin. The exchange rate constant for each modeled line broadening was used to generate an Eyring plot, see the main text for detailed analysis.

EPR Spectroscopy. X-band (9.65 GHz) EPR spectra were recorded on a Bruker Elexsys spectrometer outfitted with a dual-mode resonator and an Oxford ESR 900 cryostat. The microwave frequency, generated by a Gunn diode, was measured by a frequency counter. A modulation frequency and amplitude of 100 kHz and 0.5 mT pp, respectively, was used. The temperature for EPR measurements was calibrated using a carbon-glass resistor (LakeShore CGR-1-1000) placed at the position of the sample in an EPR tube. EPR simulations were performed by the software SpinCount developed by M. P. Hendrich.[19]

X-Ray Crystallography. Single crystals were selected and mounted using Paratone onto a nylon fiber and cooled to the data collection temperature of 100(2) K with a stream of dry nitrogen gas. X-ray diffraction intensities were collected on a Rigaku XtaLAB Synergy-I diffractometer using CuKα (1.54178 Å) radiation with a HyPIX HPC detector. Structures were refined by full-matrix least squares based on F2 with all reflections (SHELXTL V5.10; G. Sheldrick, Siemens XRD, Madison, WI). Non-hydrogen atoms were refined with anisotropic displacement coefficients, and hydrogen atoms were treated as idealized contributions. SADABS (Sheldrick, 12 G.M. SADABS (2.01), Bruker/Siemens

Area Detector Absorption Correction Program; Bruker AXS: Madison, WI, 1998) absorption correction was applied. Crystallographic data have been deposited with the Cambridge Crystallographic Data Center and are available free of charge through the CCDC online database.

Syntheses

[$H_2B(^{tBu}NHC)_2Mn(CO)_3$] (**1^{H}**)

In the glovebox, [$H_2B(^{tBu}Im)_2$]I (200 mg, 0.52 mmol) and lithium diisopropylamide (113 mg, 1.05 mmol) were added to a vial and dissolved in THF (5 mL). The mixture was stirred at room temperature for 2 h to yield a pale yellow and slightly cloudy solution. [$Mn(CO)_3(NC^{tBu})Br$]$_2$ (136 mg, 0.23 mmol) was then added to the mixture, which immediately turned deep red. After stirring overnight at room temperature, the mixture darkened to deep red-black. The solvent was removed under vacuum to yield a dark, oily, black residue. The residue was dried under vacuum and redissolved in DCM (1 mL), followed by the addition of pentane (4 mL). The solution was filtered through a glass fiber filter plug to remove brown solids, and then dried under high vacuum to give an orange-red oil. This oil was dissolved in methanol (2 mL) and extracted with pentane (5 mL x 15) to remove remaining lithium salts. The pentane extractions were combined and dried to yield a yellow powder (113 mg, 55%). Single crystals suitable for X-ray diffraction were grown via slow evaporation of the product in a biphasic mixture of MeOH and pentane. **^{1}H{^{11}B} NMR** (500 MHz, CD_2Cl_2): δ 6.89 (2H, CH_{NHC}), 6.74 (2H, CH_{NHC}), 3.00 (1H, BH), 1.67 (18H, ^{t}Bu), -7.99 (1H, B-H--Mn) ppm. **^{1}H NMR** (500 MHz, C_6D_6): δ 6.51 (s, 1H), 6.29 (s, 1H), 4.07 – 2.99 (br, m, 1H), 1.41 (s, 11H), -7.33 (br, d, *J* = 92.0 Hz, 1H). **^{1}H NMR** (500 MHz, CD_3CN): δ 7.01 (2H, CH_{NHC}), 6.81 (2H, CH_{NHC}), 2.88 (1H, free BH), 1.66 (18H, ^{t}Bu), -8.13 (1H, μ-BH) ppm. **^{1}H NMR** (500 MHz, THF-*d*$_8$): δ 7.03 (2H, CH_{NHC}), 6.79 (2H, CH_{NHC}), 3.16 (1H, free BH), ca. 1.72 (18H, ^{t}Bu, overlapping with THF-d$_8$), -7.83 (1H, μ-BH) ppm. **^{13}C NMR** (150 MHz, C_6D_6): δ 230.0 ($CO_{trans\text{-}NHC}$), 221.7 ($CO_{cis\text{-}NHC}$), 182.85 (N*C*N), 121.61 (HC=CH), 118.40 (HC=CH), 57.10 (*C*(CH_3)$_3$), 31.15 (C(*C*H_3)$_3$). **^{13}C NMR** (150 MHz, THF-*d*$_8$): δ 230.0 ($CO_{trans\text{-}NHC}$), 221.7 ($CO_{cis\text{-}NHC}$), 182.85 (N*C*N), 121.61 (HC=CH), 118.40 (HC=CH), 57.10 (*C*(CH_3)$_3$), 31.15 (C(*C*H_3)$_3$). **^{11}B{^{1}H) NMR** (160 MHz, CD_2Cl_2): δ -15.18 ppm. **^{11}B NMR** (160 MHz, C_6D_6): δ -14.76 ppm. **^{11}B NMR** (160 MHz, CD_3CN): δ -15.21 ppm. **^{11}B NMR** (160 MHz, THF-*d*$_8$): δ -15.17 ppm. **IR** (KBr): 2013, 1921, 1897 cm^{-1} (ν_{CO}). **UV-Vis** (THF, $\varepsilon_{\lambda max}$($M^{-1}cm^{-1}$)): ε_{393} = 740 ± 30, ε_{368} = 580 ± 30. **Anal. Calcd for $C_{17}H_{24}N_4O_3BMn$ (%)**: Found C: 51.05, H: 5.91, N: 13.50. Expected: C: 51.28, H: 6.08, N: 14.07.

[Na(2.2.2)crypt][**2^{H}**]

Sodium naphthalenide (NaNap) was prepared in the glovebox by stirring naphthalene (19.6 mg, 0.15 mmol) with a large excess of Na^0 chunks in a vial with THF (2 mL), which immediately turns the mixture a dark green color. After 30 min of stirring, the solution was filtered through a glass fiber filter plug and added to a yellow solution of **1^{H}** (48.5 mg, 0.12 mmol) and [2.2.2]cryptand (46.8 mg, 0.12 mmol) dissolved in THF (1 mL). The resulting dark green solution was stirred for 2.5 h, filtered through a glass fiber filter plug, and dried under high vacuum to yield a dark green residue. Adding ether/pentane (1:1) and scraping the product residue before drying again under high vacuum yielded a dark green powder (40 mg, 41%). Crystals suitable for X-ray diffraction were grown by dissolving in a minimal amount of THF and performing a THF/pentane vapor diffusion in the freezer at -30°C. **UV-Vis** (THF, $\varepsilon_{\lambda max}$($M^{-1}cm^{-1}$)): ε_{685} = 330 ± 30, ε_{353} = 870 ± 40. **Anal. Calcd for $C_{35}H_{60}BMnN_6NaO_9$ (%)**: Found: C: 52.37, H: 7.67, N: 9.80. Expected: C: 52.70, H: 7.58, N: 10.54.

[K(2.2.2)crypt][**2^{H}**]

In the glovebox, **1^{H}** (34.4 mg, 0.086 mmol) and [2.2.2]cryptand (37.1 mg, 0.099 mmol) were combined in a vial with THF (3 mL). Solid KC_8 (13.2 mg, 0.98 mmol) was added to the yellow solution, and the mixture immediately turned a dark forest-green color. The solution was stirred for 3 h before filtering through a glass fiber filter plug. The solvent was evaporated under high vacuum to concentrate the dark green solution. Purification was achieved by dissolving in a minimal amount of THF and crystallizing via THF/pentane vapor diffusion to yield dark green crystals, which are suitable for X-ray diffraction (47 mg, 67%). The sample was not analyzed any further.

[K(18-c-6(THF)$_x$)][**2^{H}**]

In the glovebox, **1^{H}** (31.6 mg, 0.079 mmol) and 18-crown-6 (28.6 mg, 0.11 mmol) were combined in a vial with THF (3 mL). Solid KC_8 (12.8 mg, 0.95 mmol) was added to the yellow solution, and the mixture immediately turned dark forest-green. The solution was stirred for 3 h before filtering through a glass fiber filter. The solvent was evaporated under high vacuum to concentrate the dark green solution. Purification was achieved by crystallizing the product via THF/pentane vapor diffusion to yield dark green crystals (31 mg, 56%). The sample was not analyzed any further.

[Na(THF)$_x$]$_2$[**3^{H}**]

In the glovebox, **1^{H}** (30.1 mg, 0.0756 mmol) and pure Na^0 chunks were stirred together in THF-*d$_8$* (0.7 mL) at room temperature for 3 h. The solution, after turning a dark red brown, was then passed through a glass fiber filter into a J Young tube for NMR analysis. Due to its incredibly sensitive nature, the product was not purified or isolated any further. **^{1}H NMR** (500 MHz, THF-*d*$_8$): δ 6.90 (2H, CH_{NHC}), 6.77 (2H, CH_{NHC}), 3.38 (br, m, 1H, BH), 2.58 (br, m, 1H, BH), 1.86 (18H, ^{t}Bu) ppm. **^{11}B NMR** (160 MHz, THF-*d*$_8$): δ -7.87 ppm. **^{13}C NMR** (126 MHz, THF-*d*$_8$): δ 261.71 (CO_{NHC}), 205.46 (N*C*N), 122.72 (HC=CH), 115.70 (HC=CH), 56.85 ($\mathit{C}(CH_3)_3$), 31.14 ($C(\mathit{C}H_3)_3$) ppm.

Reactions with *n*-BuLi

Synthesis of [Li(THF)$_x$][**2^{Ph}**] and octane

In the glovebox, **1^{Ph}** (7.5 mg, 0.014 mmol) and THF (1 mL) were added to a vial equipped with a blackened Teflon stir bar. To the stirring orange solution, *n*-BuLi (2.78 M in hexanes; 5 µL, 0.014 mmol) was added, and a green solution was immediately observed. The sample was retained for UV-Vis analyses. The absorbance at 662 nm (ε = 570) indicates that **[2^{Ph}]$^-$** is generated in nearly quantitative yield (ca. 96%), formulated as [Li(THF)$_x$][**2^{Ph}**], where x = 3 or 4.[17] In a second reaction, 3 equivalents of *n*-BuLi were added following identical reaction conditions, and an aliquot taken for GC-MS analysis revealed octane production in (11% yield, average of three trials). In a third reaction, a vial containing a green solution from the reaction between **1^{Ph}** and *n*-BuLi was transferred out of the glovebox and exposed to air, which instantly turned the green solution orange. For **1^{Ph}** purification, the bulk orange solution was dried under high vacuum, and the residue was dissolved in DCM. The DCM solution was pipetted through a glass fiber filter plug to remove a dark brown precipitate and dried under high vacuum to give an orange powder, which was dissolved in Et_2O. The Et_2O solution was pipetted through a glass fiber filter plug to remove insoluble impurities and dried under vacuum to yield **1^{Ph}** recovered in 42% yield via ^{1}H NMR spectroscopy in CD_2Cl_2[7] vs. added 1,3,5-trimethoxybenzene (TMB) as an internal standard.

Synthesis of [Li(THF)$_2$][**1^H-Ac$_{eq}$**]

In the glovebox, **1^H** (9.8 mg, 0.025 mmol) and THF (1 mL) were added to a vial equipped with a blackened Teflon stir bar. To the stirring yellow solution, *n*-BuLi (2.78 M in hexanes; 8.8 µL, 0.025 mmol) was added, and a red-brown solution was observed within 5 min. The solution was stirred for an additional 30 min, pipetted through a glass fiber filter plug, and dried under vacuum to yield the orange-brown powder [Li(THF)$_2$][**1^H-Ac$_{eq}$**] in 36% yield, as determined via ^{1}H NMR spectroscopy in THF-*d$_8$* *vs.* 1,3,5-trimethoxybenzene (TMB) internal standard. **^{1}H NMR** (500 MHz, THF-*d*$_8$, 25°C): δ 6.74 (2H, CH_{NHC}), 6.59 (2H, CH_{NHC}), 3.61 (8H, free THF), 3.14 – 2.71 (m, 1H, free BH), 2.44 (2H, $CH_2CH_2CH_2CH_3$), 1.77 (8H, free THF), 1.60 (18H, ^{t}Bu), 1.22 (2H, $CH_2CH_2CH_2CH_3$), 1.10 (2H, $CH_2CH_2CH_2CH_3$), 0.75 (3H, $CH_2CH_2CH_2CH_3$), -4.40 (br, 1H, µ-BH) ppm. **^{1}H{^{11}B} NMR** (500 MHz, THF-*d*$_8$, 25°C): δ 6.74 (2H, CH_{NHC}), 6.59 (2H, CH_{NHC}), 3.61 (8H, free THF), 3.03 – 3.01 (d, $^2J_{HH}$ = 10.3 Hz, 1H, free BH), 2.44 (2H, $CH_2CH_2CH_2CH_3$), 1.77 (8H, free THF), 1.60 (18H, ^{t}Bu), 1.22 (2H, $CH_2CH_2CH_2CH_3$), 1.11 (2H, $CH_2CH_2CH_2CH_3$), 0.75 (3H, $CH_2CH_2CH_2CH_3$), -4.22 – -4.24 (d, $^2J_{HH}$ = 10.6 Hz, 1H, µ-BH) ppm. **^{11}B{^{1}H} NMR** (160 MHz, THF-*d$_8$*): δ -13.51 ppm. **^{7}Li{^{1}H} NMR** (194 MHz, THF-*d$_8$*): δ 1.10 ppm. **IR** (THF): 1889, 1797, 1748 cm^{-1} (ν_{CO}). **^{13}C NMR** (126 MHz, THF-*d*$_8$, 25°C): δ 200.2 (NCN), 120.7 (NC=CN), 115.8 (NC=CN), 60.5 ($CH_2CH_2CH_2CH_3$), 56.3 ($C(CH_3)_3$), 28.4 ($CH_2CH_2CH_2CH_3$), 31.5 ($C(CH_3)_3$), 23.9 ($CH_2CH_2CH_2CH_3$), 14.6 ($CH_2CH_2CH_2CH_3$) ppm. The ^{13}C resonances for the CO ligands are detected only at -80 °C, while the CO-Bu ligand could not be resolved between -80 °C and 25 °C. **^{13}C NMR** (126 MHz, THF-*d*$_8$, -80°C): δ 240.7 (CO), 229.2 (CO), 198.5 (NCN), 198.0 (NCN), 119.0 (NC=CN), 118.2 (NC=CN), 114.4 (NC=CN), 112.9 (NC=CN), 59.0 ($CH_2CH_2CH_2CH_3$), 54.4 ($C(CH_3)_3$), 54.2 ($C(CH_3)_3$), 26.8 ($CH_2CH_2CH_2CH_3$), 29.6, 29.2 ($C(CH_3)_3$), 22.3 ($CH_2CH_2CH_2CH_3$), 13.2 ($CH_2CH_2CH_2CH_3$) ppm. **^{1}H NMR** (500 MHz, THF-*d*$_8$, -80°C): δ 6.77, 6.70 (2H, CH_{NHC}), 6.59, 6.49 (2H, CH_{NHC}), 2.90 (br, 1H, free BH), 2.37 (2H, $CH_2CH_2CH_2CH_3$), 1.54 (18H, ^{t}Bu), 1.22 (buried; 2H, $CH_2CH_2CH_2CH_3$), 1.05 (2H, $CH_2CH_2CH_2CH_3$), 0.73 (3H, $CH_2CH_2CH_2CH_3$), -4.60 (br, 1H, µ-BH) ppm. See discussion in main text about intramolecular acyl exchange behavior and loss of mirror plane symmetry in low temperature NMR spectra of **[1^H-Ac$_{eq}$]$^-$**. **Anal. Calcd for $C_{29}H_{47}N_4O_5BMnLi$ (%)**: Found C: 52.755, H: 7.309, N: 11.545. Expected: C: 57.63, H: 7.84, N: 9.27. The discrepancies in EA for this very air sensitive sample are likely exacerbated by loss of the butyl fragment and solvent (THF).

Synthesis and crystallization of [Li(12-c-4)][**1^H-Ac$_{eq}$**]

In the glovebox, **1^H** (5.3 mg, 0.013 mmol), 12-crown-4 (2.6 µL, 0.013 mmol), and DME (1 mL) were added to a vial equipped with a blackened Teflon stir bar. To the stirring yellow solution, *n*-BuLi (2.78 M in hexanes; 4.8 µL, 0.013 mmol) was added, and a red-brown solution was observed within 5 min. The solution was stirred for an additional 30 min, pipetted through a glass fiber filter plug, concentrated under high vacuum, and set up to crystallize (DME/pentane vapor diffusion) at -35°C. After two weeks, tiny, crystalline orange blocks formed of the equatorial acylation product [Li(12-c-4)]**[1^H-Ac$_{eq}$]$^-$**.

Reactions with allylmagnesium bromide

Synthesis of [**2^{Ph}**]$^-$ and 1,5-hexadiene

In the glovebox, **1^{Ph}** (18.0 mg, 0.0327 mmol) and THF (1.4 mL) were added to a vial equipped with a blackened Teflon stir bar. $Mg(C_3H_5)Br$ (1.0 M in Et_2O, 0.1 mL, 0.1 mmol) was added to the stirring orange solution, and within 20 min the mixture turned dark green. The sample was stirred for 1 h and retained for GC-MS and UV-Vis analyses. The dark green mixture was filtered through a syringe filter, and an aliquot was taken for GC-MS analysis, revealing an average yield of 48% for 1,5-hexadiene production (three trials). In a second reaction, **1^{Ph}** (12.0 mg, 0.0218 mmol), THF (1.2 mL), and $Mg(C_3H_5)Br$ (1.0 M in Et_2O, 0.07 mL, 0.0654 mmol) were combined under similar conditions to generate a

dark green solution, which was dried under high vacuum, and the residue dissolved in MeCN for UV-Vis analysis. The absorbance at 669 nm (ε = 220 M^{-1} cm^{-1}) indicates that $\mathbf{[2^{Ph}]^-}$ is generated in a 34% yield.

Reactions with 1-bromobutane

Synthesis of [Na(THF)$_x$][$\mathbf{2^{Ph}}$] and octane

In the glovebox, $\mathbf{1^{Ph}}$ (15.6 mg, 0.0283 mmol), THF (2.0 mL), and excess fresh chunks of Na^0 were added to a vial equipped with a blackened Teflon stir bar. After 3.5 h, a red-brown color-tinted solution formed, indicative of doubly reduced $\mathbf{[3^{Ph}]^{2-}}$. $CH_3(CH_2)_3Br$ (3.4 µL, 0.0311 mmol) was added to the stirring red-brown solution, and immediately the mixture turned dark green. The dark green solution was pipetted through a glass fiber filter plug, and [Na(THF)$_x$][$\mathbf{2^{Ph}}$] was generated in a nearly quantitative yield (ca. 99%), as observed by UV-Vis spectroscopy using the absorbance at 665 nm (ε = 570 M^{-1} cm^{-1}). In a separate reaction, 3 equivalents of $CH_3(CH_2)_3Br$ were added under identical reaction conditions and an aliquot taken for GC-MS analysis revealed a 35% yield of octane (average of three trials).

Synthesis of [Na(THF)$_x$][$\mathbf{1^H}$-$\mathbf{Ac_{eq}}$]

In the glovebox, $\mathbf{1^H}$ (15.5 mg, 0.0389 mmol), THF (2.0 mL), and excess fresh chunks of Na^0 were added to a vial equipped with a blackened Teflon stir bar. After 3 h, a red-brown color-tinted solution formed, indicative of doubly reduced $\mathbf{[3^H]^{2-}}$. $CH_3(CH_2)_3Br$ (4.6 µL, 0.043 mmol) was added to the stirring red-brown solution, and within 10 mins the mixture turned a bright orange color, and a white precipitate (NaBr) was observed. The orange solution was pipetted through a glass fiber filter plug and dried under vacuum to yield the orange powder [Na(THF)$_x$][$\mathbf{1^H}$-$\mathbf{Ac_{eq}}$] in a 67% yield via ^{1}H NMR spectroscopy in THF-d_8 vs. added 1,3,5-trimethoxybenzene (TMB) as an internal standard. ^{1}H and ^{11}B NMR spectra of [Na(THF)$_x$][$\mathbf{1^H}$-$\mathbf{Ac_{eq}}$] closely match chemical shifts observed for [Li(THF)$_2$][$\mathbf{1^H}$-$\mathbf{Ac_{eq}}$], which is generated from the reaction of $\mathbf{1^H}$ with *n*-BuLi as described above. See discussion in main text about intramolecular acyl exchange behavior and loss of mirror plane symmetry in low temperature NMR spectra of $\mathbf{[1^H\text{-}Ac_{eq}]^-}$. In a separate reaction, 3 equivalents of $CH_3(CH_2)_3Br$ were added following identical reaction conditions, and an aliquot of the orange filtered solution was taken for GC-MS analysis, revealing traces (< 1%) of octane.

Reactions with 6-Iodo-1-hexene

Synthesis of Iodomethylcyclopentane via [$\mathbf{1^{Ph}}$]$^-$

Sodium naphthalenide (NaNap) was prepared in the glovebox by stirring naphthalene (1.4 mg, 0.011 mmol) with a large excess of Na^0 chunks in a vial with THF (1 mL), which immediately turns the mixture a dark green color. After 30 min of stirring, the solution was filtered through a glass fiber filter plug and added to a yellow solution of $\mathbf{1^{Ph}}$ (4.8 mg, 0.0087 mmol; 10 mol%). After 2 h, the slightly brighter green solution is indicative of reduced $\mathbf{[2^{Ph}]^-}$. The solution was dried and the solid was redissolved in 0.55 mL THF-d_8 before transferring to a J. Young tube. To this solution, 6-iodo-1-hexene (13.5 uL, 0.0933 mmol) was added along with 1,3,5-trimethoxybenzene (TMB) as an internal standard. The mixture was stirred in an NMR tube spinner overnight for 18 h at which point the green solution turned orange, indicative of neutral $\mathbf{1^{Ph}}$. ^{1}H NMR confirms the presence of $\mathbf{1^{Ph}}$ and iodomethylcyclopentane in >99% and 57% yield, respectively (5.8 turnovers; average of three trials).

Synthesis of Iodomethylcyclopentane via [1^H]$^-$

Sodium naphthalenide (NaNap) was prepared in the glovebox by stirring naphthalene (1.8 mg, 0.014 mmol) with a large excess of Na^0 chunks in a vial with THF (1 mL), which immediately turns the mixture a dark green color. After 30 min of stirring, the solution was filtered through a glass fiber filter plug and added to a yellow solution of **1^H** (4.9 mg, 0.012 mmol). After 2 h, the slightly brighter green solution is indicative of reduced **[2^H]**$^-$. The solution was dried and the solid was redissolved in 0.55 mL THF-d_8 before transferring to a J-Young tube. To this solution, 6-iodo-1-hexene (18.5 uL, 0.128 mmol) was added along with 1,3,5-trimethoxybenzene (TMB) as an internal standard. Within 5 min the solution turns from green to orange. The mixture was stirred in an NMR tube spinner overnight for 18 h. at which point the solution turned yellow, indicative of neutral **1^H**. ^{1}H NMR confirms the presence of **1^H** and iodomethylcyclopentane in 41% and 51% yield, respectively (5.0 turnovers; average of three trials). Evidence of the 1-hexene acylated **[1^H-Ac_{eq}]** product were also seen after about 20 minutes (Fig 27), with a final yield of 5% after 18 h (Fig S28).

Electrochemistry

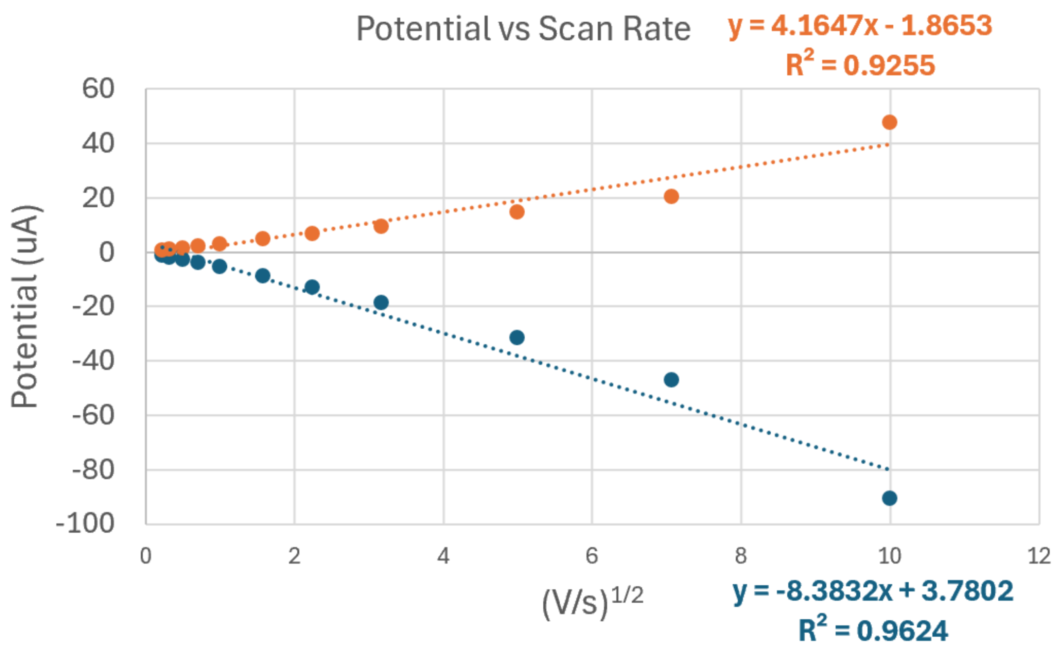

Figure S1 - Graph of $E_{1/2}$ vs $v^{1/2}$. Data derived from the variable scan rate CVs of the $Mn^{I/0}$ redox couple of **1^H** in THF (see Figure 4B, main text).

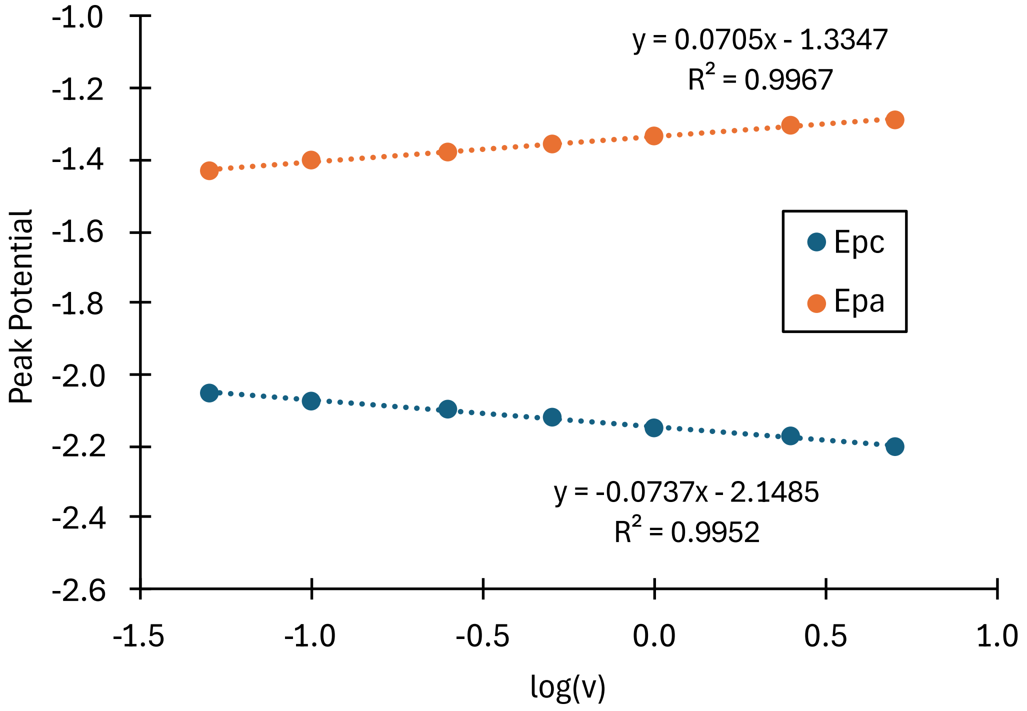

Figure S2 - Plot of current vs log(v). Data derived from the variable scan rate CVs of the $Mn^{I/0}$ redox couple of $\mathbf{1^H}$ in THF (see Figure 4B, main text).

Table S1. Digital Simulation Parameters

**E_q Mechanism**	
Mn(I) + e^- ⇌ Mn(0)	E = −1.95 V, α = 0.50, k_S = 5.0 × 10^{-4} cm/s
**$\underline{E_i E_i}$ Mechanism**	
Mn(I) + e^- → Mn(0)	E = −1.95 V, α = 0.40, k_S = 1.4 × 10^{-3} cm/s
Mn(I) + e^- ← Mn(0)	E = −1.95 V, α = 0.58, k_S = 6.4 × 10^{-4} cm/s
**Model Parameters**	
D = 1 × 10^{-5} cm^2/s (all species)	T = 298.2 K
$[Mn(I)]_o$ = 0.001 M	Noise level = 0%
Area = 0.008 cm^2	Expansion Factor (x-grid) = 0.5
Potential step = 0.001 V	Truncation Error = 1 × 10^{-5} %
R_u = 0 Ω	X_{max} / $(Dt)^{1/2}$ = 6
C_{dl} = 0 F	

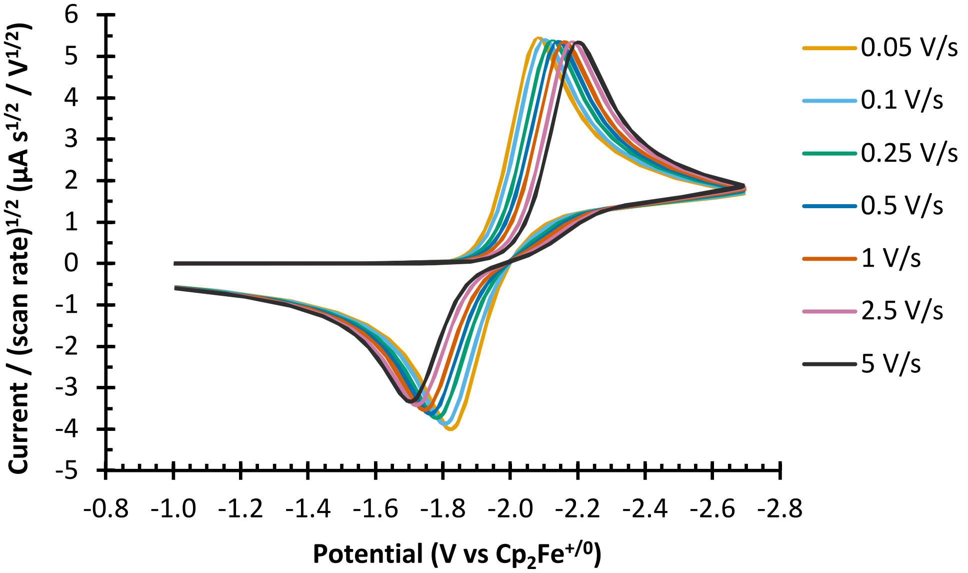

Figure S3 – Simulated voltammograms for an E_q mechanism.

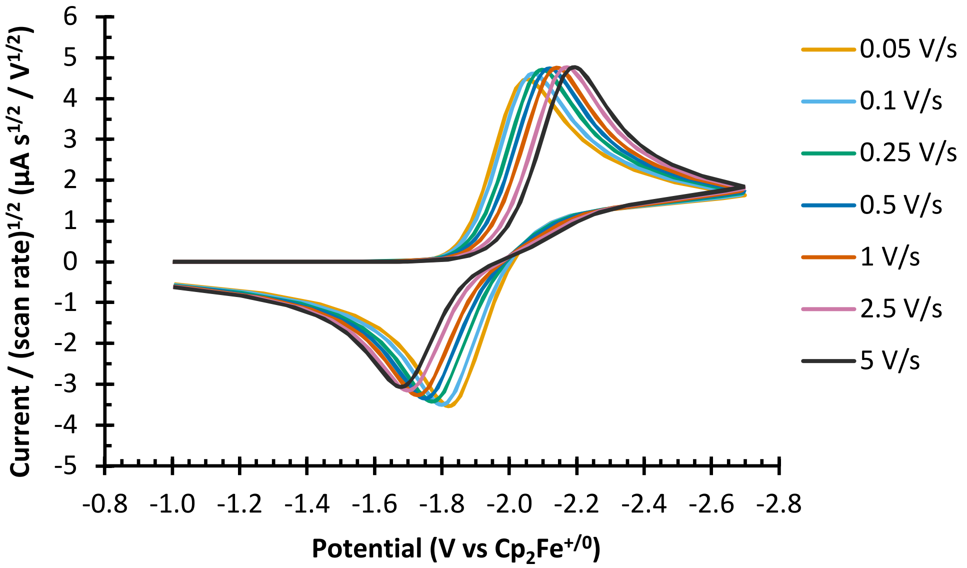

Figure S4 – Simulated voltammograms for an E_iE_i mechanism.

Table S2. Comparison of Simulated and Experimental Peak Potentials

υ (V/s)	E_q Mechanism			E_iE_i Mechanism			Experimental		
	E_{pc} (V)	E_{pa} (V)	ΔE_p (mV)	E_{pc} (V)	E_{pa} (V)	ΔE_p (mV)	E_{pc} (V)	E_{pa} (V)	ΔE_p (mV)
0.05	−2.085	−1.823	262	−2.053	−1.820	233	−2.053	−1.835	218
0.1	−2.102	−1.805	297	−2.071	−1.800	271	−2.078	−1.810	268

0.25	−2.126	−1.782	344	−2.098	−1.771	327	−2.108	−1.785	323
0.5	−2.143	−1.764	379	−2.119	−1.750	369	−2.118	−1.763	355
1	−2.161	−1.746	415	−2.141	−1.728	413	−2.152	−1.737	415
2.5	−2.185	−1.722	463	−2.170	−1.700	470	−2.178	−1.710	468
5	−2.203	−1.704	499	−2.192	−1.678	514	−2.203	−1.695	508

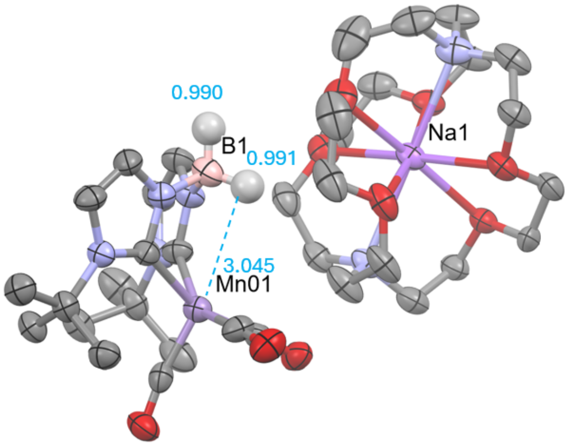

Figure S5 - Molecular structure of [Na(2.2.2)crypt][**2^H**] (50% probability ellipsoids with most hydrogens omitted for clarity).

NMR Spectra

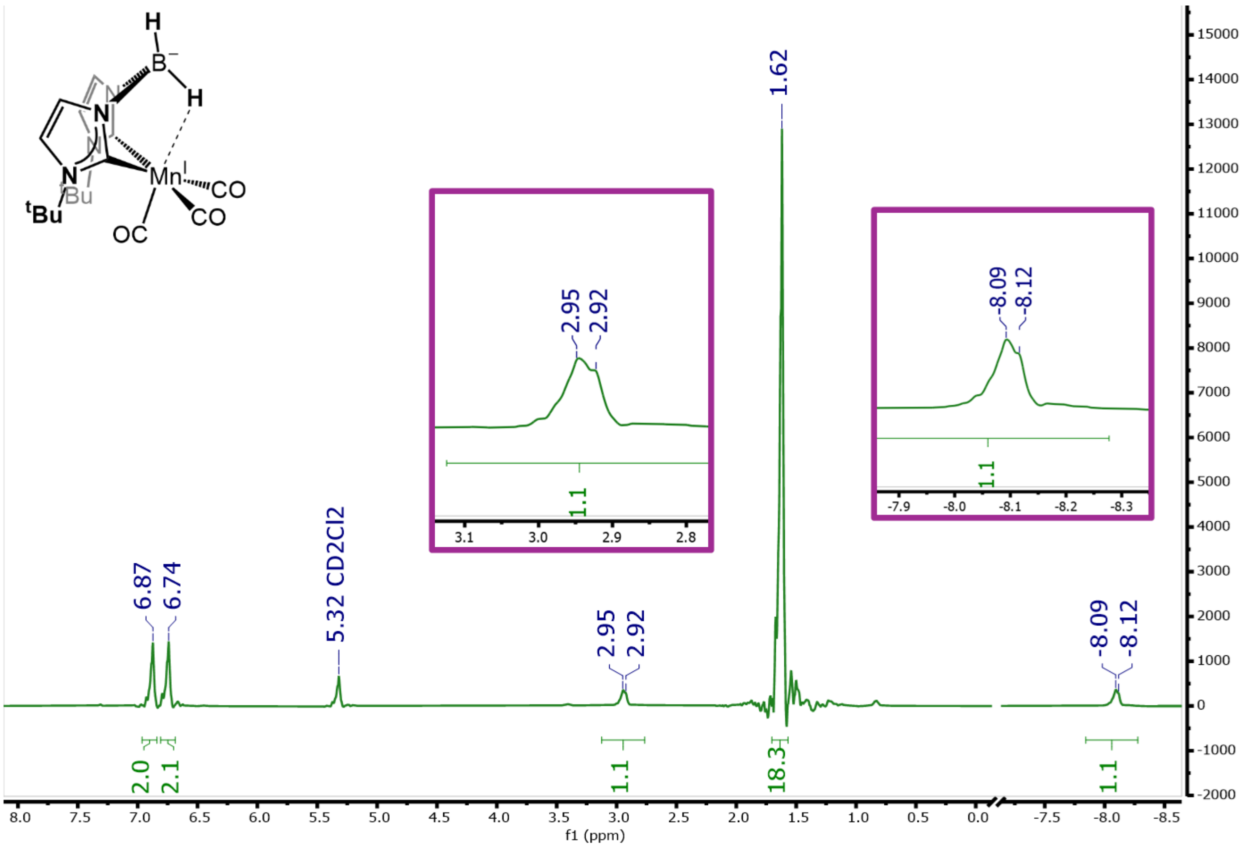

Figure S6 - $^{1}H\{^{11}B\}$ NMR spectrum of **1^{H}** (500 MHz, CD_2Cl_2) at -50°C.

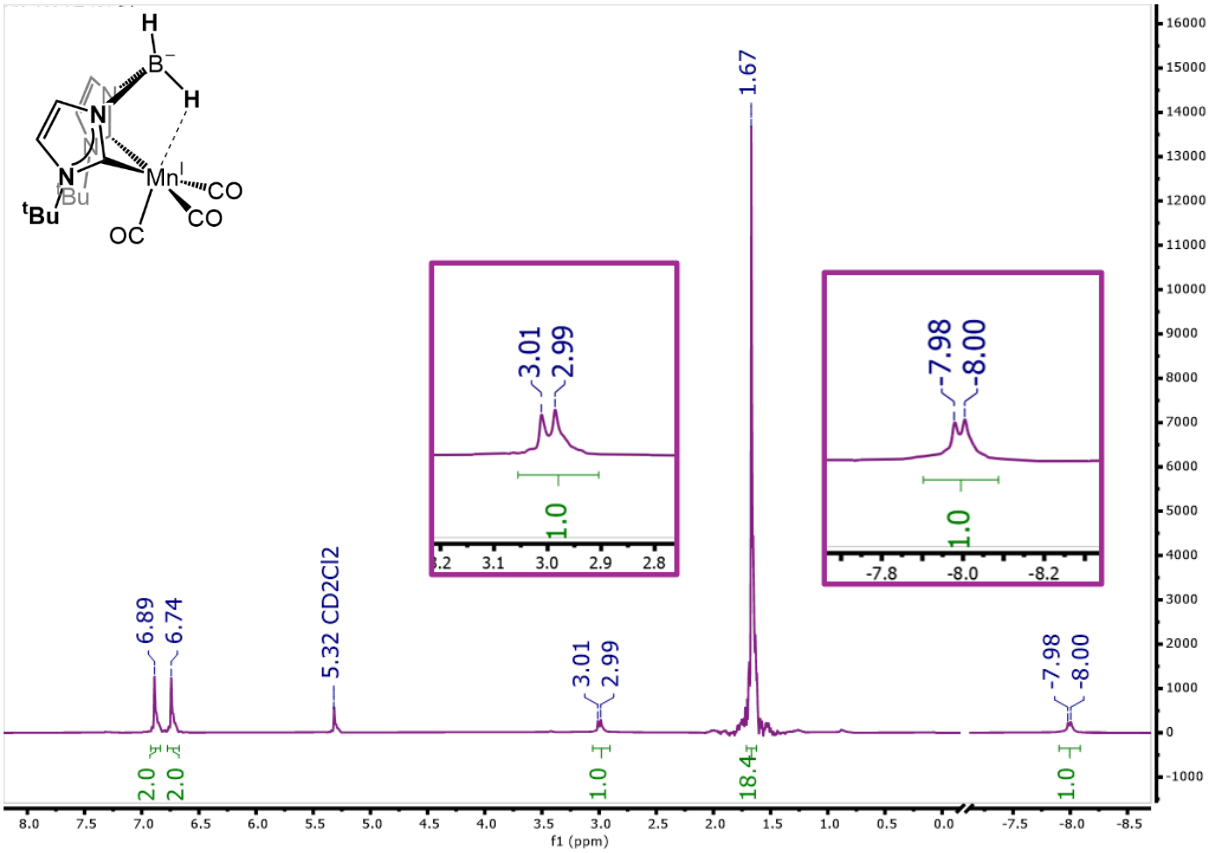

Figure S7 - $^{1}H\{^{11}B\}$ NMR spectrum of **1^H^** (500 MHz, CD_2Cl_2) at 20°C.

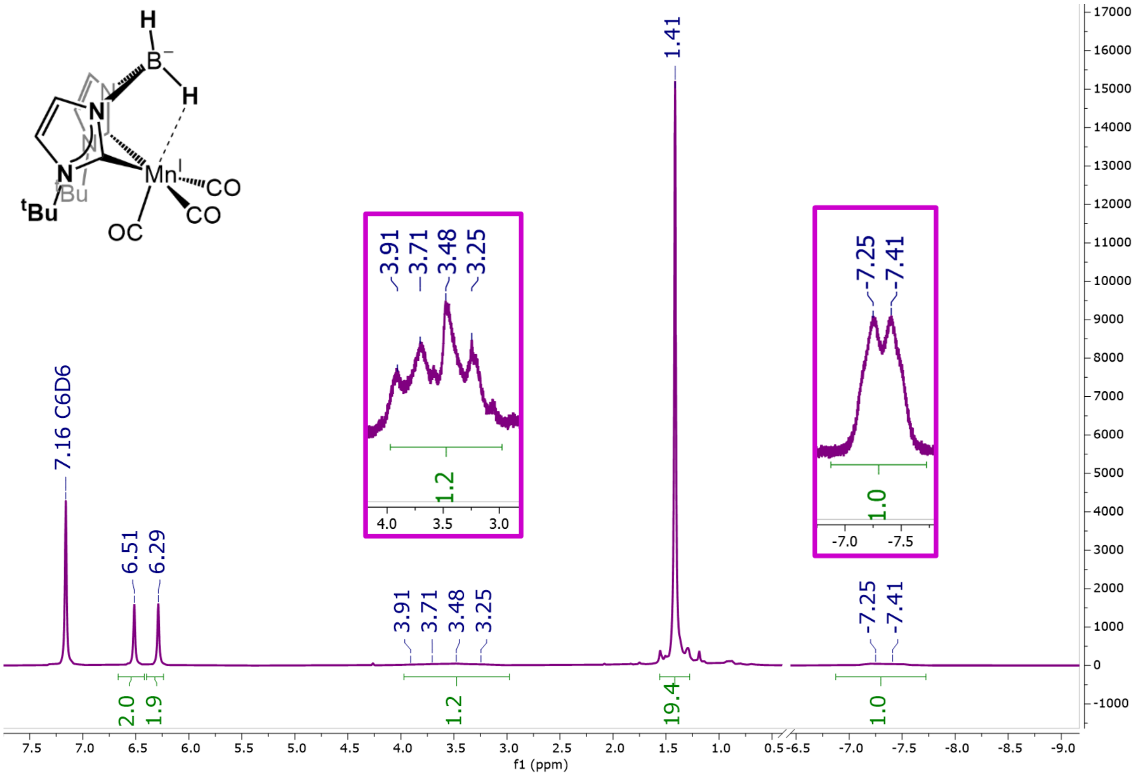

Figure S8 - ^{1}H NMR spectrum of **1^{H}** (500 MHz, C_6D_6) at 25°C.

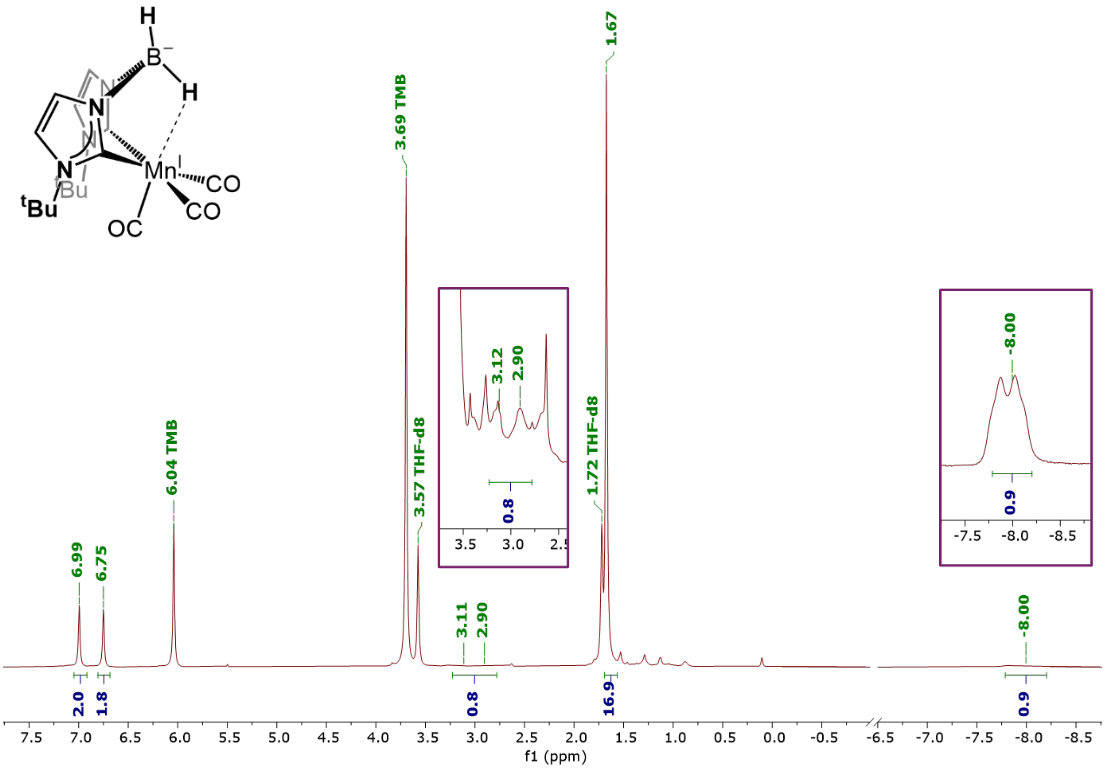

Figure S9 - ^{1}H NMR spectrum of **1^{H}** (500 MHz, THF-*d$_8$*) at 25°C. TMB = 1,3,5-trimethoxybenzene.

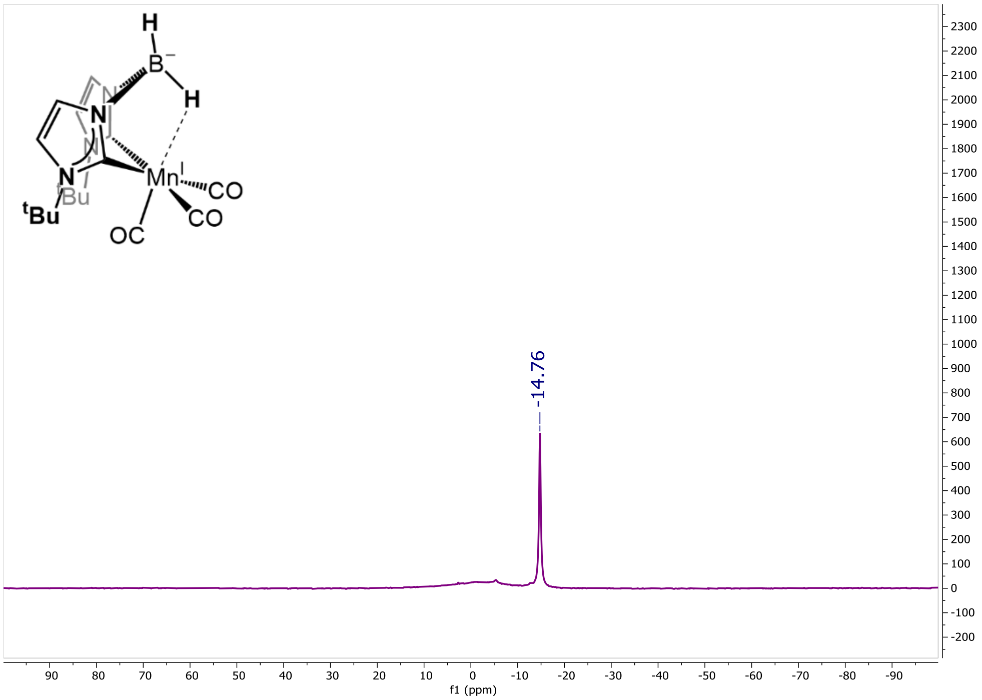

Figure S10 - Background-corrected ^{11}B NMR spectrum of **1^H** (160 MHz, C_6D_6) at 25°C.

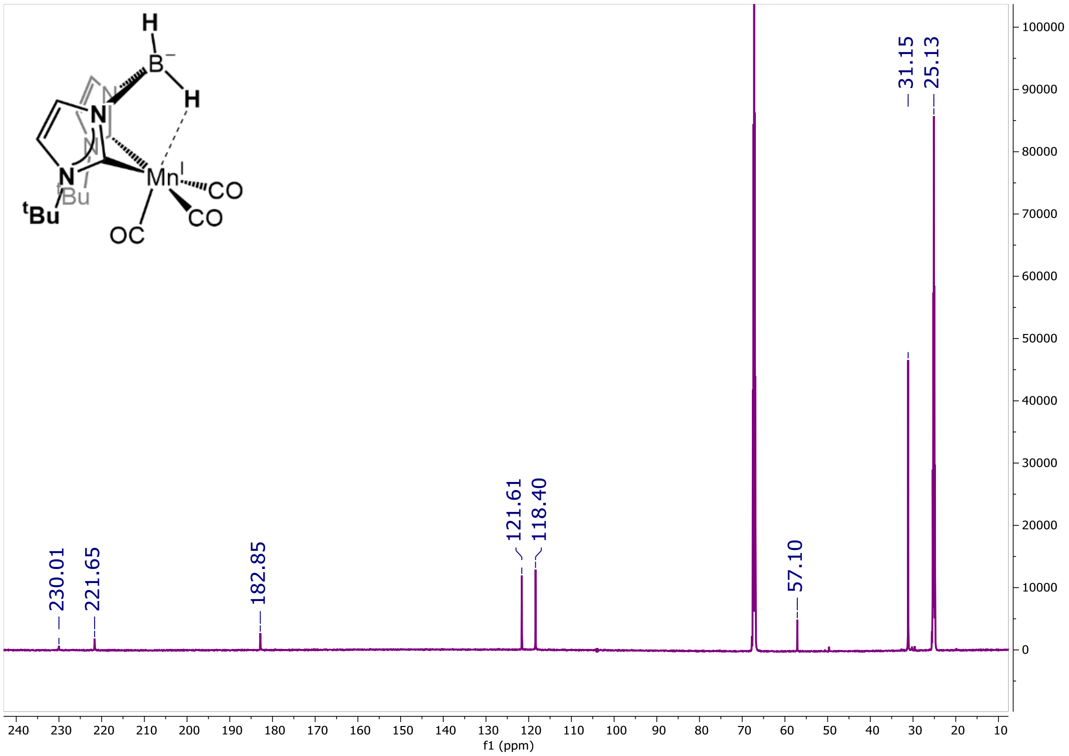

Figure S11 - ^{13}C NMR spectrum of **1^H** (150 MHz, THF-d_8, 25°C).

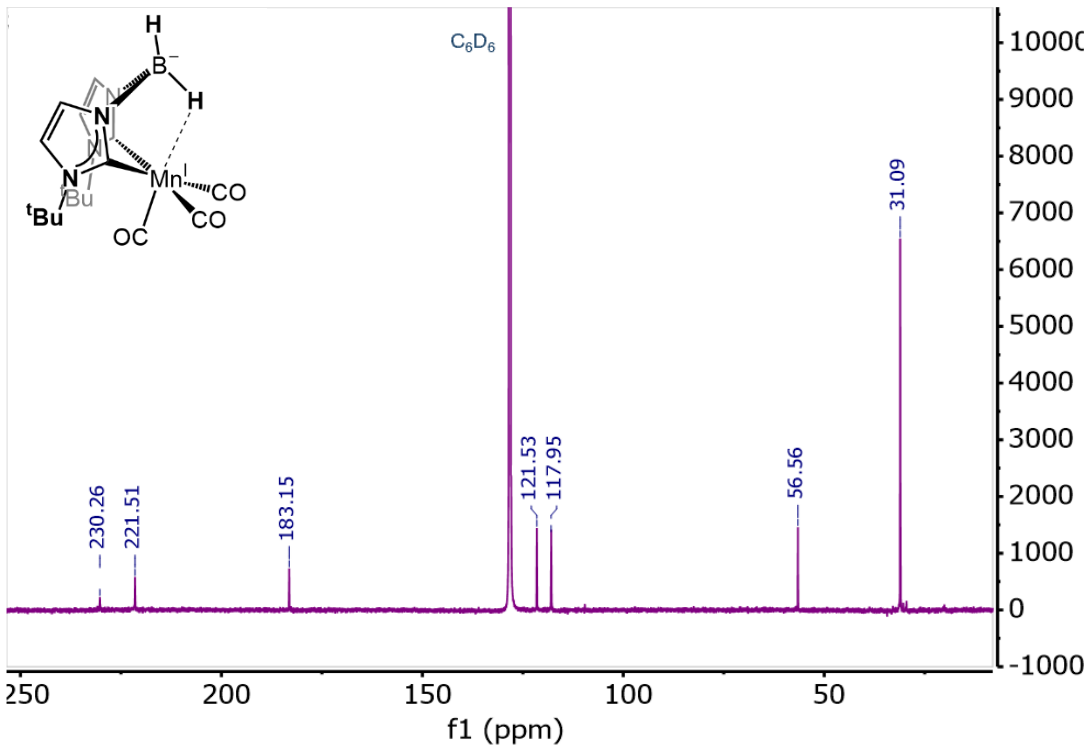

Figure S12 - ^{13}C NMR spectrum of **1^{H}** (150 MHz, C_6D_6, 25°C).

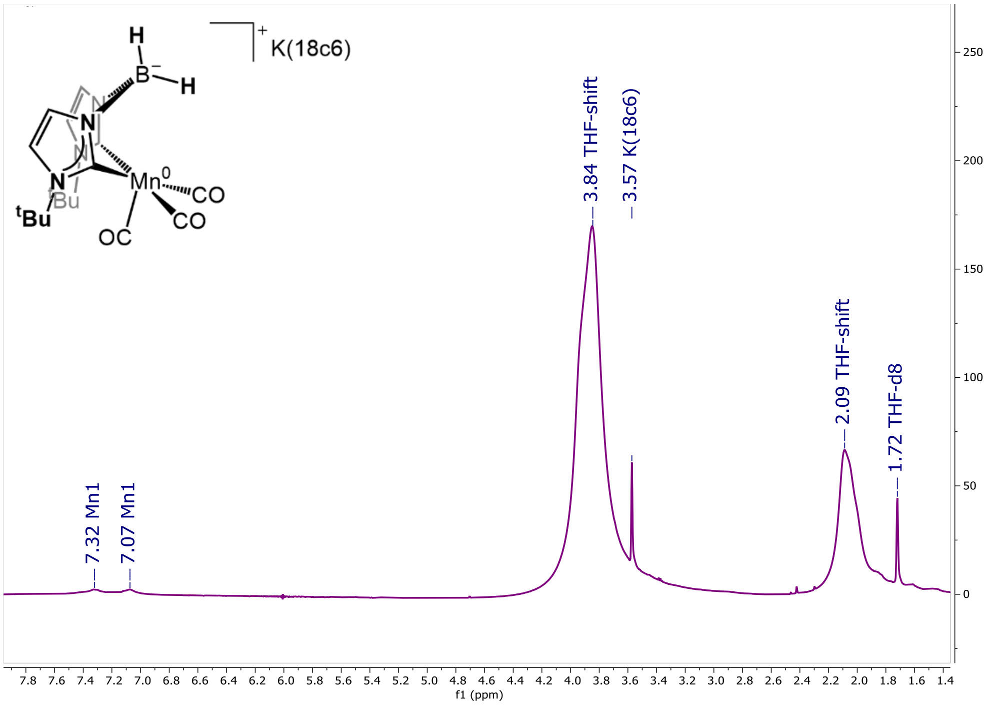

Figure S13 - ^{1}H NMR of [K(18c6)][**2^{H}**] (600 MHz, THF-d_8) at 25°C. Trace amounts of **1^{H}** are observable at 7.07 and 7.32 ppm.

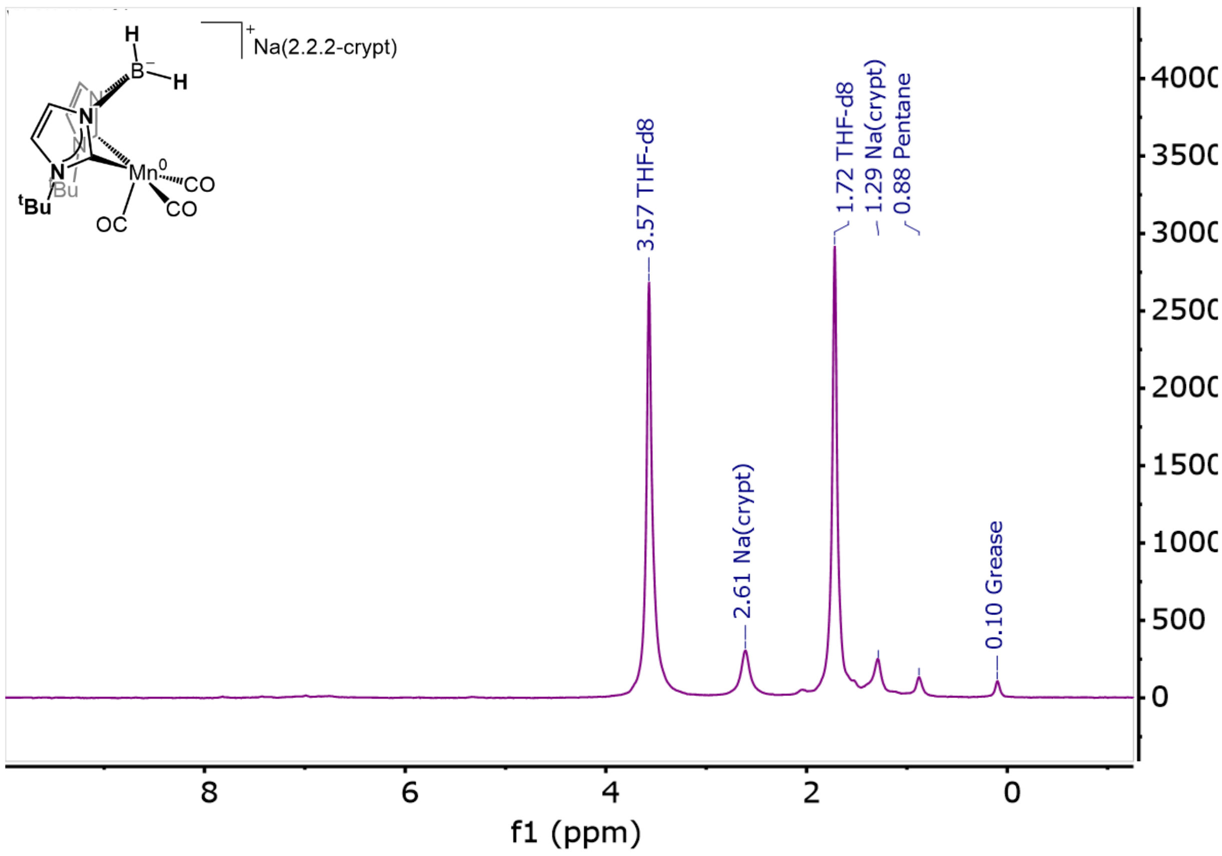

Figure S14 - H NMR of [Na(crypt)][**2^H**] (500 MHz, THF-d_8) at 25°C.

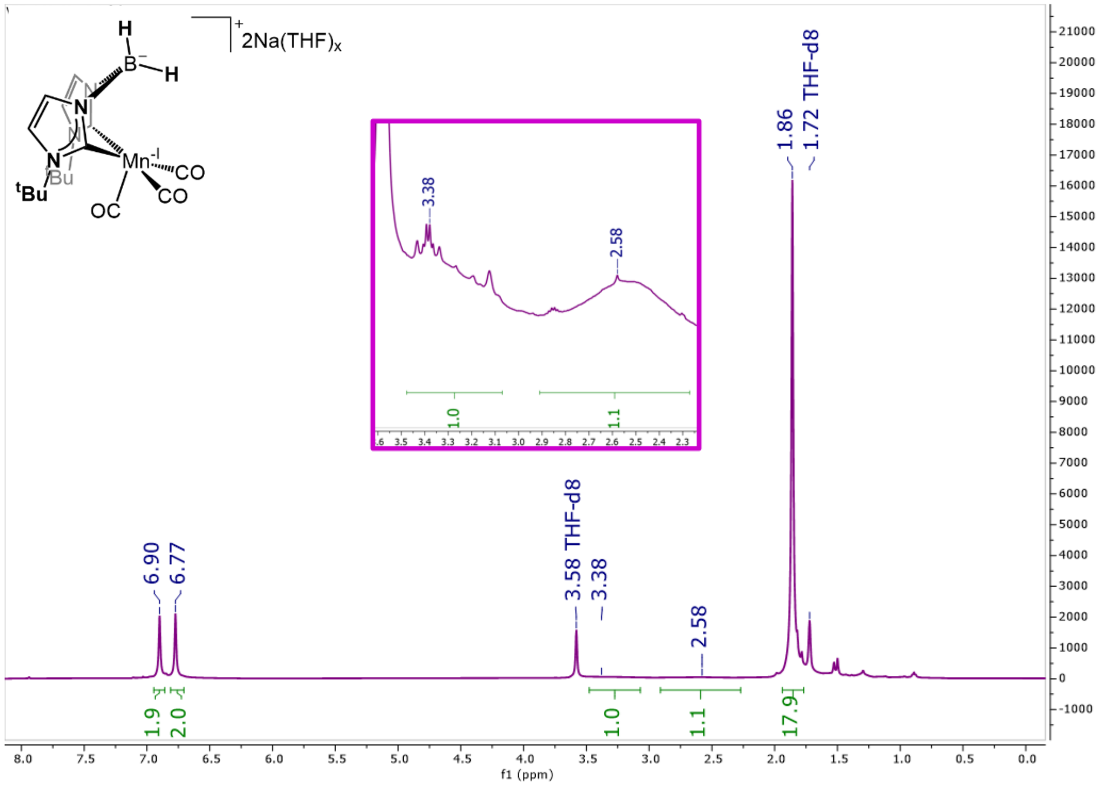

Figure S15 - ^{1}H NMR of $[Na(THF)_x]_2[\mathbf{3^H}]$ (500 MHz, THF-d_8) at 25°C.

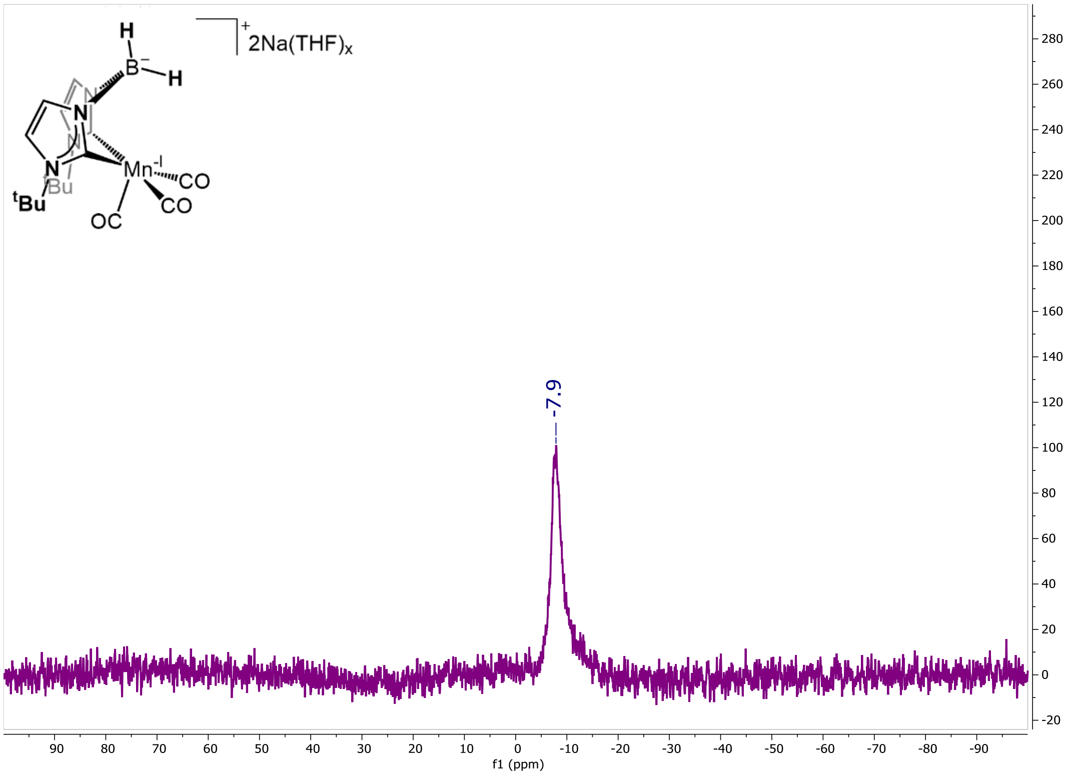

Figure S16 - Background corrected ^{11}B NMR spectrum of $[Na(THF)_x]_2[\mathbf{3^H}]$ (160 MHz, THF-d_8) at 25°C.

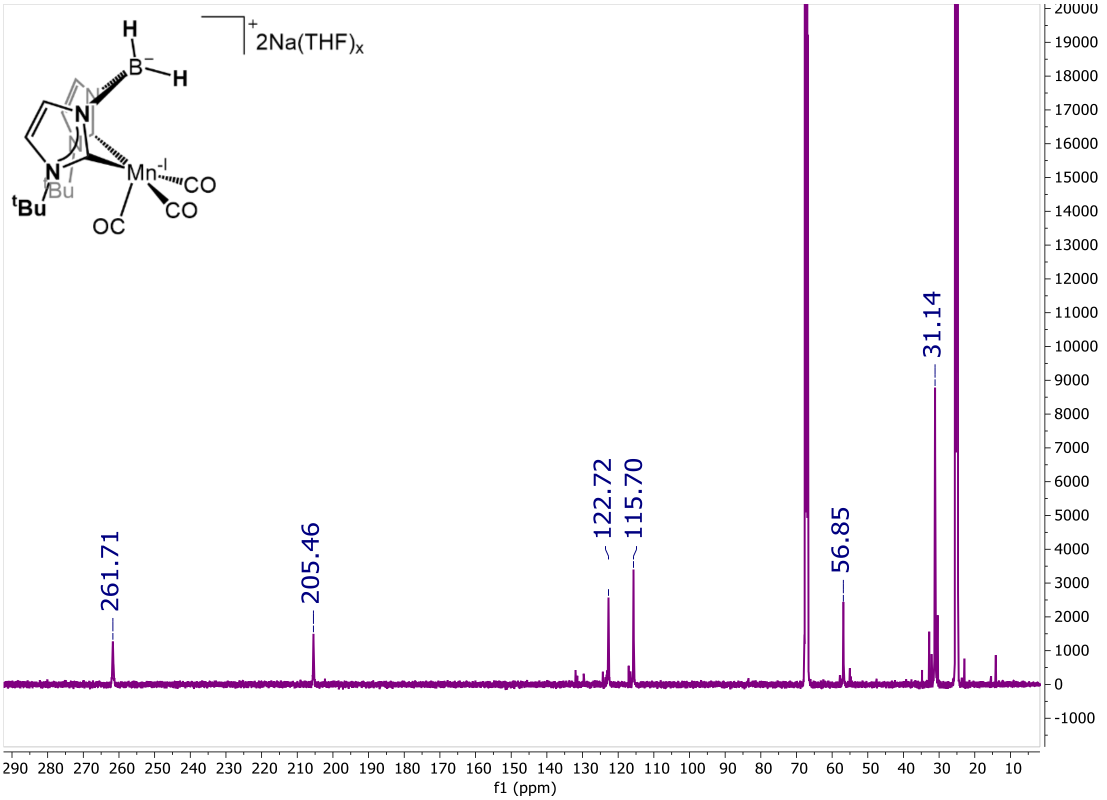

Figure S17 - ^{13}C NMR spectrum of $[Na(THF)_x]_2$[**3^{H}**] (150 MHz, THF-d_8, 25°C).

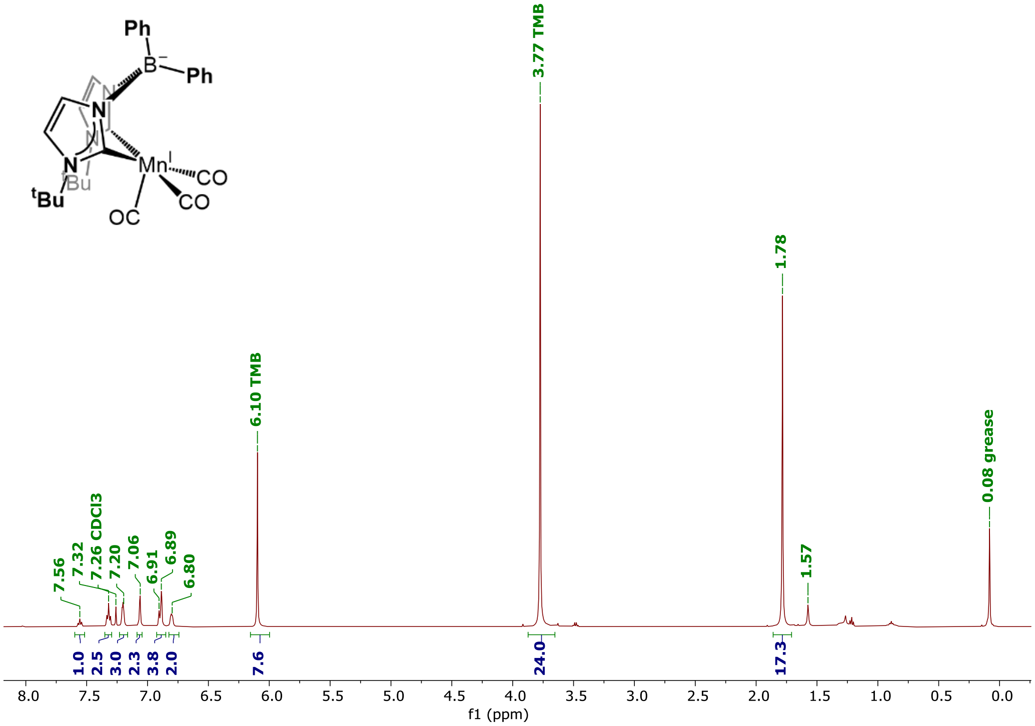

Figure S18 - ^{1}H NMR spectrum of **1^{Ph}** (500 MHz, $CDCl_3$, 25°C) recovered after purification from a reaction of **1^{Ph}** with *n*-BuLi. The internal standard, 1,3,5-trimethoxybenzene (TMB), was added to determine the yield of **1^{Ph}**.

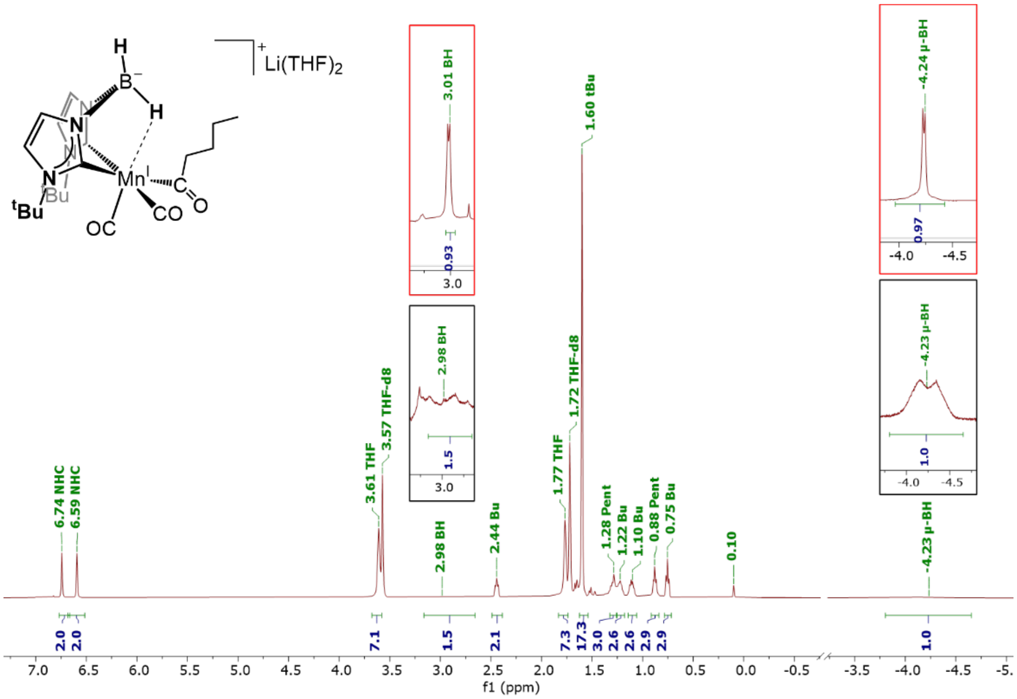

Figure S19 - ^{1}H NMR spectrum of [Li(THF)$_2$][**1^H-Ac$_{eq}$**] (500 MHz, THF-d_8, 25°C) with an insert (black box) for peak clarity of the bridging B-H proton (ca. -4.40 ppm) and the free B-H proton (ca. 2.98 ppm), both of which are broad. These signals sharpen with ^{11}B decoupling, as shown in the red inserts (μ-BH ca. -4.20 ppm, $^2J_{HH}$ = 10.6 Hz and free B-H ca. 3.02 ppm, $^2J_{HH}$ = 10.3 Hz).

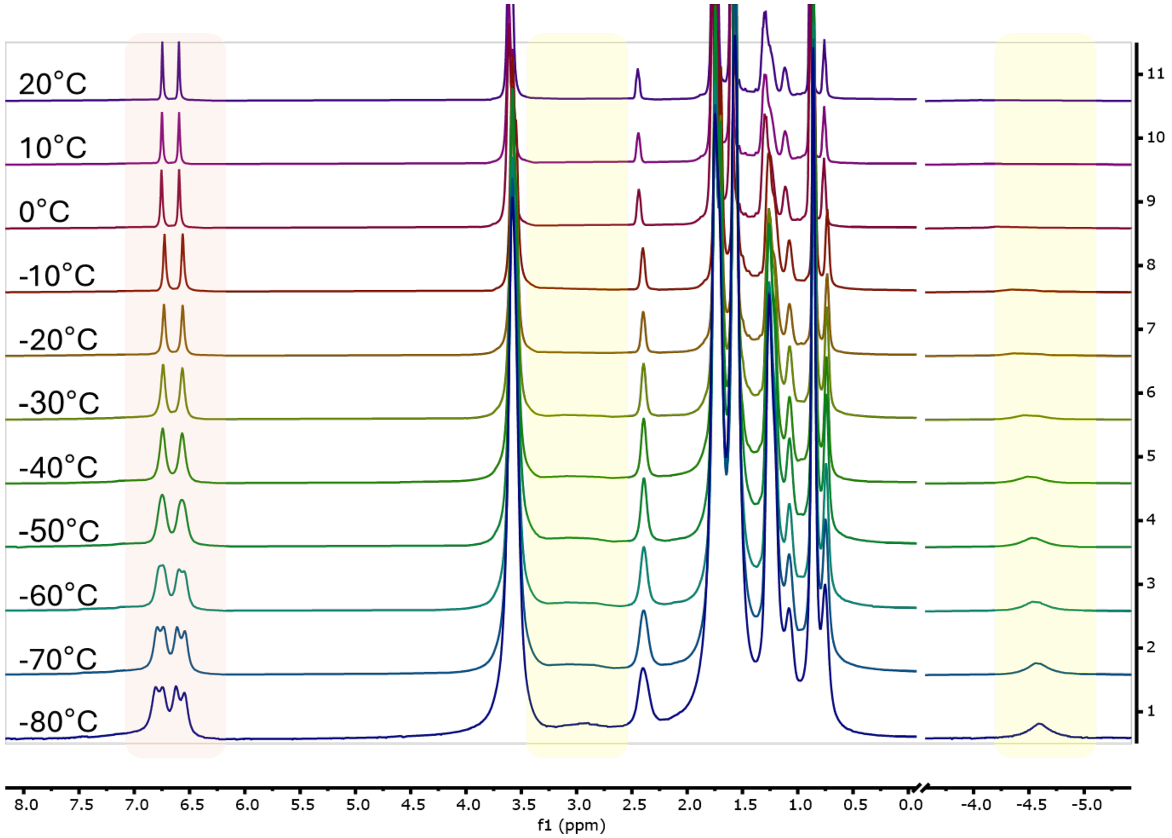

Figure S20 – Stacked VT ^{1}H NMR spectra of $[Li(THF)_2]$[$\mathbf{1^{H}}$-**Ac**$_{\mathbf{eq}}$] (500 MHz, THF-d_8, -80°C to 25°C) with the NHC region highlighted in pink and the BH_2 regions (free vs. μ-BH) in yellow.

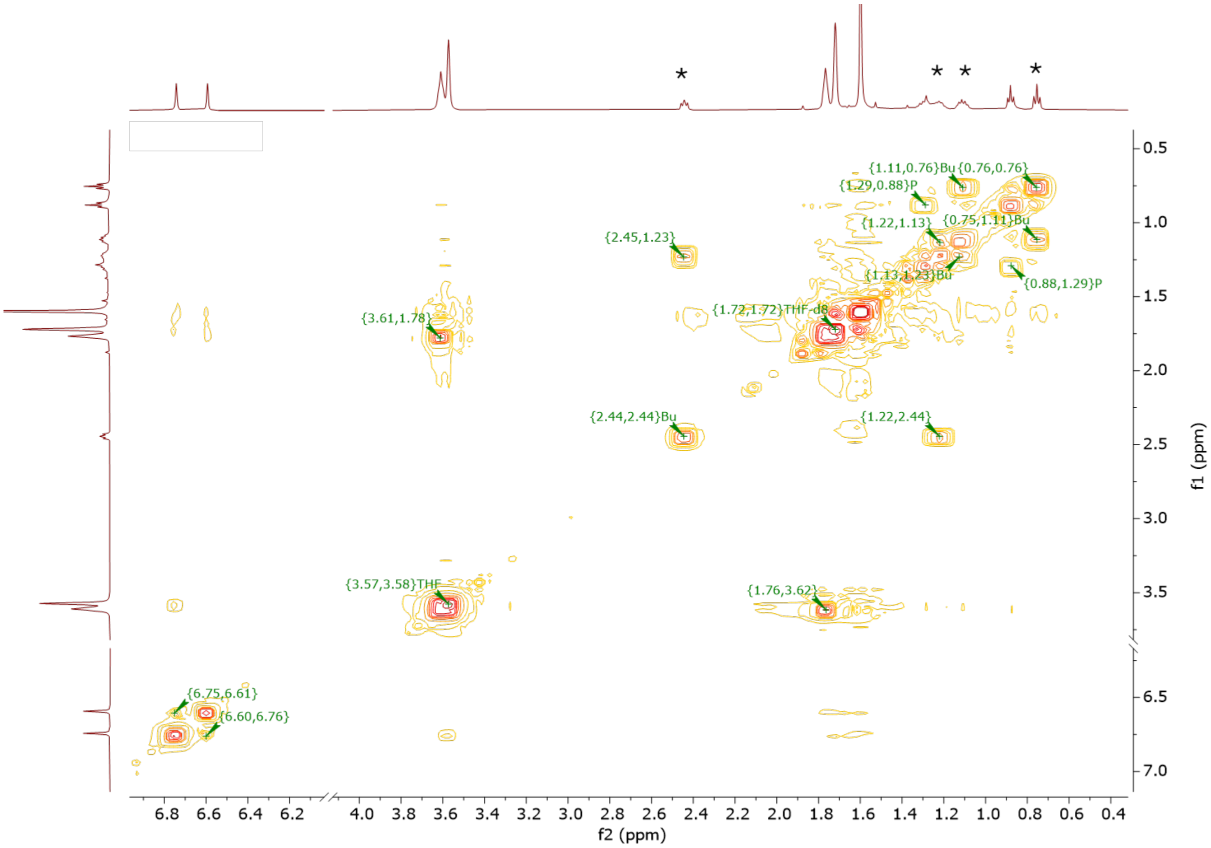

Figure S21 - ^1H-^1H COSY NMR spectrum of $[Li(THF)_2]$[**1^{H}-Ac$_{eq}$**] (500 MHz, THF-d_8, 25°C). Asterisks indicate the butyl chemical shifts.

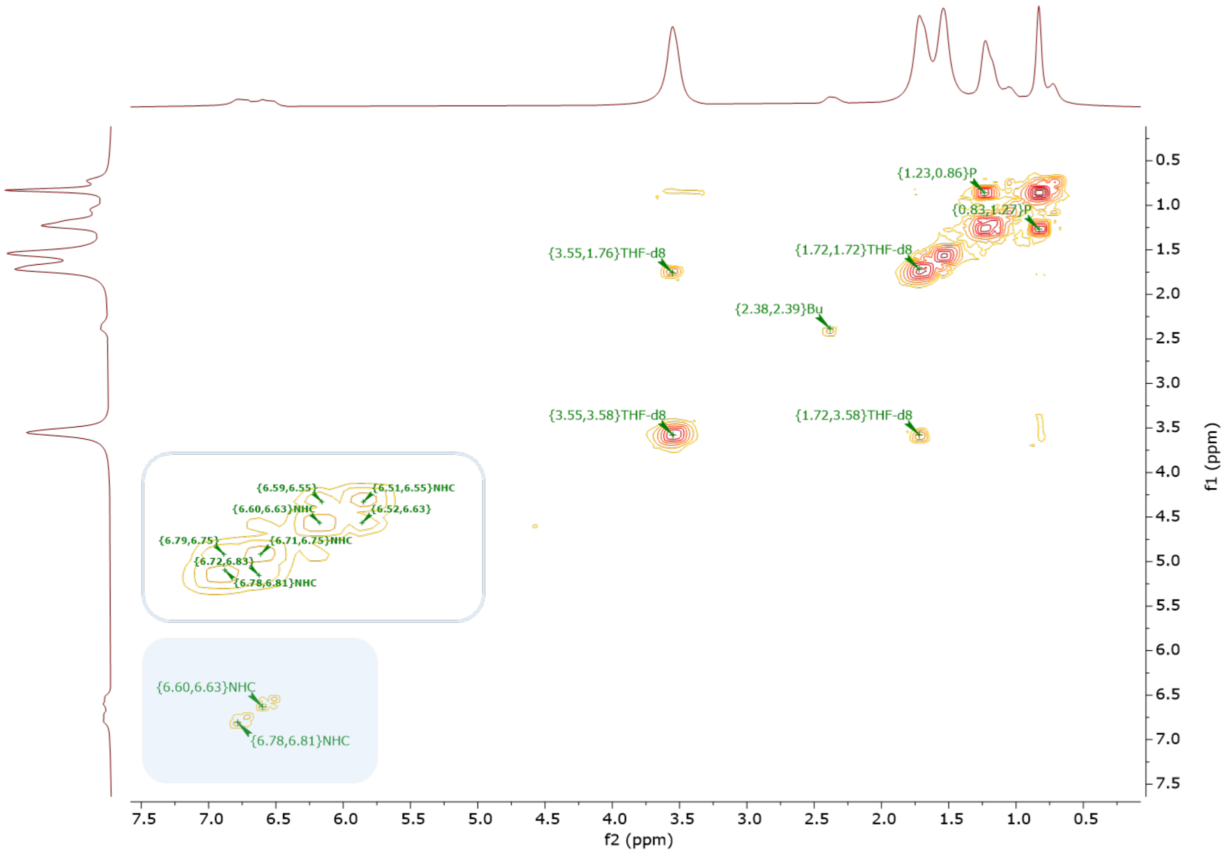

Figure S22 - ^{1}H-^{1}H COSY NMR spectrum of $[Li(THF)_2]$[**1^{H}-Ac$_{eq}$**] (500 MHz, THF-d_8, -80°C) with the NHC region (inset, blue box) showing cross peaks between the new pairs of NHC resonances observed at low temperature.

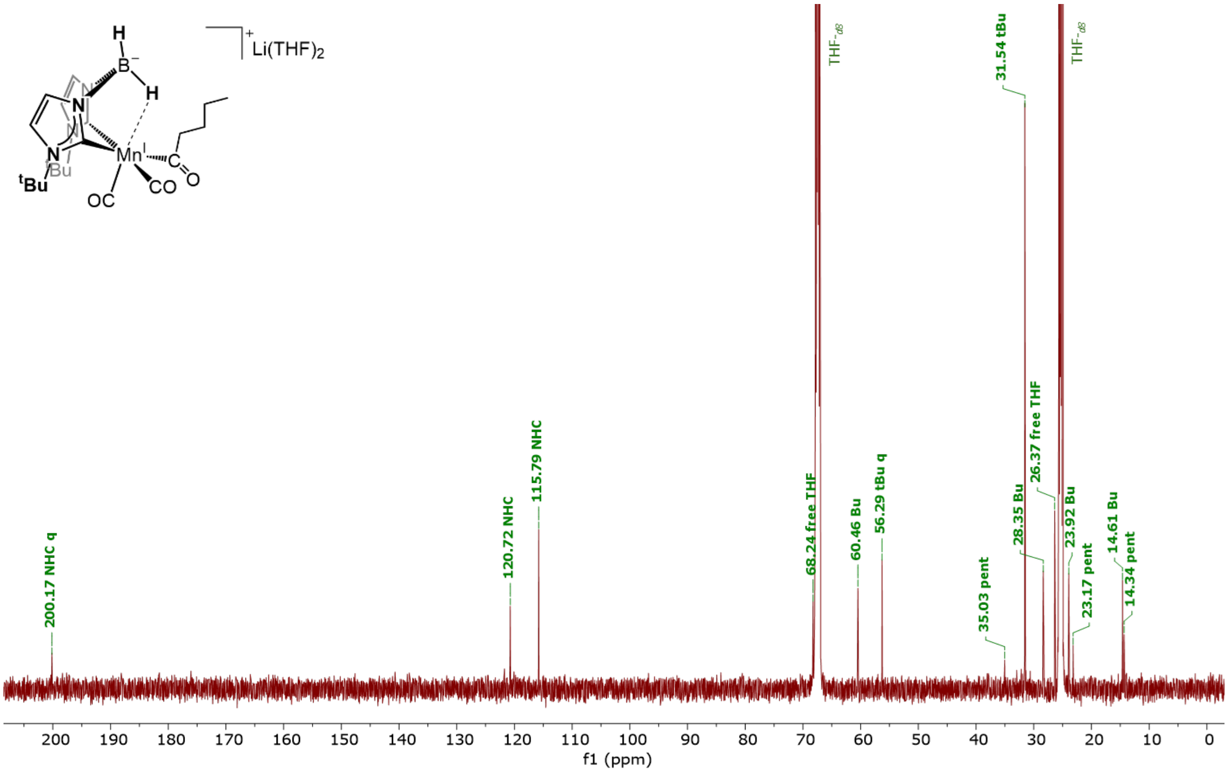

Figure S23 - ^{13}C NMR spectrum of [Li(THF)$_2$][**1^{H}-Ac$_{eq}$**] (126 MHz, THF-d_8, 25°C).

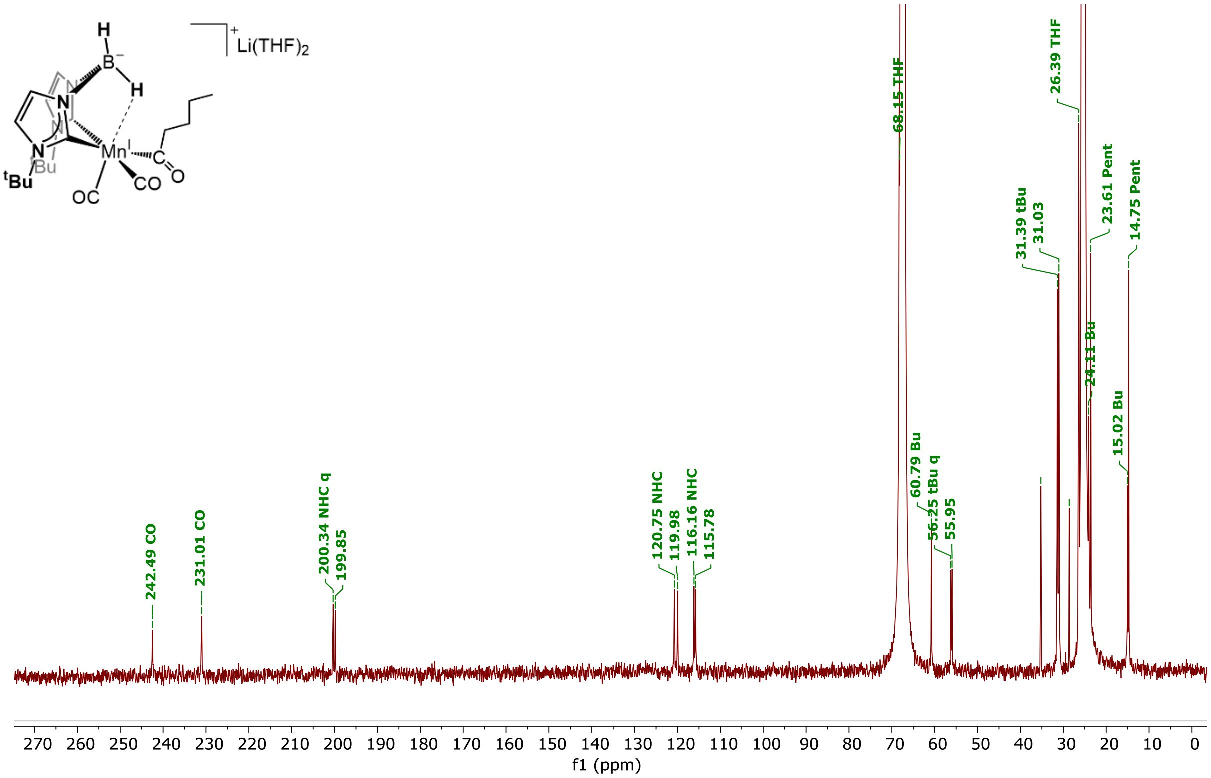

Figure S24 - ^{13}C NMR spectrum of [Li(THF)$_2$][**1^{H}-Ac$_{eq}$**] (126 MHz, THF-d_8, -80°C).

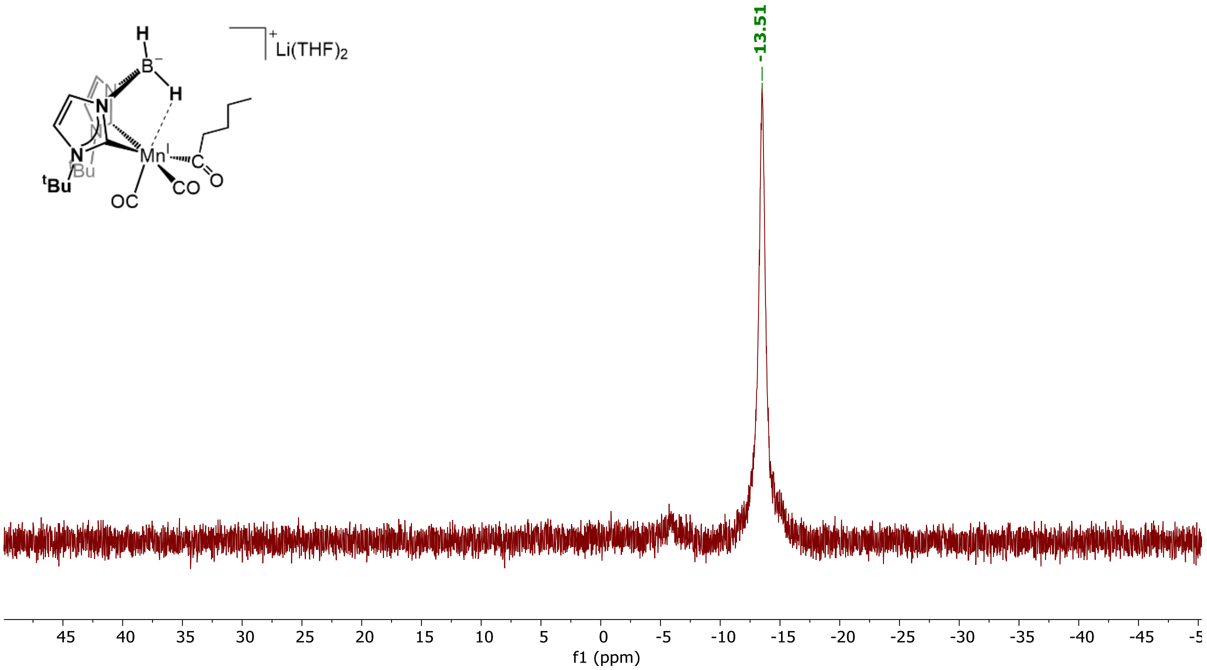

Figure S25 - Background corrected $^{11}B\{^{1}H\}$ NMR spectrum of [Li(THF)$_2$][**1^{H}-Ac$_{eq}$**] (160 MHz, THF-d_8, 25°C).

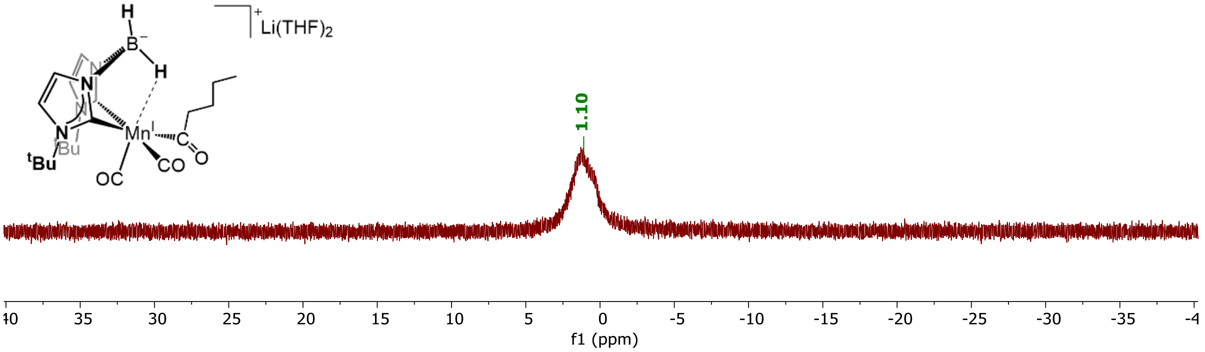

Figure S26 - $^{7}Li\{^{1}H\}$ NMR spectrum of [Li(THF)$_2$][**1^{H}-Ac$_{eq}$**] (194 MHz, THF-d_8, 25°C).

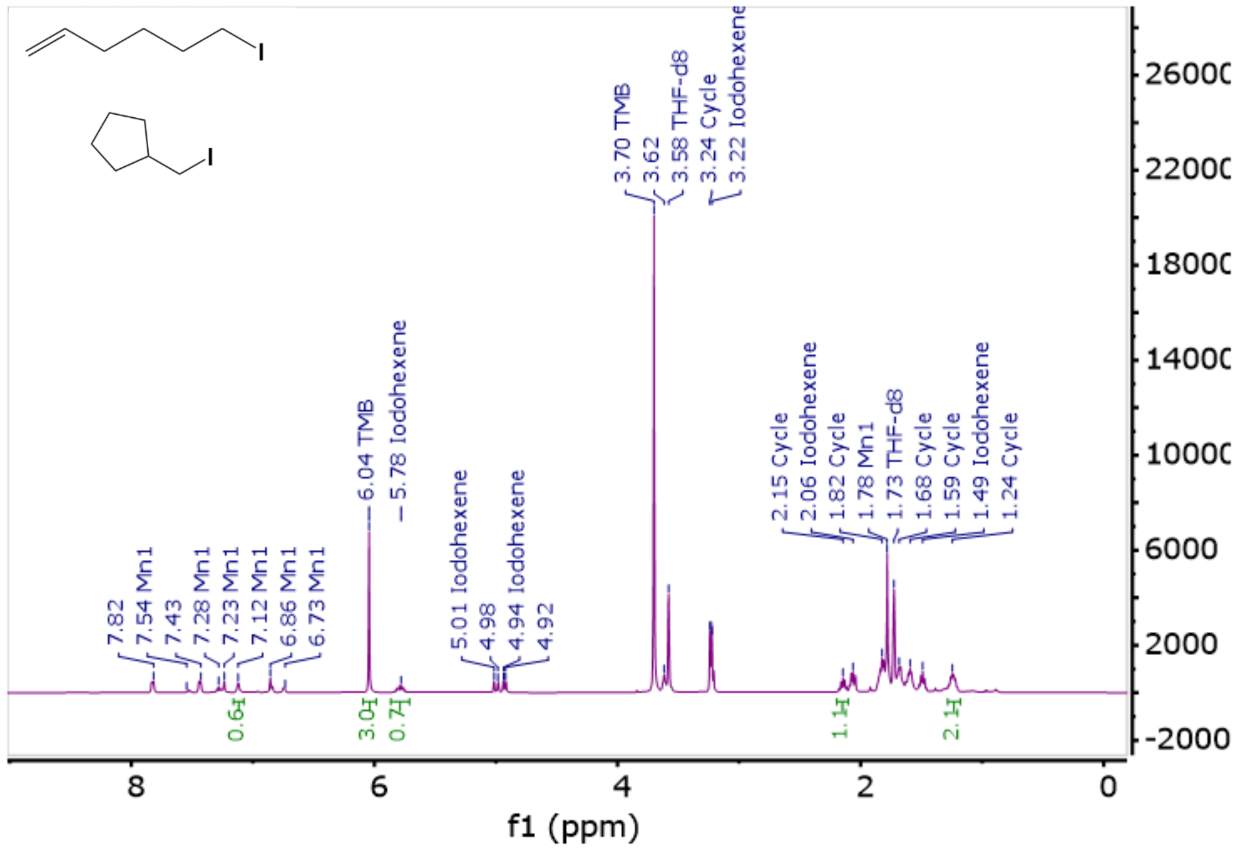

Fig S27 – Representative ^{1}H NMR spectrum of the reaction between **[2^{Ph}]**$^{-}$ and 6-iodo-1-hexene after 18 h (500 MHz, THF-d_8, 25°C). TMB = 1,3,5-trimethoxybenzene, Cycle = vinylcyclopentane.

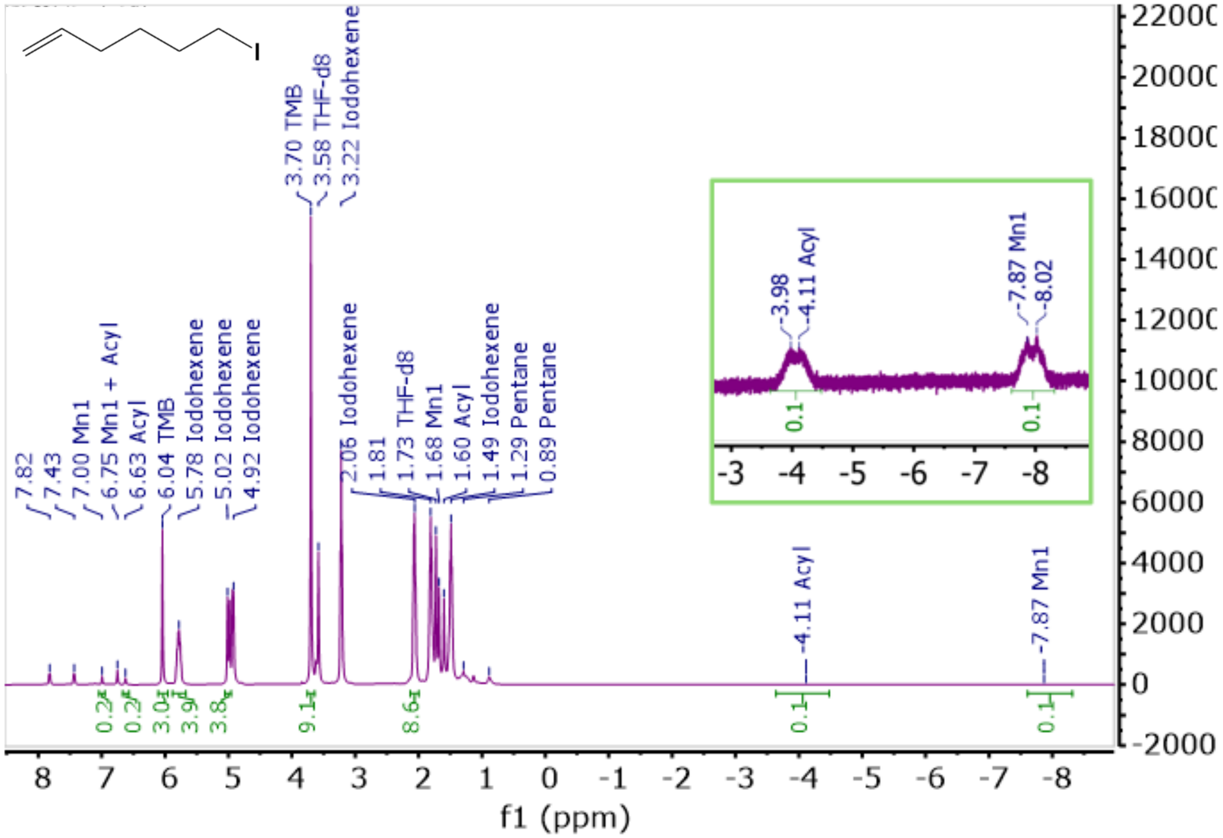

Fig S28 – Representative ^{1}H NMR spectrum of the reaction between **[2^{H}]**$^{-}$ and 6-iodo-1-hexene after 20 min, showing **1^{H}** and an acylated adduct similar to [Li(THF)$_2$][**1^{H}-Ac$_{eq}$**] (500 MHz, THF-d_8, 25°C).

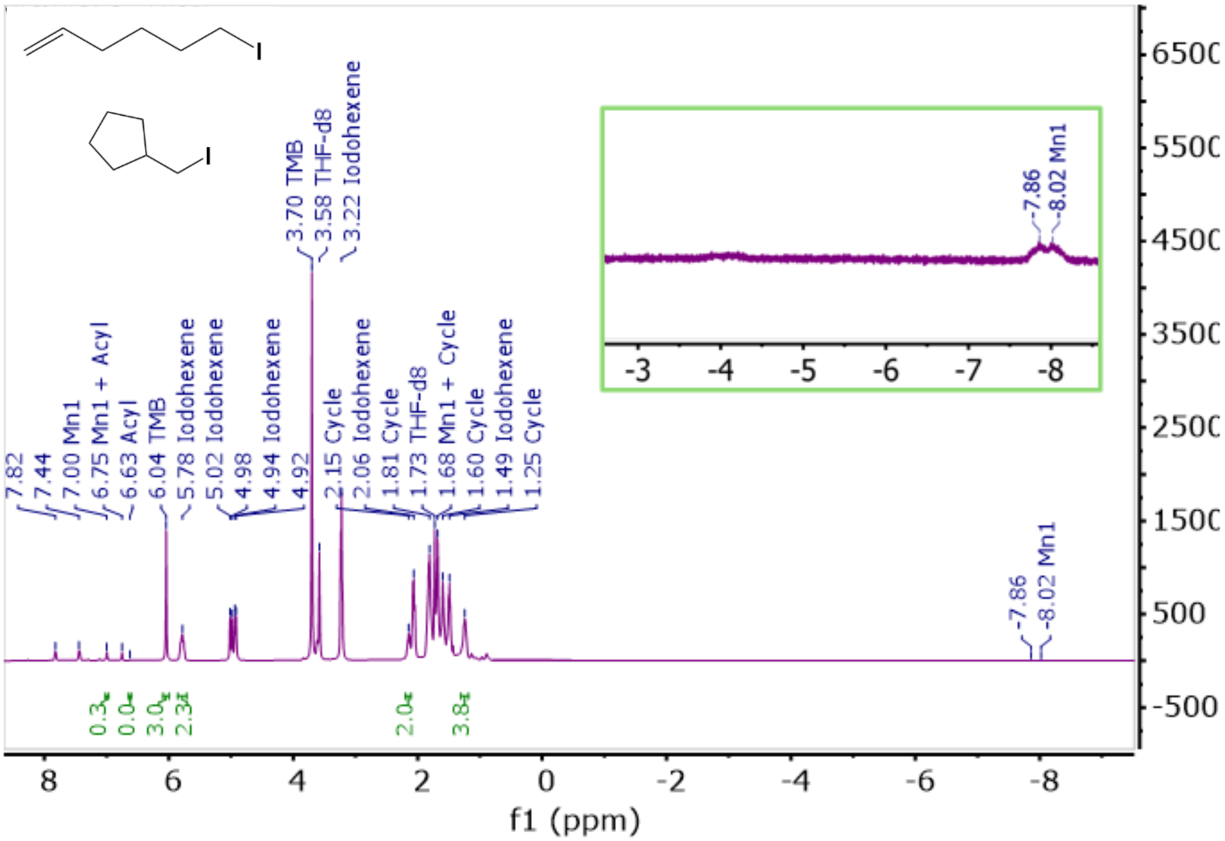

Fig S29 – Representative ^{1}H NMR spectrum of the reaction between **[2^H]^-** and 6-iodo-1-hexene after 18 h (500 MHz, THF-d_8, 25°C). TMB = 1,3,5-trimethoxybenzene, Cycle = vinylcyclopentane.

UV-Vis Spectra

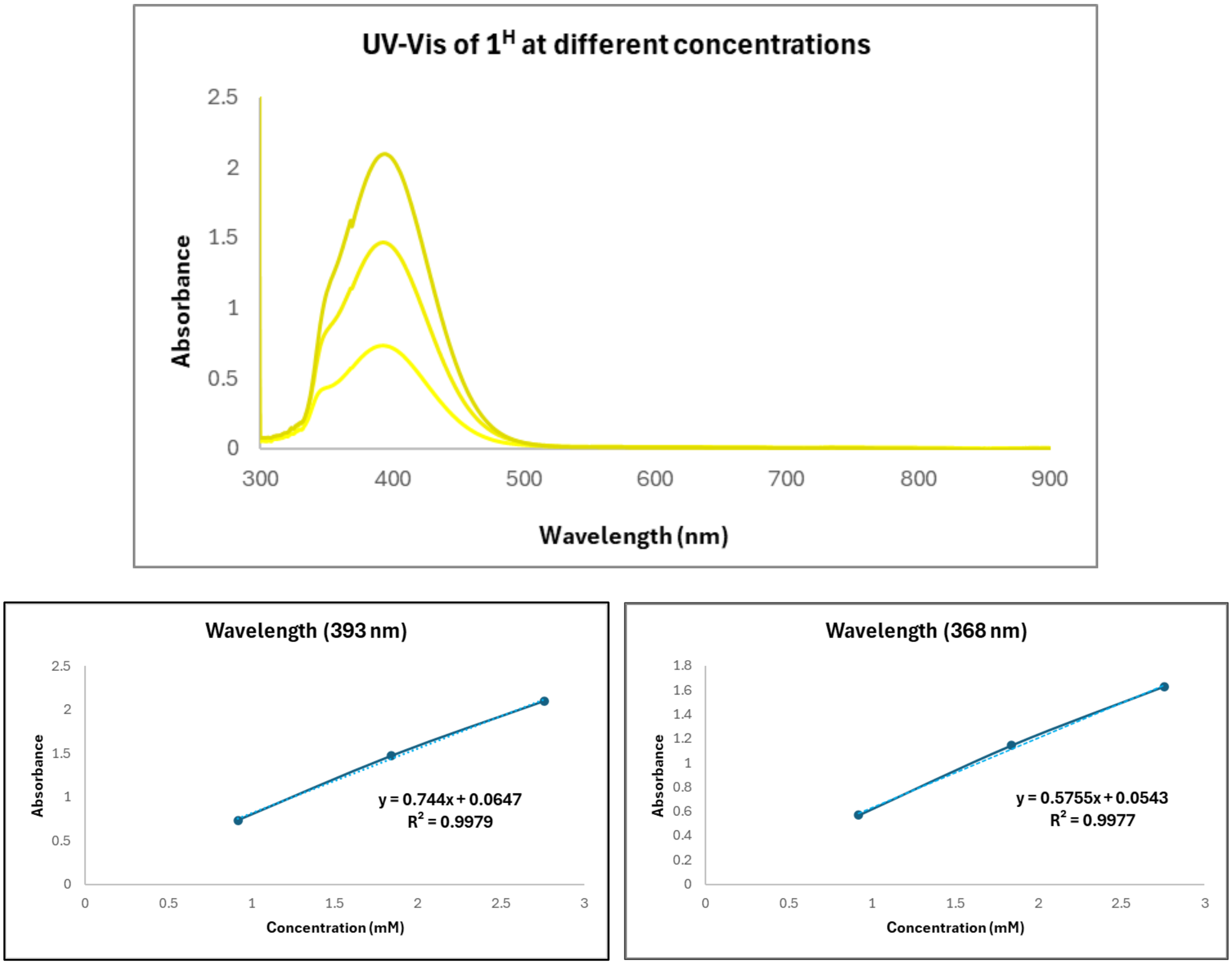

Figure S30: Top: Overlaid UV-visible spectra of **1^H** at different concentrations in THF. Bottom: Plot of **1^H** vs. absorbance at λ_{max} = 393 nm and λ_{max} = 368 nm.

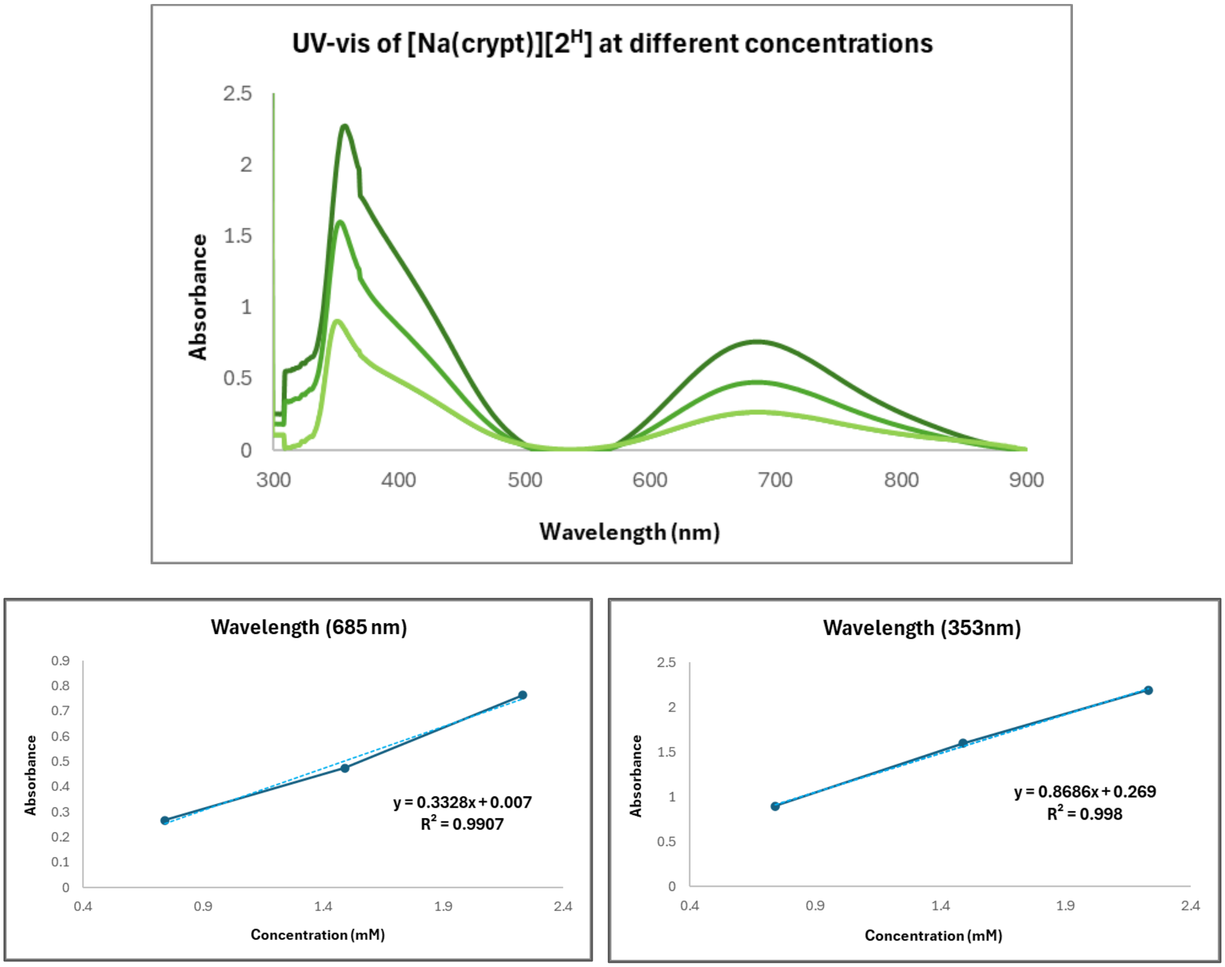

Figure S31: Top: Overlaid UV-visible spectra of [Na(crypt)][**2**^{H}] at different concentrations in THF. Bottom: Plot of [Na(crypt)][**2**^{H}] vs. absorbance at λ_{max} = 685 nm and λ_{max} = 353 nm.

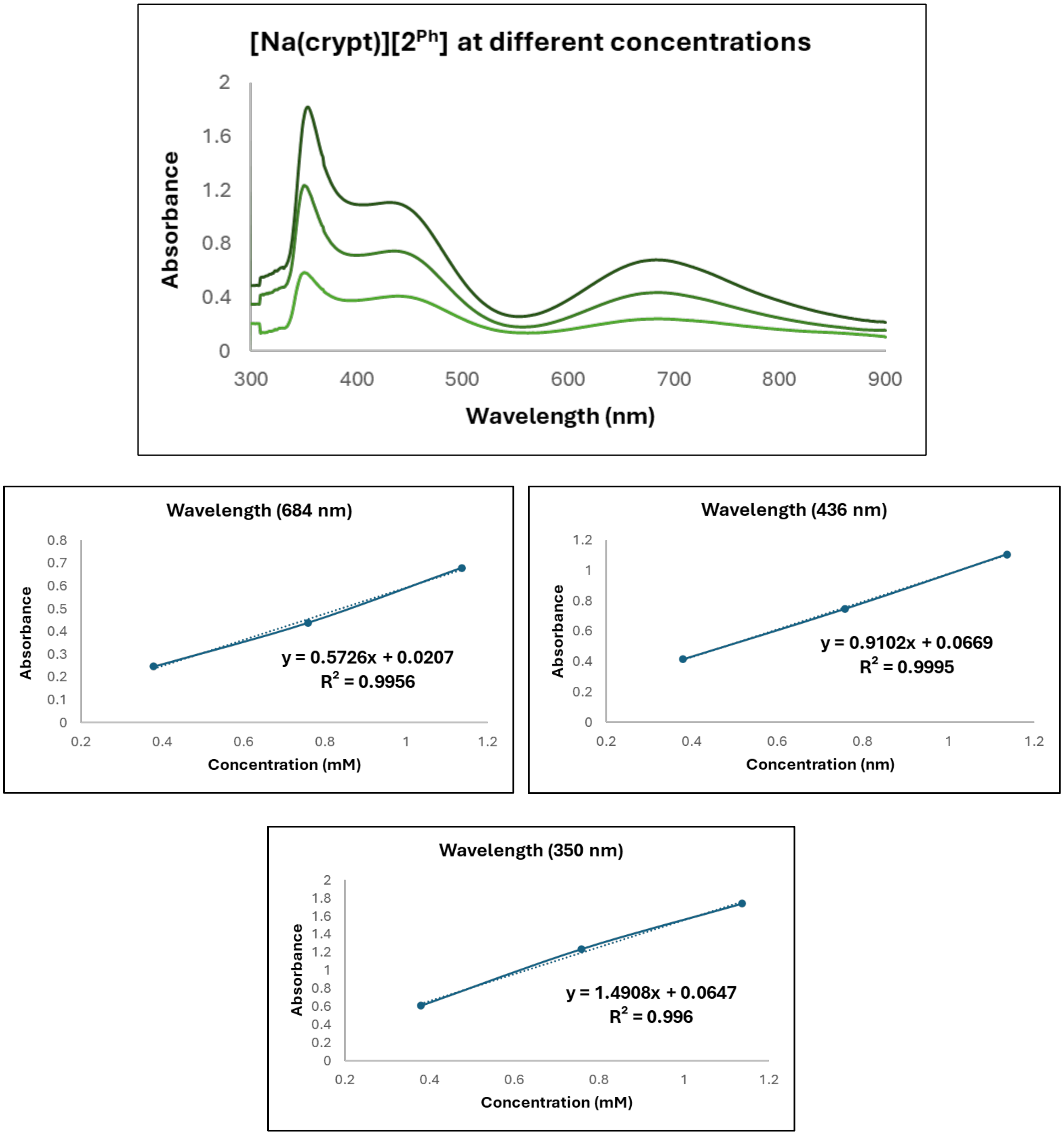

Figure S32: Top: Overlaid UV-visible spectra of [Na(crypt)][**2**^{Ph}] at different concentrations in THF. Bottom: Plots of [Na(crypt)][**2**^{Ph}] vs. absorbance at λ_{max} = 350 nm (THF, $\varepsilon_{\lambda max}$ ($M^{-1}cm^{-1}$): ε_{350} = 1500 ± 60), λ_{max} = 436 nm (THF, $\varepsilon_{\lambda max}$ ($M^{-1}cm^{-1}$): ε_{436} = 900 ± 60) and λ_{max} = 684 nm (THF, $\varepsilon_{\lambda max}$ ($M^{-1}cm^{-1}$): ε_{684} = 570 ± 20).

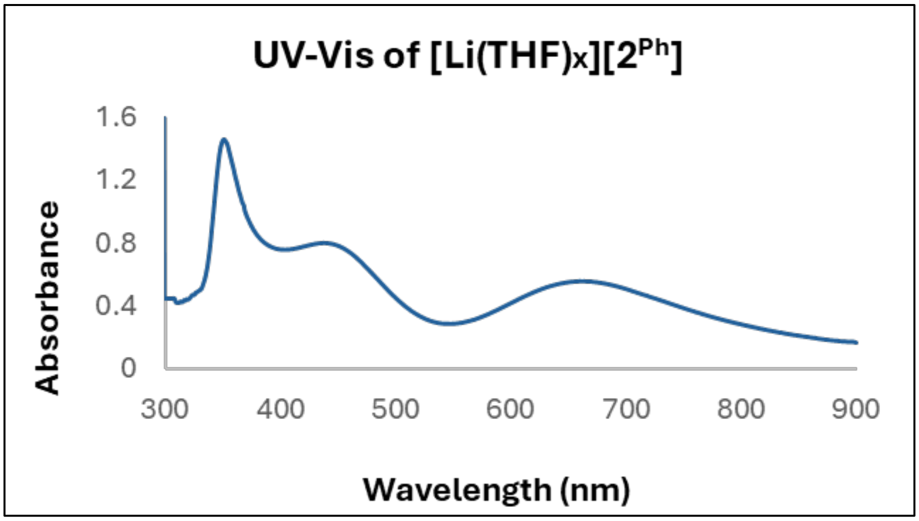

Figure S33: UV-visible spectrum of the 1e$^-$ reduction reaction of **1^{Ph}** to [**2^{Ph}**]$^-$ using *n*-BuLi in THF at 25°C. The absorbance at 662 nm (ε_{THF} = 570 M^{-1} cm^{-1}) was used to determine [Li(THF)$_x$][**2^{Ph}**] concentration and yield.

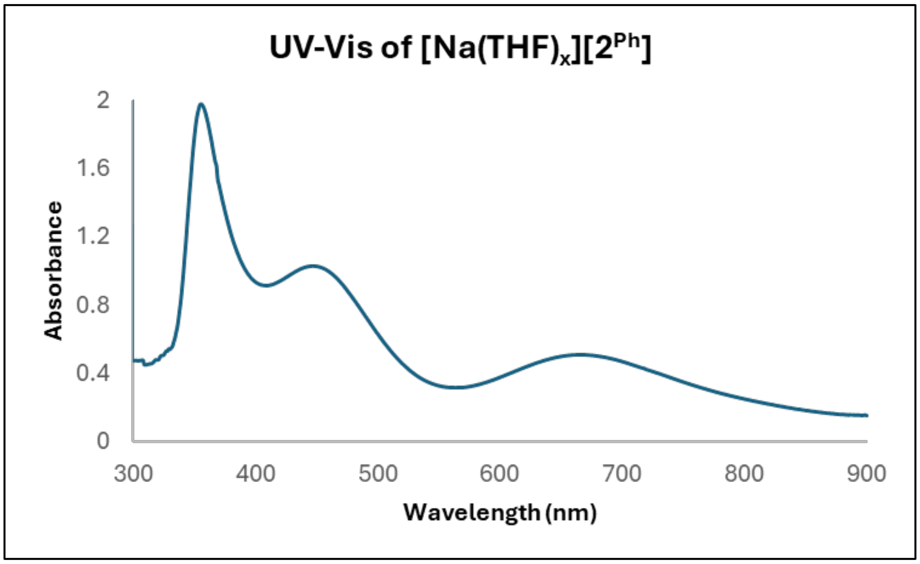

Figure S34: UV-visible spectrum of the 1e$^-$ oxidation reaction of [**3^{Ph}**]$^{2-}$ to [**2^{Ph}**]$^-$ using 1-bromobutane in THF at 25°C. The absorbance at 665 nm (ε_{THF} = 570 M^{-1} cm^{-1}) was used to determine [Na(THF)$_x$][**2^{Ph}**] concentration and yield.

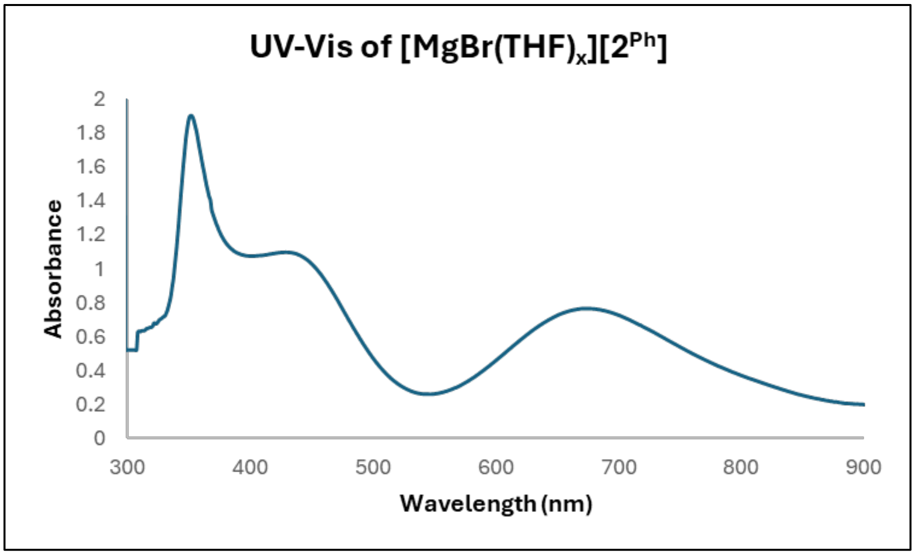

Figure S35: UV-visible spectrum of the 1e$^-$ reduction reaction of **1^{Ph}** to [**2^{Ph}**]$^-$ using allylMgBr in THF at 25°C. The absorbance at 669 nm (ε_{MeCN} = 200 M^{-1} cm^{-1}) was used to determine [MgBr(THF)$_x$][**2^{Ph}**] concentration and yield.

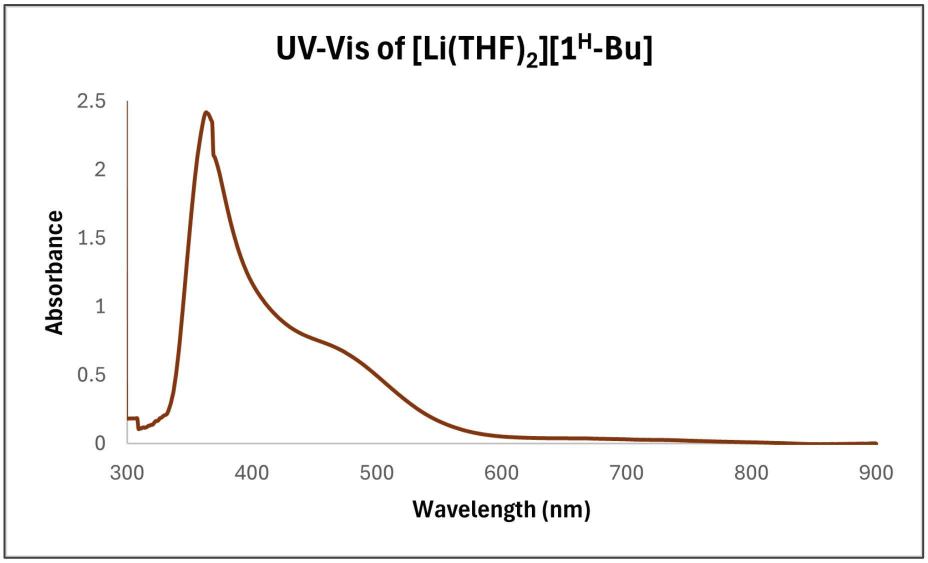

Figure S36: UV-visible spectrum of [Li(THF)$_2$][**1^H-Ac$_{eq}$**] in THF with absorbance λ_{max} = 363 nm and λ_{max} = 455 nm.

GC-MS

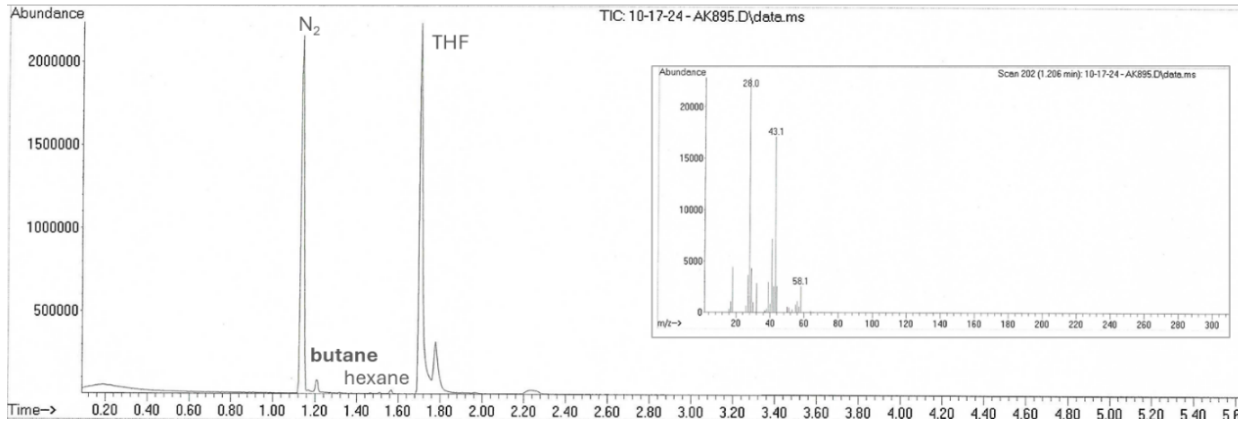

Figure S37: GC-MS results of the headspace gas from a reaction mixture of **1^{Ph}** and *n*-BuLi. The MS of butane (1.206 min) is included for clarity.

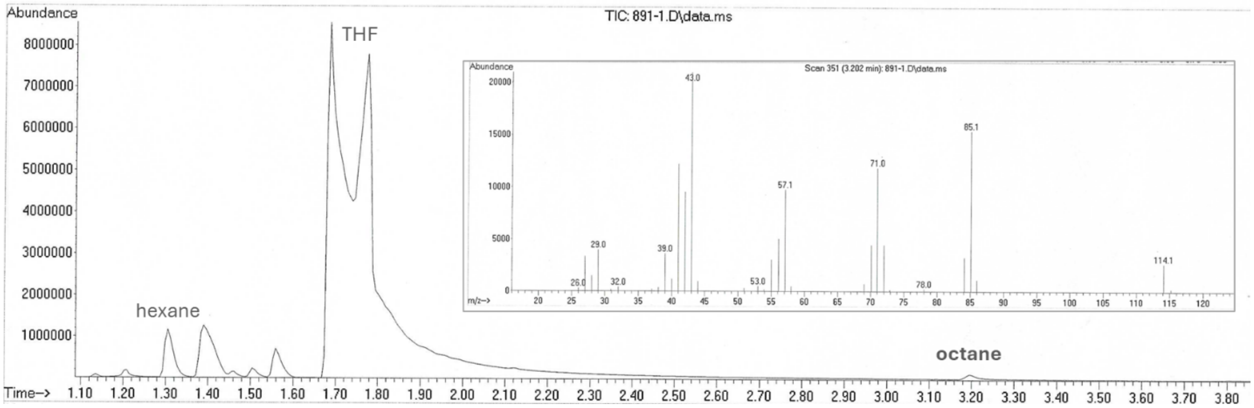

Figure S38: Representative GC-MS result from a reaction mixture of **1^{Ph}** and *n*-BuLi. The MS of octane (3.202 min) is included for clarity.

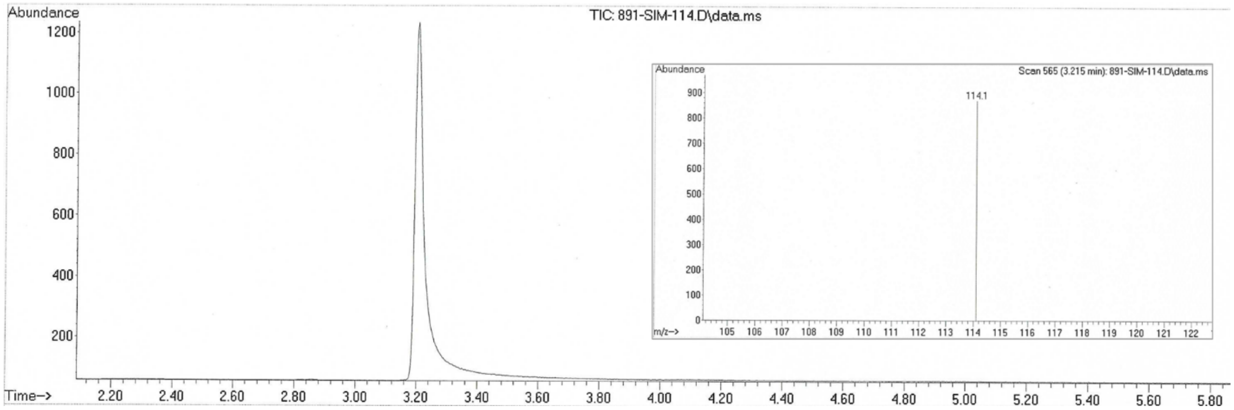

Figure S39: Representative SIM (Selected Ion Monitoring) GC-MS ion chromatogram of ion 114 (octane) from a reaction mixture of **1^{Ph}** and *n*-BuLi. The SIM MS of octane (3.221 min) is included for clarity.

References

(1) Williams, D. B. G.; Lawton, M. Drying of Organic Solvents: Quantitative Evaluation of the Efficiency of Several Desiccants. *J. Org. Chem.* **2010**, *75* (24), 8351–8354. https://doi.org/10.1021/jo101589h.
(2) Arrowsmith, M.; Hill, M. S.; Kociok-Köhn, G. Bis(Imidazolin-2-Ylidene-1-Yl)Borate Complexes of the Heavier Alkaline Earths: Synthesis and Studies of Catalytic Hydroamination. *Organometallics* **2009**, *28* (6), 1730–1738. https://doi.org/10.1021/om8010933.
(3) Nieto, I.; Bontchev, R. P.; Smith, J. M. Synthesis of a Bulky Bis(Carbene)Borate Ligand – Contrasting Structures of Homoleptic Nickel(II) Bis(Pyrazolyl)Borate and Bis(Carbene)Borate Complexes. *European Journal of Inorganic Chemistry* **2008**, *2008* (15), 2476–2480. https://doi.org/10.1002/ejic.200800034.
(4) Forshaw, A. P.; Bontchev, R. P.; Smith, J. M. Oxidation of the Tris(Carbene)Borate Complex PhB(MeIm)$_3$ Mn^{I} (CO)$_3$ to Mn^{IV} [PhB(MeIm)$_3$]$_2$ (OTf)$_2$. *Inorg. Chem.* **2007**, *46* (10), 3792–3794. https://doi.org/10.1021/ic070187w.
(5) Karagiannis, A.; Tyryshkin, A. M.; Lalancette, R. A.; Spasyuk, D. M.; Washington, A.; Prokopchuk, D. E. A Redox-Active Mn(0) Dicarbene Metalloradical. *Chem. Commun.* **2022**, *58* (93), 12963–12966. https://doi.org/10.1039/D2CC04677F.
(6) Marcó, A.; Compañó, R.; Rubio, R.; Casals, I. Assessment of Additives for Nitrogen, Carbon, Hydrogen and Sulfur Determination by Organic Elemental Analysis. *Microchim. Acta* **2003**, *142* (1), 13–19. https://doi.org/10.1007/s00604-002-0956-y.
(7) Karagiannis, A.; Tyryshkin, A. M.; Lalancette, R. A.; Spasyuk, D. M.; Washington, A.; Prokopchuk, D. E. A Redox-Active Mn(0) Dicarbene Metalloradical. *Chem. Commun.* **2022**, *58* (93), 12963–12966. https://doi.org/10.1039/D2CC04677F.
(8) Goddard, T. D.; Huang, C. C.; Meng, E. C.; Pettersen, E. F.; Couch, G. S.; Morris, J. H.; Ferrin, T. E. UCSF ChimeraX: Meeting Modern Challenges in Visualization and Analysis. *Protein Science* **2018**, *27* (1), 14–25. https://doi.org/10.1002/pro.3235.
(9) Meng, E. C.; Goddard, T. D.; Pettersen, E. F.; Couch, G. S.; Pearson, Z. J.; Morris, J. H.; Ferrin, T. E. UCSF ChimeraX : Tools for Structure Building and Analysis. *Protein Science* **2023**, *32* (11), e4792. https://doi.org/10.1002/pro.4792.
(10) Pettersen, E. F.; Goddard, T. D.; Huang, C. C.; Meng, E. C.; Couch, G. S.; Croll, T. I.; Morris, J. H.; Ferrin, T. E. UCSF ChimeraX : Structure Visualization for Researchers, Educators, and Developers. *Protein Science* **2021**, *30* (1), 70–82. https://doi.org/10.1002/pro.3943.
(11) Schaefer, A. J.; Ingman, V. M.; Wheeler, S. E. SEQCROW : A ChimeraX Bundle to Facilitate Quantum Chemical Applications to Complex Molecular Systems. *J Comput Chem* **2021**, *42* (24), 1750–1754. https://doi.org/10.1002/jcc.26700.
(12) Falivene, L.; Credendino, R.; Poater, A.; Petta, A.; Serra, L.; Oliva, R.; Scarano, V.; Cavallo, L. SambVca 2. A Web Tool for Analyzing Catalytic Pockets with Topographic Steric Maps. *Organometallics* **2016**, *35* (13), 2286–2293. https://doi.org/10.1021/acs.organomet.6b00371.
(13) Poater, A.; Cosenza, B.; Correa, A.; Giudice, S.; Ragone, F.; Scarano, V.; Cavallo, L. Samb *V* ca: A Web Application for the Calculation of the Buried Volume of N-Heterocyclic Carbene Ligands. *Eur J Inorg Chem* **2009**, *2009* (13), 1759–1766. https://doi.org/10.1002/ejic.200801160.
(14) Falivene, L.; Cao, Z.; Petta, A.; Serra, L.; Poater, A.; Oliva, R.; Scarano, V.; Cavallo, L. Towards the Online Computer-Aided Design of Catalytic Pockets. *Nat. Chem.* **2019**, *11* (10), 872–879. https://doi.org/10.1038/s41557-019-0319-5.
(15) Appel, A. M.; DuBois, D. L.; Rakowski DuBois, M. Molybdenum−Sulfur Dimers as Electrocatalysts for the Production of Hydrogen at Low Overpotentials. *J. Am. Chem. Soc.* **2005**, *127* (36), 12717–12726. https://doi.org/10.1021/ja054034o.
(16) Bao, D.; Millare, B.; Xia, W.; Steyer, B. G.; Gerasimenko, A. A.; Ferreira, A.; Contreras, A.; Vullev, V. I. Electrochemical Oxidation of Ferrocene: A Strong Dependence on the Concentration of the Supporting Electrolyte for Nonpolar Solvents. *J Phys Chem A* **2009**, *113* (7), 1259–1267. https://doi.org/10.1021/jp809105f.
(17) Karagiannis, A.; Neugebauer, H.; Lalancette, R. A.; Grimme, S.; Hansen, A.; Prokopchuk, D. E. Pushing the Limits of Organometallic Redox Chemistry with an Isolable Mn(−I) Dianion. *J. Am. Chem. Soc.* **2024**, *146* (28), 19279–19285. https://doi.org/10.1021/jacs.4c04561.

(18) Fulmer, G. R.; Miller, A. J. M.; Sherden, N. H.; Gottlieb, H. E.; Nudelman, A.; Stoltz, B. M.; Bercaw, J. E.; Goldberg, K. I. NMR Chemical Shifts of Trace Impurities: Common Laboratory Solvents, Organics, and Gases in Deuterated Solvents Relevant to the Organometallic Chemist. *Organometallics* **2010**, *29* (9), 2176–2179. https://doi.org/10.1021/om100106e.
(19) Golombek, A. P.; Hendrich, M. P. Quantitative Analysis of Dinuclear Manganese(II) EPR Spectra. *Journal of Magnetic Resonance* **2003**, *165* (1), 33–48. https://doi.org/10.1016/j.jmr.2003.07.001.

Part B

Computational

Contents

I. Statistical Measures **S41**

II. Computational Methods **S42**

III. Obtaining Linear Harmonic Frequency Scaling Factors for Composite-Level DFT Methods **S45**

IV. Assessing the Performance of Various Density Functional Approximations and Solvation Models **S48**

V. Further High-Energy Coordination Motifs of the Butyl-Adduct of 1^H **S50**

VI. Details on the Butyl Exchange Mechanism Intermediates via Analysis of the Dihedral Scan Along the Acyl Group in $[1\text{-Ac}_{eq}]^-$ **S52**

VII. Additional Computational Results **S54**

VII.I Potential Solvent Binding S55

VIII. List of Abbreviations **S57**

IX. References **S58**

I. Statistical Measures

The error of a calculated quantitity $q_{\text{calc.}}$ to the experimental reference value $q_{\text{exp.}}$ is given by

$$\Delta q = q_{\text{calc.}} - q_{\text{exp.}}\,. \tag{S1}$$

For a set of N errors $\{\Delta q_i\}$, common statistical measures are then defined by the following equations:

$$\text{Mean error: ME} = \frac{1}{N}\sum_{i=1}^{N}\Delta q_i \tag{S2}$$

$$\text{Mean absolute error: MAE} = \frac{1}{N}\sum_{i=1}^{N}|\Delta q_i| \tag{S3}$$

$$\text{Median error: MDN} = \begin{cases} \Delta q\left[\frac{N+1}{2}\right] & \text{if } N \text{ is odd} \\ \frac{1}{2}\cdot\left(\Delta q\left[\frac{N}{2}\right] + \Delta q\left[\frac{N}{2}+1\right]\right) & \text{if } N \text{ is even} \end{cases} \tag{S4}$$

$$\text{Root Mean Square Error: RMSE} = \sqrt{\frac{1}{N}\sum_{i=1}^{N}(\Delta q_i)^2} \tag{S5}$$

$$\text{Maximal error: MAX} = \max_i\left(\{\Delta q_i\}\right) \tag{S6}$$

$$\text{Minimal error: MIN} = \min_i\left(\{\Delta q_i\}\right) \tag{S7}$$

$$\text{Absolute maximal error: AMAX} = \max_i\left(\{|\Delta q_i|\}\right) \tag{S8}$$

$$\text{Standard deviation: SD} = \sqrt{\frac{1}{N-1}\sum_{i=1}^{N}(\Delta q_i - \text{ME})^2} \tag{S9}$$

II. Computational Methods

All final Gibbs energies were calculated employing

$$G \approx E_{\text{gas}}^{\text{SP}} + \Delta G_{\text{mRRHO}} + \Delta G_{\text{solv}}, \quad \text{(S10)}$$

where $E_{\text{gas}}^{\text{SP}}$ is the electronic energy in the gas phase, ΔG_{mRRHO} contributes thermostatistical corrections based on Grimme's mRRHO model[20], and ΔG_{solv} is the free energy of solvation.

Preoptimizations and Conformational Analysis

All geometries were initially preoptimized at the GFN1-xTB[21]/ALPB[22] (THF, $\varepsilon = 7.52$ [23], xTB version 6.7.0[24], `--opt extreme`, `--acc 0.01`) level of theory with initial guess geometries obtained from XRD data, if available. However, using both GFN1-xTB or GFN2-xTB[25] resulted in unrealistic rearrangements of the CO ligand shell of the dianionic complexes due to errors in the corresponding PESs. Thus, whenever GFN1-xTB/ALPB(THF) yielded unreasonable geometries, we opted for r^{2}SCAN-3c[26]/CPCM[27,28](THF) instead to still obtain reasonably preoptimized geometries. Nevertheless, these PESs errors limit the applicability of these methods, e.g., for conformational exploration. Fortunately, however, initial tests on the monoanionic acyl complexes (e.g., [1-Ac$_{\text{eq}}$]$^-$) showed that the conformational space is fairly limited, especially when considering the substantially varying relative energies of specific conformations such as required for the agostic intermediate (see Figure 7A, main text), thus leading us to expect only marginal errors for the lowest-energy conformers.

Hence, if not stated otherwise, we employed conformational sampling only when exploring different coordination modes of a specific complex as discussed in Section V. For this, we employed two separate approaches, CREST[29–31] (version 2.12, `--keepdir`, `--mrest 50`, `--mdlen x2.5`, `--opt extreme`) and GOAT[32] (`maxitermult 7.0`, `gfnuphill gfnff`, `freezeamides true`) at the GFN1-xTB/ALPB(THF) level of theory, respectively. To address issues such as spontaneous ligand dissociation during CREST simulations, as is generally observed for metal complexes[33,34], we constrained the first ligand shell, where the latter was determined by comparing each metal-to-atom bond length to the scaled sum (1.4) of Pyykkö's covalent radii[35] for all atom pairs in the complex. Harmonic bond constraints (`--cinp name.inp` → `force constant=0.15`, `--subrmsd`, `--tstep 2.5`) were then added to all bonds to the first ligand sphere atoms or additionally also for all (redundant) angles between the first ligand sphere atoms (e.g., $L^1{-}M{-}L^2$). Due to redundancy, the latter approach completely fixes the first ligand sphere, whereas the former prevents any ligand dissociation while still retaining the flexibility of rearranging to a different coordination motif in the conformational run. Hence, we ultimately combined the generated conformers from maximal four conformational runs (the unconstrained, bond-constrained, and angle-constrained CREST runs,

and those from an isolated GOAT run). The resulting CEs were then preliminarily sorted with CREST (`--cregen conformers.xyz`, `--notopo`) and energetically reranked via part one of version 1.2.0. of the CENSO program[36], using r^2SCAN-3c[26]/SMD[37](THF) + G_{mRRHO}-SPH[38][GFN1-xTB[21]/ALPB(THF)] as the level of theory. Please note that ORCA[39,40] 6.0.1 was used for any high-level quantum chemical calculations throughout this study. These calculations were also carried out in an unrestricted fashion to allow for potential orbital instabilities.[41]

Final Geometries and Harmonic Frequencies

Final geometries were obtained with B3LYP-3c[42]/CPCM[27,28](THF) (`B3LYP`, `D3BJ`, `def2-mSVP`, `gCP(DFT/SV(P))`, `ABC`, `TIGHTOPT`), which was also used to validate the nature of the calculated stationary points of each PES (no/only one imaginary eigenmode for minima/transition states). Every computed harmonic frequency has been linearly scaled with frequency-range dependent scaling factors as determined in Section III. based on work from McKemmish and co-workers.[43,44] B3LYP-3c was initially chosen as it has previously been successfully employed to reproduce experimental IR spectroscopical data with excellent agreement at reduced computational cost.[42] However, in retrospect, we conclude that r^2SCAN-3c[26] would have been equally suited for accurately reproducing the experimental CO bands, while also circumventing issues with locating some of the key structures for the exchange mechanism as described in the main text (Figure 7), which will be relevant for future studies tackling the catalytic abilities of the investigated manganese complexes.

Some transition states were obtained by employing the NEB method.[45,46] Besides featuring only one imaginary eigenmode, all transition states were validated by visual inspection of the imaginary eigenmode and via bidirectional IRC[47,48] calculations (`maxiter 30`, `de_init_displ 3.0`) whose endpoints were fully optimized to minimum geometries with the above level of theory. If the latter converged to chemically questionable minimum structures, manual dislocation of the transition state geometries along the corresponding imaginary eigenmode (± 1.0) followed by a simple geometry optimization yielded the expected minimum structures.

More levels of theories were employed for obtaining new scaling factors for composite DFT methods from the Grimme group (refer to Section III. for details).

Electronic Energy Refinements

All final electronic energies were refined at the PW6B95[49]-D4/def2-QZVPPD[50,51] level of theory with `TIGHTSCF`, `DEFGRID3`, `RIJCOSX`[52,53], and with a large def2/JK[54] auxiliary basis set. To break the spin symmetry of the initial SCF guess for potential BS states, high-spin (HS) ($\Delta M = +2$) orbitals were calculated first and subsequently converged to the corresponding low-spin (LS) orbitals (e.g., triplet start orbitals for a singlet SCF calculation). The stability of

the LS orbitals was verified via analysis of the lowest eigenvalues of the orbital Hessian.[55–57] If SCF convergence turned out to be challenging, Fermi smearing was employed with an increasing electronic temperature until convergence was reached. This was followed by re-convergence at 0 K using the previous orbitals for a new density guess to allow for comparable electronic energies.

Other DFAs tested with the def2-QZVPPD basis set in the redox potential benchmark (see Section IV.) are shown in Table S3.

Table S3. DFAs with corresponding references used in the small redox potential benchmark herein (Section IV.).

Functional	References
r^2SCANh-D4	[58–60]
TPSSh-D4	[59–61]
B3LYP-D4	[59,60,62,63]
r^2SCAN0-D4	[58–60]
PBE0-D4	[59,60,64]
PW6B95-D4	[49,59,60]
ωB97X-V	[65,66]
ωB97X-D4rev	[59,60,67]
ωB97M-V	[66,68]
ωB97M-D4rev	[59,60,69]

Solvation Free Energies

ΔG_{solv} was obtained via two individual single-point energy evaluations, one with (solution phase) and one without (gas phase) application of an implicit solvation model. Four continuum models were evaluated for THF and MeCN (see Section IV.), namely COSMO-RS[70–72], COSMO-RS[70–72](fine), CPCM[27,28], and SMD[37]. For the latter two, r^2SCAN-3c densities were used for cavity construction within ORCA (`TIGHTSCF`, `DEFGRID3`), whereas COSMO-RS solvation energies were provided by version C3.0 (release 16.01, 2016, parameterization: BP_TZVP_C30_1601.ctd and BP_TZVPD_FINE_C30_1601.ctd, default Gsolv option) of the COSMOtherm program, utilizing version 7.7.1 of the Turbomole[73] program suite. All solvation energies were standard state corrected to 1 mol L^{-1}.[74]

Further Computational Details

The diffusion coefficients of the oxidized and reduced species were assumed to be identical for the calculation of the experimentally measured potentials[75] and the Fc/Fc^+ redox couple

was chosen for internal referencing of all redox potentials, using the "direct" approach (Figure S40).[76,77] ChimeraX[78,79] (version 1.9) was used for visualization purposes.

$$\mathrm{Fc\ (solv)} \rightleftharpoons \mathrm{Fc^{+}\ (solv) + e^{-}\ (solv)} \qquad -\Delta G_{\mathrm{ref}} = F \cdot E^{o}_{\mathrm{abs}}(\mathrm{Fc/Fc^{+}})$$

$$\mathbf{R}\ \mathrm{(solv) + e^{-}\ (solv)} \rightleftharpoons \mathbf{R}^{\boldsymbol{\cdot}\mathbf{-}}\ \mathrm{(solv)} \qquad \Delta G_{\mathrm{R}} = - F \cdot E^{o}_{\mathrm{abs}}(\mathbf{R}^{\boldsymbol{\cdot}\mathbf{-}}/\mathbf{R})$$

$$\mathbf{R}\ \mathrm{(solv) + Fc\ (solv)} \rightleftharpoons \mathbf{R}^{\boldsymbol{\cdot}\mathbf{-}}\ \mathrm{(solv) + Fc^{+}\ (solv)} \qquad \Delta G_{\mathrm{DFT}} = - F \cdot E^{o}_{\mathrm{vs.\ Fc/Fc+}}(\mathbf{R}^{\boldsymbol{\cdot}\mathbf{-}}/\mathbf{R}) = - F \cdot (E^{o}_{\mathrm{abs}}(\mathbf{R}^{\boldsymbol{\cdot}\mathbf{-}}/\mathbf{R}) - E^{o}_{\mathrm{abs}}(\mathrm{Fc/Fc^{+}}))$$

Figure S40. General reaction scheme used for calculating the computational redox potentials, where F is Faraday's constant.

III. Obtaining Linear Harmonic Frequency Scaling Factors for Composite-Level DFT Methods

We followed a previously established procedure by McKemmish and co-workers[44] that relies on the VIBFREQ1295 dataset[43] for obtaining the frequency-range-dependent linear scaling factors from Table S5. For this, we re-implemented the procedure as outlined in references [44] and [43] in Python to be used in combination with ORCA (`DEFGRID3`, `VERYTIGHT`, `VERYTIGHTOPT`, `NOSYM`) rather than Gaussian. The script and an exemplary ORCA input can be found in the Supporting Information .zip file. Further changes to the VIBFREQ1295 procedure involve the number of test and training data splits, which we increased from 100 to 1000. Transition state structures with imaginary modes were removed if no convergence to a true minimum structure could be established. Similarly, molecules with excessively deviating frequencies compared to experiment (errors greater than 1500 cm^{-1}, CH radical with PBEh-3c) or calculations showing large spin polarization (observed for HF-3c) were equally neglected for the calculation of scaling factors. Table S4 provides the difference in scaling factors for three DFAs when using our re-implementation compared to the previously reported scaling factors by McKemmish *et.al.*

Table S4. Scaling factors resulting from analysis of the VIBFREQ1295[43] dataset when employing the DFAs B3LYP[62,63]-D3(BJ)[80,81], TPSS0[82]-D3(BJ)[80,81], and PBE0[64]-D3(BJ)[80,81] with the def2-TZVP[51] basis set in the gas phase. Frequency ranges correspond to global (all frequencies), low (< 1,000 cm^{-1}), mid (1,000-2,000 cm^{-1}), and high (> 2,000 cm^{-1}). "calc" indicates the scaling factors obtained with our approach compared to the published reference values ("ref").[44]

Range	Level of Theory	λ_{calc}	λ_{ref}
Global	B3LYP-D3(BJ)	0.9686	0.9670
Low	B3LYP-D3(BJ)	0.9957	0.9914
Mid	B3LYP-D3(BJ)	0.9760	0.9749
High	B3LYP-D3(BJ)	0.9641	0.9626
Global	TPSS0-D3(BJ)	0.9518	0.9509
Low	TPSS0-D3(BJ)	0.9710	0.9700
Mid	TPSS0-D3(BJ)	0.9569	0.9569
High	TPSS0-D3(BJ)	0.9488	0.9476
Global	PBE0-D3(BJ)	0.9600	0.9592
Low	PBE0-D3(BJ)	0.9793	0.9755
Mid	PBE0-D3(BJ)	0.9692	0.9681
High	PBE0-D3(BJ)	0.9558	0.9553

Minor deviations in the reproduced scaling factors are to be expected given the change in quantum chemical software to ORCA. Nevertheless, very similar scaling factors were obtained using our approach, which validates our re-implementation. Further, the remaining differences in scaling factors can be safely neglected given the inherently empirical nature of applying scaling factors to harmonic frequencies to implicitly correct for anharmonicities in the PESs, which necessarily also limits the generally expected accuracy to similar wavenumber regimes. Finally, Table S5 shows the resulting scaling factors for the most well-known composite methods from the Grimme group.

Table S5. Scaling factors of the most important composite methods from the Grimme group (HF-3c[83], B97-3c[84], r^2SCAN-3c[26], B3LYP-3c[42], PBEh-3c[85], and ωB97X-3c[67]), resulting from the application of the slightly modified procedure from McKemmish and co-workers as outlined above.[44] Frequency ranges correspond to global (all frequencies), low (< 1,000 cm^{-1}), mid (1,000-2,000 cm^{-1}), and high (> 2,000 cm^{-1}).

Range	Method	λ
Global	HF-3c	0.8427
Low	HF-3c	0.8711
Mid	HF-3c	0.8641
High	HF-3c	0.8348
Global	B97-3c	0.9808
Low	B97-3c	1.0204
Mid	B97-3c	0.9827
High	B97-3c	0.9768
Global	r^2SCAN-3c	0.9704
Low	r^2SCAN-3c	1.0039
Mid	r^2SCAN-3c	0.9805
High	r^2SCAN-3c	0.9646
Global	B3LYP-3c	0.9731
Low	B3LYP-3c	0.9992
Mid	B3LYP-3c	0.9672
High	B3LYP-3c	0.9727
Global	PBEh-3c	0.9331
Low	PBEh-3c	0.9468
Mid	PBEh-3c	0.9311
High	PBEh-3c	0.9326
Global	ωB97X-3c	0.9375
Low	ωB97X-3c	0.9618
Mid	ωB97X-3c	0.9481
High	ωB97X-3c	0.9325

IV. Assessing the Performance of Various Density Functional Approximations and Solvation Models

Albeit PW6B95-D4/def2-QZVPP/COSMO-RS has already been successfully applied as the level of theory for final energy refinements in a previous study by some of us[86], we systematically revisited the validation of the experimental redox potentials shown in Table S6 by assessing the combination of 10 DFAs with four different implicit solvation models and the def2-QZVPPD basis set, arriving at Table S7 with the final statistics for the best-performing method combinations for this small redox potential benchmark. Please note that all MeCN potentials have been calculated with structures properly relaxed in MeCN continuum solvent. All calculated data are provided as .csv files in the main .zip file that is part of this Supporting Information.

Table S6. Experimental redox potential data in V vs. Fc/Fc$^+$ for the $Mn^{I/0}$ and $Mn^{0/-I}$ redox couples in THF and MeCN solvent as previously measured for the complexes with phenyl-substituents at boron (-**Ph**)[86] and as reported herein for the analogous complexes with hydrogen substituents at boron (-**H**). The corresponding DFT-computed values at the PW6B95-D4/def2-QZVPPD/COSMO-RS level of theory are shown in parentheses.

Complex	Solvent	Redox Potential	Solvent	Redox Potential
$Mn^{I/0}$-**Ph**	MeCN	-1.76 (-1.81)	THF	-1.96 (-1.89)
$Mn^{0/-I}$-**Ph**	MeCN	-2.85 (-3.09)	THF	-3.13 (-3.31)
$Mn^{I/0}$-**H**	MeCN	-1.96 (-1.93)	THF	-2.02 (-2.06)
$Mn^{0/-I}$-**H**	MeCN	-2.90 (-2.97)	THF	-2.85 (-3.22)

Table S7. Statistical measures for a few method combinations with the lowest observed MAEs for evaluating **all eight** experimental redox potentials from Table S6. All statistical measures are provided in units of V vs. Fc/Fc$^+$.

DFA	Solvation Model	ME	MAE	MDN	RMSE	MAX	MIN	AMAX	SD	N
PW6B95-D4	COSMO-RS	-0.11	0.13	-0.06	0.17	0.07	-0.37	0.37	0.15	8
PBE0-D4	COSMO-RS	0.09	0.20	0.13	0.22	0.33	-0.25	0.33	0.22	8
PW6B95-D4	CPCM	-0.09	0.23	-0.01	0.28	0.25	-0.57	0.57	0.28	8
PW6B95-D4	SMD	-0.06	0.26	-0.00	0.30	0.34	-0.58	0.58	0.32	8
PBE0-D4	CPCM	0.11	0.27	0.20	0.30	0.37	-0.46	0.46	0.30	8
PBE0-D4	COSMO-RS(fine)	-0.15	0.27	-0.04	0.41	0.27	-0.93	0.93	0.41	8
r^2SCAN0-D4	COSMO-RS	0.16	0.29	0.18	0.35	0.50	-0.35	0.50	0.33	8
PBE0-D4	SMD	0.14	0.31	0.26	0.34	0.46	-0.46	0.46	0.33	8

Table S8. Statistical measures for a few method combinations with the lowest observed MAEs for evaluating **only the four $Mn^{0/-I}$** redox potentials. All statistical measures are provided in units of V vs. Fc/Fc^{+}.

DFA	Solvation Model	ME	MAE	MDN	RMSE	MAX	MIN	AMAX	SD	N
PW6B95-D4	COSMO-RS	0.00	0.05	-0.01	0.05	0.07	-0.05	0.07	0.06	4
ωB97X-V	COSMO-RS	-0.08	0.08	-0.09	0.09	-0.00	-0.13	0.13	0.06	4
ωB97X-D4rev	COSMO-RS	-0.08	0.08	-0.07	0.09	-0.03	-0.15	0.15	0.06	4
ωB97M-D4rev	COSMO-RS	-0.09	0.09	-0.09	0.11	-0.04	-0.15	0.15	0.06	4
ωB97M-V	COSMO-RS	-0.11	0.11	-0.12	0.12	-0.03	-0.16	0.16	0.06	4
TPSSh-D4	COSMO-RS(fine)	0.02	0.11	0.03	0.12	0.17	-0.14	0.17	0.14	4
PBE0-D4	COSMO-RS(fine)	0.11	0.14	0.11	0.16	0.27	-0.05	0.27	0.14	4
PW6B95-D4	CPCM	-0.05	0.14	-0.01	0.16	0.09	-0.27	0.27	0.17	4

Table S7 clearly suggests PW6B95-D4/def2-QZVPPD/COSMO-RS as the most suitable combination of DFA and solvation model to reproduce the electro-thermochemistry of the investigated manganese complexes herein. However, while achieving, on average, good agreement with experiments, the large AMAX values across all method combinations are initially concerning and warrant further discussion. The $Mn^{0/-I}$ redox potentials (see Table S8) can be reproduced with excellent agreement to the experimental values, further indicating issues with the dianionic complexes as was previously observed for the large PES errors at semiempirical quantum mechanical level of theory (see Section II., preoptimization).

Additionally, we observed that most of the tested DFAs yielded severely spin-symmetry broken SCF solutions with deviations in $\langle \hat{S}^2 \rangle$ (from the expected singlet value of 0.0) that were greater than 1.0. All of the above results suggest electronic difficulties of our DFT-based description of the dianions[87], which can be rationalized based on the d^8 electron configuration of a Mn(-I) center in an octahedral ligand field with thus one electron in both the d_{z^2} and $d_{x^2-y^2}$ orbital. For a total spin singlet, the latter occupation can be considered as the antiferromagnetically coupled case of the two-electron in two-orbitals problem, as, for instance, described in Section 7.10 of reference [88], and can thus not be captured within a single-reference electronic structure approach as DFT. Please note that using Yamaguchi's spin-projection scheme[89,90] also did not systematically improve the accuracy of any of these results, which is why we adhere to the **non** spin-projected values reported above and summarized in Table S6.

Despite these inherent difficulties with the dianionic complexes, all key computational results reported in the main text primarily focus on the $Mn^{I/0}$-complexes, for which this is not a problem. Hence, our DFT workflow is well-suited to tackle the investigated manganese chemistries, while good agreement with experiments may be expected (Table S8).

V. Further High-Energy Coordination Motifs of the Butyl-Adduct of 1^{H}

To explore other coordination motifs of the acyl or butyl complexes $[1\text{-Ac}]^{-}$ and $[1\text{-Bu}]^{-}$, respectively, we performed CREST and GOAT simulations on a number of different initial coordination environments. Evidently, the lowest-energy complex, e.g., $[1\text{-Ac}_{eq}]^{-}$ for the acyl-bound species, is obtained without the use of proper constraints, which is why bond and angle constraints (see Section II. for definitions and overall conformational procedure) were introduced. Sometimes, only angle-constrained conformational runs were successful for obtaining certain high-energy orientations; therefore, a varying number of (angle- and/or bond-constrained) CREST and GOAT runs were employed, subsequently combined, and finally reranked for each species. Furthermore, some motifs ultimately dissociated or rearranged in the final unconstrained geometry optimizations with B3LYP-3c/CPCM(THF). Hence, only true local minima are shown in the summary in Figure S41, in which some of the key structures from Figure 7A of the main text also appear. The remaining structures from Figure 7A were obtained via a fine dihedral scan as discussed in Section VI.

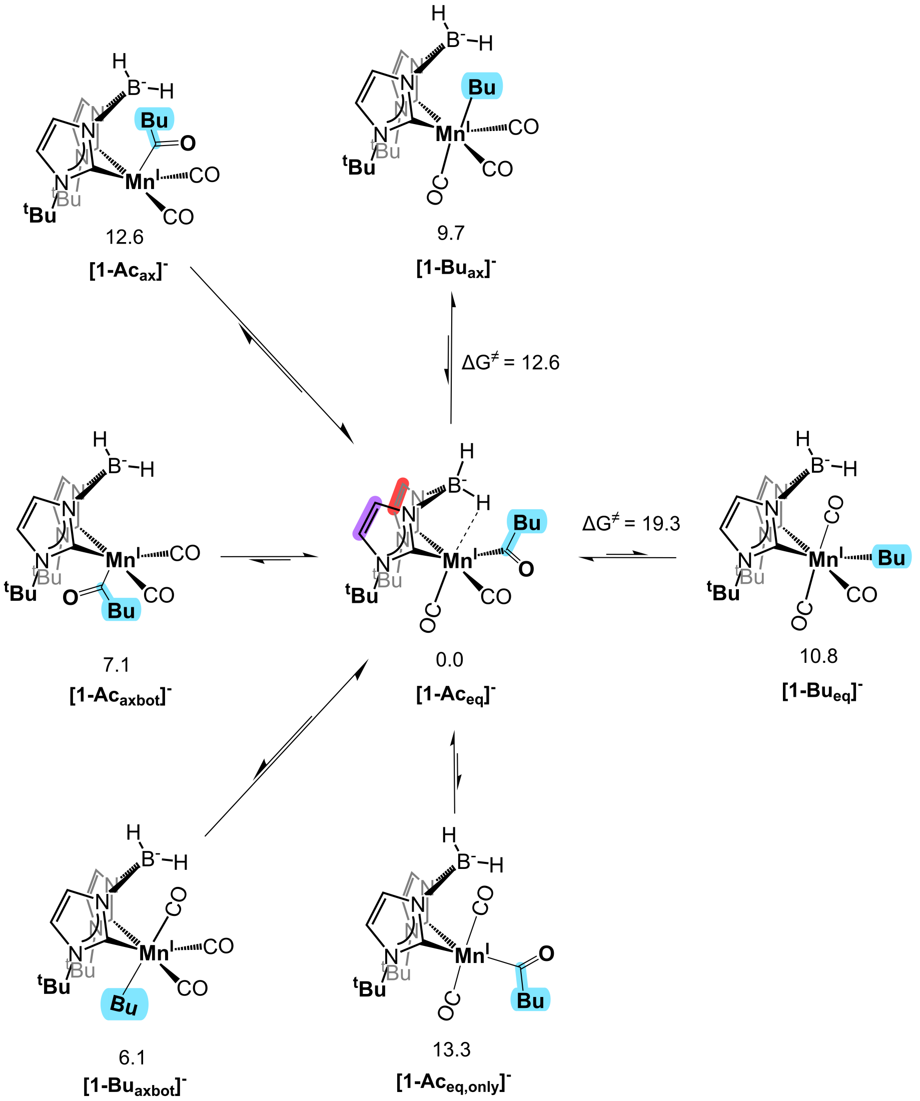

Figure S41. Overview of all (high-energy) coordination motifs obtained for the butyl adducts of 1^{H} with their relative free energy at the PW6B95-D4/def2-QZVPPD/COSMO-RS(THF)//B3LYP-3c/CPCM(THF) level of theory in kcal mol^{-1}. Two fully conformationally sampled transition-state barrier heights for specific transformations are also provided in kcal mol^{-1}. Notation as used in the main text, with the nature of ^{n}Bu^{-} binding denoted as ^{n}Bu (Bu) or acyl (Ac): axial-bottom (axbot), equatorial (eq).

VI. Details on the Butyl Exchange Mechanism Intermediates via Analysis of the Dihedral Scan Along the Acyl Group in [1-Ac$_{eq}$]$^-$

Figure S42 shows a full 360° dihedral scan along the carbonyl group of the equatorial acyl ligand in [1-Ac$_{eq}$]$^-$, featuring distinct local minima and maxima, corresponding to minimum and transition state structures that when fully optimized are given as the structures shown in Figure S42b. Interestingly, formation of the acyl complex [1-Ac$_{eq,CO}$]$^-$ (**A**) with accompanying cleavage of the agostic Mn−H interaction can be observed as the maximum near **B**, which ultimately is associated with a barrier height of 6.9 kcal mol^{-1} when converged to a true transition state (also used in Figure 7A of the main text). From the acyl complex **A**, rotation along the dihedral angle in both directions leads to the transition states **C** and **E**, respectively, which connect two different conformers with similar total free energy, featuring a carbonyl group of the acyl ligand that is either oriented above (**F**) or below (**D**) the equatorial plane. Reaching the acyl transition state **B** or changing from one conformer to another is evidently (thermally) facile via both **F** or **D**. However, we chose the one with the lower Gibbs free energy and thus report values of 4.5 kcal mol^{-1} and 6.9 kcal mol^{-1} for **E** and **B** in the main text, respectively.

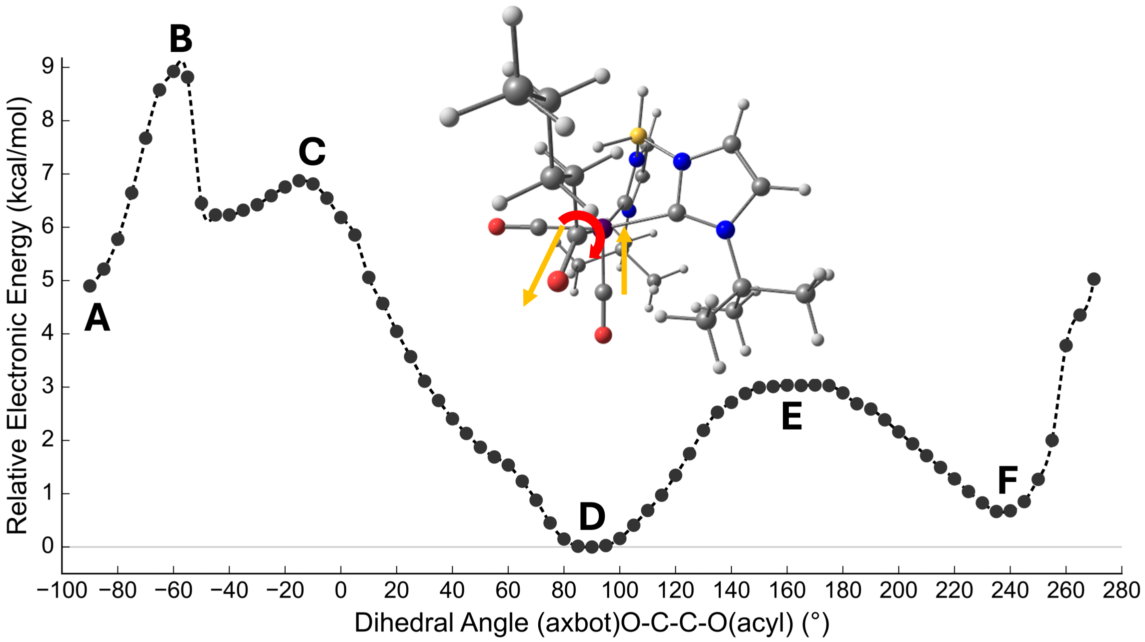

(a)

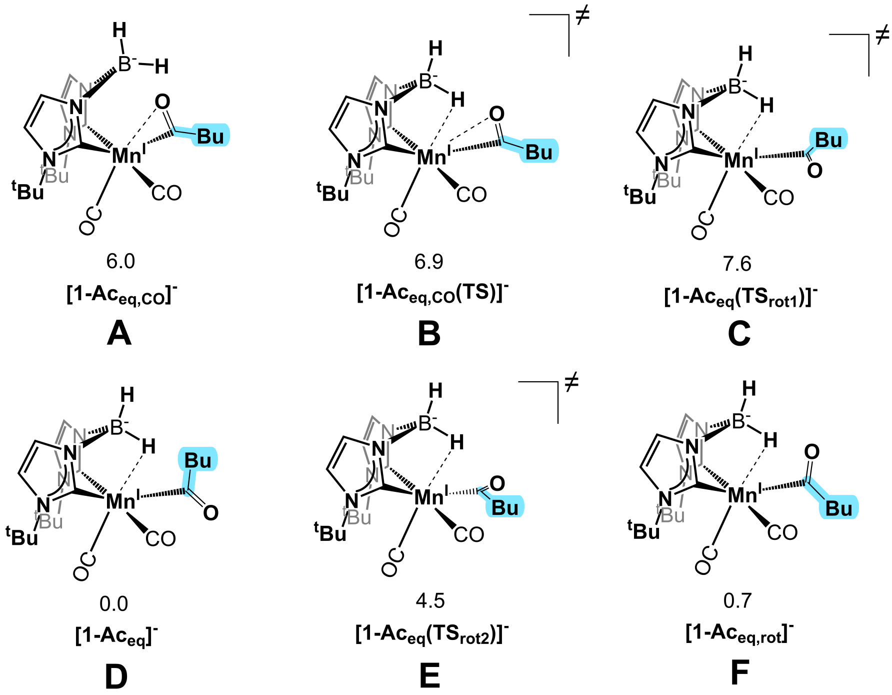

(b)

Figure S42. A: Dihedral scan along the dihedral angle between the axial CO ligand and the carbonyl group of the equatorial acyl ligand. The exact dihedral angle used for this scan is highlighted by the red and orange arrows in a representative molecular structure inlay. The constrained geometries were obtained at the B3LYP-3c/CPCM(THF) level of theory, whereas the provided relative energies are at the PW6B95-D4/def2-QZVPPD level of theory. Please note that adding solvation contributions resulted in a non-smooth PES curve, which is why only gas-phase electronic energies were plotted in this Figure. Bold letters mark important stationary points on the PES. B: Molecular structures corresponding to the stationary points in A. Markedly, both "TS$_{rot}$" transition states feature carbonyl groups of the acyl ligand that exhibit near-planarity with the equatorial plane of the NHC ligands and the remaining equatorial CO ligand. Optimizing C, E, and F yielded proper transition states, albeit with very small imaginary eigenmodes that nonetheless showed the correct chemical motion of atomic groups. Final Gibbs free energies at the PW6B95-D4/def2-QZVPPD/COSMO-RS(THF) level of theory are provided in kcal mol^{-1} for all species. Notation as used in the main text, with the nature of ^{n}Bu$^-$ binding denoted as ^{n}Bu (Bu) or acyl (Ac): axial-bottom (axbot), equatorial (eq).

VII. Additional Computational Results

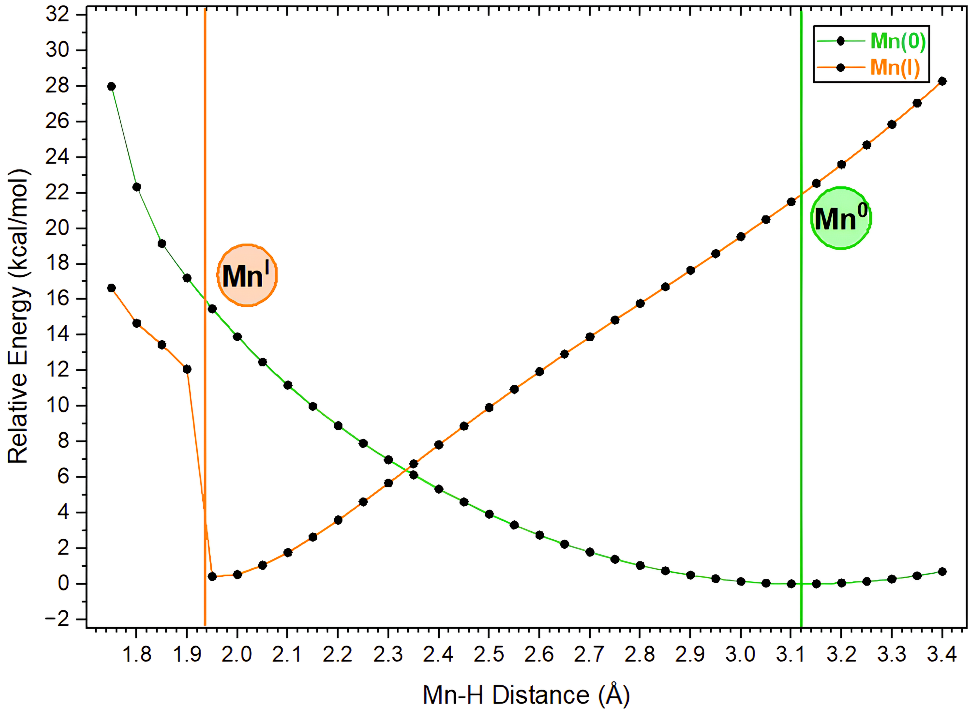

Figure S43. PES scan along the Mn—H bond length for 1^{H} (orange, Mn^{I}) and $[2^{H}]^{-}$ (green, Mn^{0}) with relative, solvated, electronic energies at the B3LYP-3c/CPCM(THF) level of theory. The vertical lines indicate the computed equilibrium bond lengths for each manganese oxidation state. Hence, e.g., constraining the Mn^{0} bond length to 1.93 Å introduces a pronounced energy penalty, which decreases **smoothly** as the bond is relaxed towards its dissociated equilibrium distance.

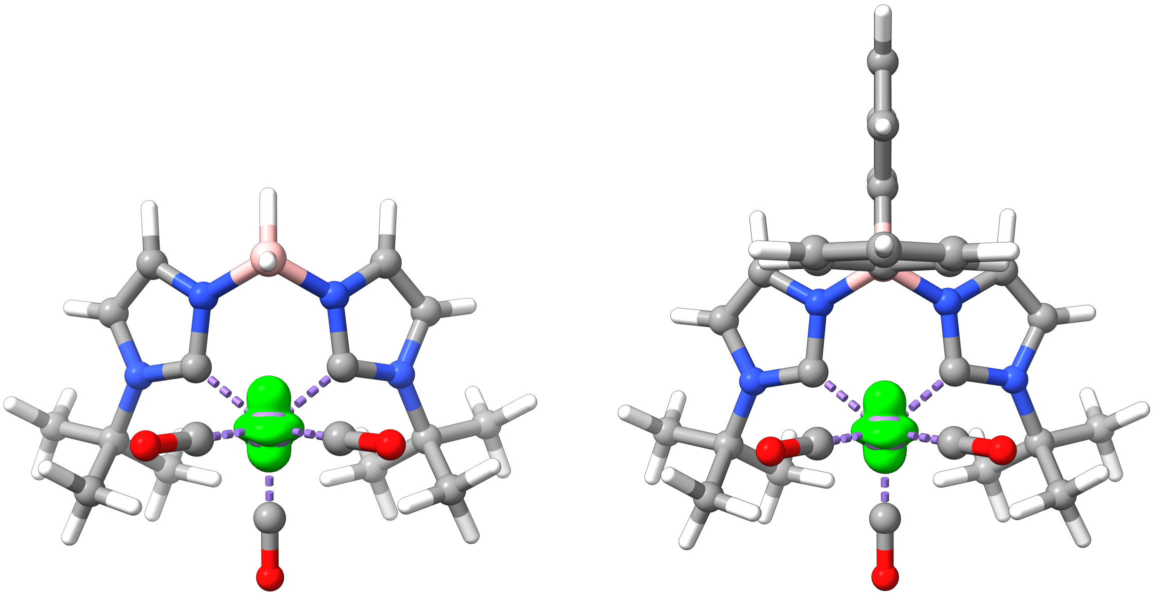

(a) (b)

Figure S44. Spin density plots (green) for $[2^{H}]^{-}$ (a) and $[2^{Ph}]^{-}$ (b) at the PW6B95-D4/def2-QZVPPD/COSMO-RS(THF) // B3LYP-3c/CPCM(THF) level of theory with a chosen isovalue of 0.03 Å^{-3}. Both spin density plots nicely reproduce the expected radical character on the metal center.

VII.I Potential Solvent Binding

Solvent binding of THF has been reported for similar manganese complexes before.[91] Hence, we investigated a few selected binding modes of THF when coordinated to $[1\text{-Ac}]^{-}$ as shown in Figure S45. Both $[1\text{-Ac}_{ax}(\text{THF})]^{-}$ and $[1\text{-Ac}_{eq}(\text{THF})_{bot}]^{-}$ rearranged to $[1\text{-Ac}_{axbot}(\text{THF})]^{-}$ and $[1\text{-Ac}_{eq}(\text{THF})]^{-}$ during the conformational sampling, respectively, and $[1\text{-Ac}_{eq}(\text{THF})]^{-}$ also turned out to be unstable as THF dissociated in the final geometry optimizations, even when pre-optimized first with a constrained Mn-O(THF) bond length before the final structural relaxation. This ultimately yielded only one local-minimum geometry with THF binding. However, even $[1\text{-Ac}_{axbot}(\text{THF})]^{-}$ displayed a large exergonic change for the free energy of THF dissociation ($-19.9\,\text{kcal mol}^{-1}$). Consequently, THF binding seems highly unlikely for all investigated species herein, which we rationalize by the required cleavage of the Mn-H agostic interaction upon THF binding, which is also accompanied by increased steric repulsion due to the crowded metal center.

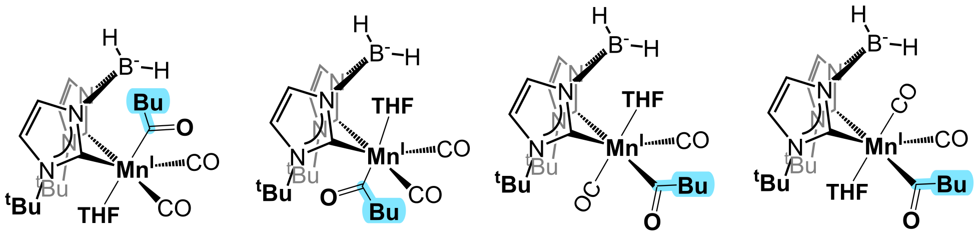

[1-Ac$_{ax}$(THF)]$^-$ **[1-Ac$_{axbot}$(THF)]$^-$** **[1-Ac$_{eq}$(THF)]$^-$** **[1-Ac$_{eq}$(THF$_{bot}$)]$^-$**

Figure S45. Structural representations of all investigated complexes with solvent coordination. Notation as used in the main text, with the nature of ^{n}Bu$^-$ binding denoted as ^{n}Bu (Bu) or acyl (Ac): axial-bottom (axbot), equatorial (eq).

VIII. List of Abbreviations

ALPB	analytical linearized Poisson-Boltzmann
AMAX	absolute maximal error
axbot	axial-bottom
BS	broken-symmetry
CE	conformer ensemble
CEH	charge extended Hückel
CENSO	command-line energetic sorting
COSMO-RS	conductor like screening model for real solvents
CPCM	conductor-like polarizable continuum model
CREST	conformer–rotamer ensemble sampling tool
DFA	density functional approximation
DFT	density functional theory
DRACO	dynamic radii adjustment for continuum solvation
eq	equatorial
Fc^+/Fc	ferrocenium/ferrocene redox-couple
GFN	geometries, vibrational frequencies and noncovalent interactions
GOAT	global optimization algorithm
HS	high-spin
IR	infrared
IRC	intrinsic reaction coordinate
KIE	kinetic isotope effect
LS	low-spin
MAE	mean absolute error
MAX	maximal error
MDN	median error
ME	mean error
Me	methyl
MeCN	acetonitrile
MIN	minimal error
MO	molecular orbital
mRRHO	modified rigid-rotor harmonic-oscillator
NEB	nudged elastic band
PES	potential energy surface
RMSE	root mean square error
SCF	self-consistent field
SD	standard deviation

SMD	solvation model based on the solute electron density
SP	spin-projected/spin projection
SPH	single-point Hessian
THF	tetrahydrofuran
TS	transition state
XRD	X-ray diffraction
xTB	extended tight-binding

IX. References

[20] S. Grimme, "Supramolecular Binding Thermodynamics by Dispersion-Corrected Density Functional Theory", *Chem. Eur. J.* **2012**, *18*, 9955–9964.

[21] S. Grimme, C. Bannwarth, P. Shushkov, "A Robust and Accurate Tight-Binding Quantum Chemical Method for Structures, Vibrational Frequencies, and Noncovalent Interactions of Large Molecular Systems Parametrized for All Spd-Block Elements (Z = 1–86)", *J. Chem. Theory Comput.* **2017**, *13*, 1989–2009.

[22] S. Ehlert, M. Stahn, S. Spicher, S. Grimme, "Robust and Efficient Implicit Solvation Model for Fast Semiempirical Methods", *J. Chem. Theory Comput.* **2021**, *17*, 4250–4261.

[23] D. R. Lide, C. R. Company (Eds.), *CRC Handbook of Chemistry and Physics 90th ed.*, CRC Press, Boca Raton, FL, **2009**.

[24] C. Bannwarth, E. Caldeweyher, S. Ehlert, A. Hansen, P. Pracht, J. Seibert, S. Spicher, S. Grimme, "Extended Tight-Binding Quantum Chemistry Methods", *WIREs Comput Mol Sci.* **2021**, *11*, e1493.

[25] C. Bannwarth, S. Ehlert, S. Grimme, "GFN2-xTB—An Accurate and Broadly Parametrized Self-Consistent Tight-Binding Quantum Chemical Method with Multipole Electrostatics and Density-Dependent Dispersion Contributions", *J. Chem. Theory Comput.* **2019**, *15*, 1652–1671.

[26] S. Grimme, A. Hansen, S. Ehlert, J.-M. Mewes, "r^{2}SCAN-3c: A "Swiss Army Knife" Composite Electronic-Structure Method", *J. Chem. Phys.* **2021**, *154*, 064103.

[27] V. Barone, M. Cossi, "Quantum Calculation of Molecular Energies and Energy Gradients in Solution by a Conductor Solvent Model", *J. Phys. Chem. A* **1998**, *102*, 1995–2001.

[28] M. Cossi, N. Rega, G. Scalmani, V. Barone, "Energies, Structures, and Electronic Properties of Molecules in Solution with the C-PCM Solvation Model", *J. Comput. Chem.* **2003**, *24*, 669.

[29] S. Grimme, "Exploration of Chemical Compound, Conformer, and Reaction Space with Meta-Dynamics Simulations Based on Tight-Binding Quantum Chemical Calculations", *J. Chem. Theory Comput.* **2019**, *15*, 2847–2862.

[30] P. Pracht, F. Bohle, S. Grimme, "Automated Exploration of the Low-Energy Chemical Space with Fast Quantum Chemical Methods", *Phys. Chem. Chem. Phys.* **2020**, *22*, 7169–7192.

[31] P. Pracht, S. Grimme, C. Bannwarth, F. Bohle, S. Ehlert, G. Feldmann, J. Gorges, M. Müller, T. Neudecker, C. Plett, S. Spicher, P. Steinbach, P. A. Wesołowski, F. Zeller, "CREST—A Program for the Exploration of Low-Energy Molecular Chemical Space", *J. Chem. Phys.* **2024**, *160*, 114110.

[32] B. De Souza, "GOAT: A Global Optimization Algorithm for Molecules and Atomic Clusters", *Angew. Chem. Int. Ed.* **2025**, *64*, e202500393.

[33] D. S. Tresp, T. K. Schramm, S. Luhach, A. Rosendo, A. Houn, T. Zhang, A. Turtz, J. V. Lockard, A. Hansen, D. E. Prokopchuk, "Expanding the PCET Thermochemistry of Cp^{N3}: N–H Bond Strengths of Metal-Free Cp^{N3} Molecules and the Influence of $Fe(CO)_3$ Coordination", *ACS Org. Inorg. Au* **2025**, *5*, 373–384.

[34] L. Lin, T. K. Schramm, P. Kucheryavy, R. A. Lalancette, A. Hansen, D. E. Prokopchuk, "A 100,000-Fold Increase in C–H Bond Acidity Gives Palladium a Key Advantage in $C(Sp^3$)–H Activation Compared to Nickel", *J. Am. Chem. Soc.* **2025**, *147*, 34395–34410.

[35] P. Pyykkö, M. Atsumi, "Molecular Single-Bond Covalent Radii for Elements 1–118", *Chem. Eur. J.* **2009**, *15*, 186–197.

[36] S. Grimme, F. Bohle, A. Hansen, P. Pracht, S. Spicher, M. Stahn, "Efficient Quantum Chemical Calculation of Structure Ensembles and Free Energies for Nonrigid Molecules", *J. Phys. Chem. A* **2021**, *125*, 4039–4054.

[37] A. V. Marenich, C. J. Cramer, D. G. Truhlar, "Universal Solvation Model Based on Solute Electron Density and on a Continuum Model of the Solvent Defined by the Bulk Dielectric Constant and Atomic Surface Tensions", *J. Phys. Chem. B* **2009**, *113*, 6378–6396.

[38] S. Spicher, S. Grimme, "Single-Point Hessian Calculations for Improved Vibrational Frequencies and Rigid-Rotor-Harmonic-Oscillator Thermodynamics", *J. Chem. Theory Comput.* **2021**, *17*, 1701–1714.

[39] F. Neese, "The SHARK Integral Generation and Digestion System", *J. Comput. Chem.* **2023**, *44*, 381–396.

[40] F. Neese, "Software Update: The ORCA Program System—Version 6.0", *WIREs Comput Mol Sci.* **2025**, *15*, e70019.

[41] X. Liu, K. Spiekermann, A. Menon, W. H. Green, M. Head-Gordon, "Revisiting a Large and Diverse Data Set for Barrier Heights and Reaction Energies: Best Practices in Density Functional Theory Calculations for Chemical Kinetics", *Phys. Chem. Chem. Phys.* **2025**, 13326–13339.

[42] P. Pracht, D. F. Grant, S. Grimme, "Comprehensive Assessment of GFN Tight-Binding and Composite Density Functional Theory Methods for Calculating Gas-Phase Infrared Spectra", *J. Chem. Theory Comput.* **2020**, *16*, 7044–7060.

[43] J. C. Zapata Trujillo, L. K. McKemmish, "VIBFREQ1295: A New Database for Vibrational Frequency Calculations", *J. Phys. Chem. A* **2022**, *126*, 4100–4122.

[44] J. C. Zapata Trujillo, L. K. McKemmish, "Model Chemistry Recommendations for Scaled Harmonic Frequency Calculations: A Benchmark Study", *J. Phys. Chem. A* **2023**, *127*, 1715–1735.

[45] G. Mills, H. Jónsson, G. K. Schenter, "Reversible Work Transition State Theory: Application to Dissociative Adsorption of Hydrogen", *Surf. Sci.* **1995**, *324*, 305–337.

[46] V. Ásgeirsson, B. O. Birgisson, R. Bjornsson, U. Becker, F. Neese, C. Riplinger, H. Jónsson, "Nudged Elastic Band Method for Molecular Reactions Using Energy-Weighted Springs Combined with Eigenvector Following", *J. Chem. Theory Comput.* **2021**, *17*, 4929–4945.

[47] K. Fukui, "Formulation of the Reaction Coordinate", *J. Phys. Chem.* **1970**, *74*, 4161–4163.

[48] K. Ishida, K. Morokuma, A. Komornicki, "The Intrinsic Reaction Coordinate. An *a b i n i t i o* Calculation for HNC→HCN and H-+CH4→CH4+H-", *J. Chem. Phys.* **1977**, *66*, 2153–2156.

[49] Y. Zhao, D. G. Truhlar, "Design of Density Functionals That Are Broadly Accurate for Thermochemistry, Thermochemical Kinetics, and Nonbonded Interactions", *J. Phys. Chem. A* **2005**, *109*, 5656–5667.

[50] F. Weigend, F. Furche, R. Ahlrichs, "Gaussian Basis Sets of Quadruple Zeta Valence Quality for Atoms H–Kr", *J. Chem. Phys.* **2003**, *119*, 12753–12762.

[51] F. Weigend, R. Ahlrichs, "Balanced Basis Sets of Split Valence, Triple Zeta Valence and Quadruple Zeta Valence Quality for H to Rn: Design and Assessment of Accuracy", *Phys. Chem. Chem. Phys.* **2005**, *7*, 3297–3305.

[52] F. Neese, F. Wennmohs, A. Hansen, U. Becker, "Efficient, Approximate and Parallel Hartree–Fock and Hybrid DFT Calculations. A 'Chain-of-Spheres' Algorithm for the Hartree–Fock Exchange", *Chem. Phys.* **2009**, *356*, 98–109.

[53] B. Helmich-Paris, B. De Souza, F. Neese, R. Izsák, "An Improved Chain of Spheres for Exchange Algorithm", *J. Chem. Phys.* **2021**, *155*, 104109.

[54] F. Weigend, "Hartree–Fock Exchange Fitting Basis Sets for H to Rn", *J. Comput. Chem.* **2008**, *29*, 167–175.

[55] E. R. Davidson, "The Iterative Calculation of a Few of the Lowest Eigenvalues and Corresponding Eigenvectors of Large Real-Symmetric Matrices", *J. Chem. Phys.* **1975**, *17*, 87–94.

[56] R. Seeger, J. A. Pople, "Self-Consistent Molecular Orbital Methods. XVIII. Constraints and Stability in Hartree–Fock Theory", *J. Chem. Phys.* **1977**, *66*, 3045–3050.

[57] R. Bauernschmitt, R. Ahlrichs, "Stability Analysis for Solutions of the Closed Shell Kohn–Sham Equation", *J. Chem. Phys.* **1996**, *104*, 9047–9052.

[58] M. Bursch, H. Neugebauer, S. Ehlert, S. Grimme, "Dispersion Corrected r^2SCAN Based Global Hybrid Functionals: r^2SCANh, r^2SCAN0, and r^2SCAN50", *J. Chem. Phys.* **2022**, *156*, 134105.

[59] E. Caldeweyher, C. Bannwarth, S. Grimme, "Extension of the D3 Dispersion Coefficient Model", *J. Chem. Phys.* **2017**, *147*, 034112.

[60] E. Caldeweyher, S. Ehlert, A. Hansen, H. Neugebauer, S. Spicher, C. Bannwarth, S. Grimme, "A Generally Applicable Atomic-Charge Dependent London Dispersion Correction", *J. Chem. Phys.* **2019**, *150*, 154122.

[61] V. N. Staroverov, G. E. Scuseria, J. Tao, J. P. Perdew, "Comparative Assessment of a New Nonempirical Density Functional: Molecules and Hydrogen-Bonded Complexes", *J. Chem. Phys.* **2003**, *119*, 12129–12137.

[62] C. Lee, W. Yang, R. G. Parr, "Development of the Colle-Salvetti Correlation-Energy Formula into a Functional of the Electron Density", *Phys. Rev. B* **1988**, *37*, 785.

[63] A. D. Becke, "Density-Functional Exchange-Energy Approximation with Correct Asymptotic Behavior", *Phys. Rev. A* **1988**, *38*, 3098.

[64] C. Adamo, V. Barone, "Toward Reliable Density Functional Methods without Adjustable Parameters: The PBE0 Model", *J. Chem. Phys.* **1999**, *110*, 6158–6170.

[65] N. Mardirossian, M. Head-Gordon, "ωB97X-V: A 10-Parameter, Range-Separated Hybrid, Generalized Gradient Approximation Density Functional with Nonlocal Correlation, Designed by a Survival-of-the-Fittest Strategy", *Phys. Chem. Chem. Phys.* **2014**, *16*, 9904–9924.

[66] O. A. Vydrov, T. Van Voorhis, "Nonlocal van Der Waals Density Functional: The Simpler the Better", *J. Chem. Phys.* **2010**, *133*, 244103.

[67] M. Müller, A. Hansen, S. Grimme, "ωB97X-3c: A Composite Range-Separated Hybrid DFT Method with a Molecule-Optimized Polarized Valence Double-ζ Basis Set", *J. Chem. Phys.* **2023**, *158*, 014103.

[68] N. Mardirossian, M. Head-Gordon, "ω B97M-V: A Combinatorially Optimized, Range-Separated Hybrid, Meta-GGA Density Functional with VV10 Nonlocal Correlation", *J. Chem. Phys.* **2016**, *144*, 214110.

[69] M. Friede, S. Ehlert, S. Grimme, J.-M. Mewes, "Do Optimally Tuned Range-Separated Hybrid Functionals Require a Reparametrization of the Dispersion Correction? It Depends", *J. Chem. Theory Comput.* **2023**, *19*, 8097–8107.

[70] A. Klamt, "Conductor-like Screening Model for Real Solvents: A New Approach to the Quantitative Calculation of Solvation Phenomena", *J. Phys. Chem.* **1995**, *99*, 2224–2235.

[71] A. Klamt, V. Jonas, T. Bürger, J. C. W. Lohrenz, "Refinement and Parametrization of COSMO-RS", *J. Phys. Chem. A* **1998**, *102*, 5074–5085.

[72] A. Klamt, "The COSMO and COSMO-RS Solvation Models", *WIREs Comput Mol Sci.* **2011**, *1*, 699–709.

[73] Y. J. Franzke, C. Holzer, J. H. Andersen, T. Begušić, F. Bruder, S. Coriani, F. Della Sala, E. Fabiano, D. A. Fedotov, S. Fürst, S. Gillhuber, R. Grotjahn, M. Kaupp, M. Kehry, M. Krstić, F. Mack, S. Majumdar, B. D. Nguyen, S. M. Parker, F. Pauly, A. Pausch, E. Perlt, G. S. Phun, A. Rajabi, D. Rappoport, B. Samal, T. Schrader, M. Sharma, E. Tapavicza, R. S. Treß, V. Voora, A. Wodyński, J. M. Yu, B. Zerulla, F. Furche, C. Hättig, M. Sierka, D. P. Tew, F. Weigend, "TURBOMOLE: Today and Tomorrow", *J. Chem. Theory Comput.* **2023**, *19*, 6859–6890.

[74] C. J. Cramer, *Essentials of Computational Chemistry: Theories and Models 2nd ed.*, Wiley, Chichester, West Sussex, England; Hoboken, NJ, **2004**.

[75] O. Hammerich, B. Speiser (Eds.), *Organic Electrochemistry: Revised and Expanded 5th ed.*, CRC Press, Boca Raton, FL, **2015**.

[76] J. Ho, "Are Thermodynamic Cycles Necessary for Continuum Solvent Calculation of pKas and Reduction Potentials?", *Phys. Chem. Chem. Phys.* **2015**, *17*, 2859–2868.

[77] J. Ho, M. Z. Ertem, "Calculating Free Energy Changes in Continuum Solvation Models", *J. Phys. Chem. B* **2016**, *120*, 1319–1329.

[78] E. F. Pettersen, T. D. Goddard, C. C. Huang, G. S. Couch, D. M. Greenblatt, E. C. Meng, T. E. Ferrin, "UCSF Chimera—A Visualization System for Exploratory Research and Analysis", *J. Comput. Chem.* **2004**, *25*, 1605–1612.

[79] E. F. Pettersen, T. D. Goddard, C. C. Huang, E. C. Meng, G. S. Couch, T. I. Croll, J. H. Morris, T. E. Ferrin, "UCSF ChimeraX : Structure Visualization for Researchers, Educators, and Developers", *Protein Sci.* **2021**, *30*, 70–82.

[80] S. Grimme, J. Antony, S. Ehrlich, H. Krieg, "A Consistent and Accurate *Ab Initio* Parametrization of Density Functional Dispersion Correction (DFT-D) for the 94 Elements H-Pu", *J. Chem. Phys.* **2010**, *132*, 154104.

[81] S. Grimme, S. Ehrlich, L. Goerigk, "Effect of the Damping Function in Dispersion Corrected Density Functional Theory", *J. Comput. Chem.* **2011**, *32*, 1456.

[82] S. Grimme, "Accurate Calculation of the Heats of Formation for Large Main Group Compounds with Spin-Component Scaled MP2 Methods", *J. Phys. Chem. A* **2005**, *109*, 3067–3077.

[83] R. Sure, S. Grimme, "Corrected Small Basis Set Hartree-Fock Method for Large Systems", *J. Comput. Chem.* **2013**, *34*, 1672–1685.

[84] J. G. Brandenburg, C. Bannwarth, A. Hansen, S. Grimme, "B97-3c: A Revised Low-Cost Variant of the B97-D Density Functional Method", *J. Chem. Phys.* **2018**, *148*, 064104.

[85] S. Grimme, J. G. Brandenburg, C. Bannwarth, A. Hansen, "Consistent Structures and Interactions by Density Functional Theory with Small Atomic Orbital Basis Sets", *J. Chem. Phys.* **2015**, *143*, 054107.

[86] A. Karagiannis, H. Neugebauer, R. A. Lalancette, S. Grimme, A. Hansen, D. E.

Prokopchuk, "Pushing the Limits of Organometallic Redox Chemistry with an Isolable Mn(-I) Dianion", *J. Am. Chem. Soc.* **2024**, *146*, 19279–19285.

[87] H. Neugebauer, H. T. Vuong, J. L. Weber, R. A. Friesner, J. Shee, A. Hansen, *Toward Benchmark-quality Ab Initio Predictions for 3d Transition Metal Electrocatalysts - A Comparison of CCSD(T) and Ph-AFQMC*, Preprint, Chemistry, **2023**.

[88] F. Neese, "Prediction of Molecular Properties and Molecular Spectroscopy with Density Functional Theory: From Fundamental Theory to Exchange-Coupling", *Coord. Chem. Rev.* **2009**, *253*, 526–563.

[89] K. Yamaguchi, F. Jensen, A. Dorigo, K. Houk, "A Spin Correction Procedure for Unrestricted Hartree-Fock and Møller-Plesset Wavefunctions for Singlet Diradicals and Polyradicals", *Chem. Phys. Lett.* **1988**, *149*, 537–542.

[90] Y. Kitagawa, T. Saito, K. Yamaguchi in *Symmetry (Group Theory) and Mathematical Treatment in Chemistry*, T. Akitsu (Ed.), InTech, **2018**.

[91] T. Zhou, S. Malakar, S. L. Webb, K. Krogh-Jespersen, A. S. Goldman, "Polar Molecules Catalyze CO Insertion into Metal-Alkyl Bonds through the Displacement of an Agostic C-H Bond", *Proc. Natl. Acad. Sci. U.S.A.* **2019**, *116*, 3419–3424.
